# Gold(I)-catalyzed 6-*endo* hydroxycyclization of 7-substituted-1,6-enynes

**DOI:** 10.3762/bjoc.9.263

**Published:** 2013-10-29

**Authors:** Ana M Sanjuán, Alberto Martínez, Patricia García-García, Manuel A Fernández-Rodríguez, Roberto Sanz

**Affiliations:** 1 Área de Química Orgánica, Departamento de Química, Facultad de Ciencias, Universidad de Burgos, Pza. Misael Bañuelos s/n, 09001 Burgos, Spain

**Keywords:** catalysis, dihydronaphthalenes, gold, gold catalysis, hydroxycyclization, selectivity

## Abstract

The cyclization of *o*-(alkynyl)-3-(methylbut-2-enyl)benzenes, 1,6-enynes having a condensed aromatic ring at C3–C4 positions, has been studied under the catalysis of cationic gold(I) complexes. The selective 6-*endo*-*dig* mode of cyclization observed for the 7-substituted substrates in the presence of water or methanol giving rise to hydroxy(methoxy)-functionalized dihydronaphthalene derivatives is highly remarkable in the context of the observed reaction pathways for the cycloisomerizations of 1,6-enynes bearing a trisubstituted olefin.

## Introduction

**Scheme 1:**
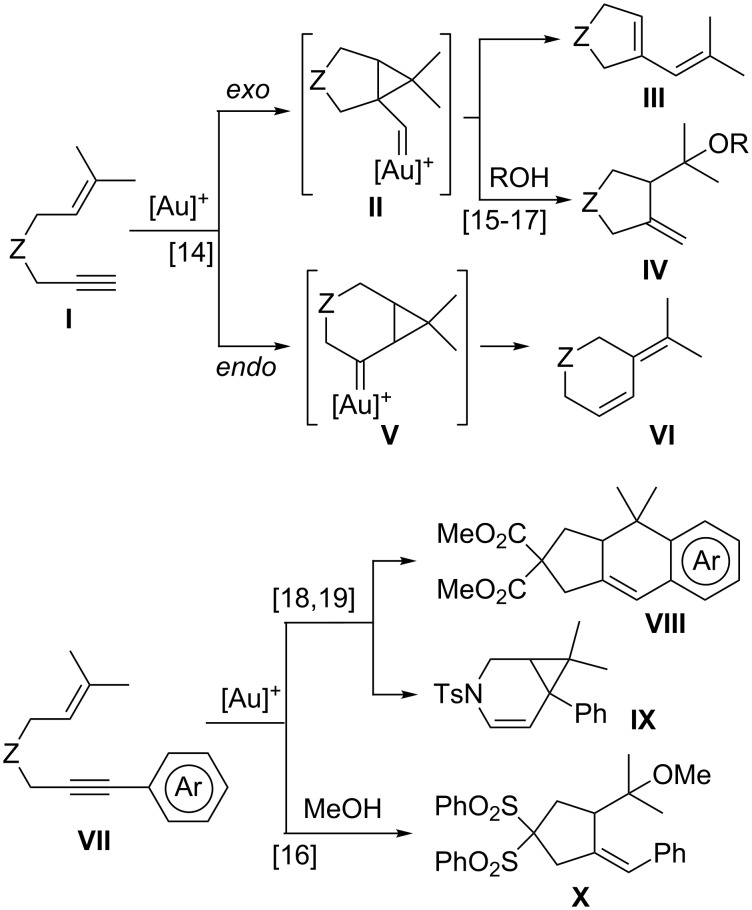
Gold(I)-catalyzed reactions of 1,6-enynes.

The cycloisomerization reactions of enynes catalyzed by gold complexes are a powerful tool for accessing complex products from rather simple starting materials under soft and straightforward conditions [1–4]. In this context, 1,6-enynes have been extensively studied, mainly by Echavarren and co-workers, as substrates in the identification of new reactivities catalyzed by gold and other transition metal complexes [5–13]. Cyclopropyl metal carbenes **II** are usually formed by *exo-dig* processes from enynes **I** bearing a terminal alkyne, which in the absence of external nucleophiles undergo skeletal rearrangements to afford products such as **III** (single cleavage) [14]. However, reactions of **II** with alcohols or water give the corresponding products of alkoxy(hydroxy)cyclization **IV** [15–17] (Scheme 1). The less common 6-*endo* cyclization via metal carbenes **V** was also observed in particular cases affording methylenecyclohexene derivatives like **VI** [14]. On the other hand, 1,6-enynes **VII**, bearing an aryl substituent at the alkyne, undergo a formal intramolecular [4 + 2] cycloaddition through an initial 5-*exo* cyclization followed by a Friedel–Crafts-type reaction to cyclopenta[*b*]naphthalenes **VIII** or, alternatively, a 6-*endo* cyclization to bicyclo[4.1.0]hept-4-enes like **IX** [18–19] (Scheme 1). In the case that MeOH is present a 5-*exo* methoxycyclization is observed, e.g., in the formation of **X** resembling the behaviour of **I** [16,20]. In addition, the gold-catalyzed reaction of 7-phenyl-1,6-enynes with a terminal double bond gives rise to bicyclo[3.2.0]heptene derivatives [19,21].

**Scheme 2:**
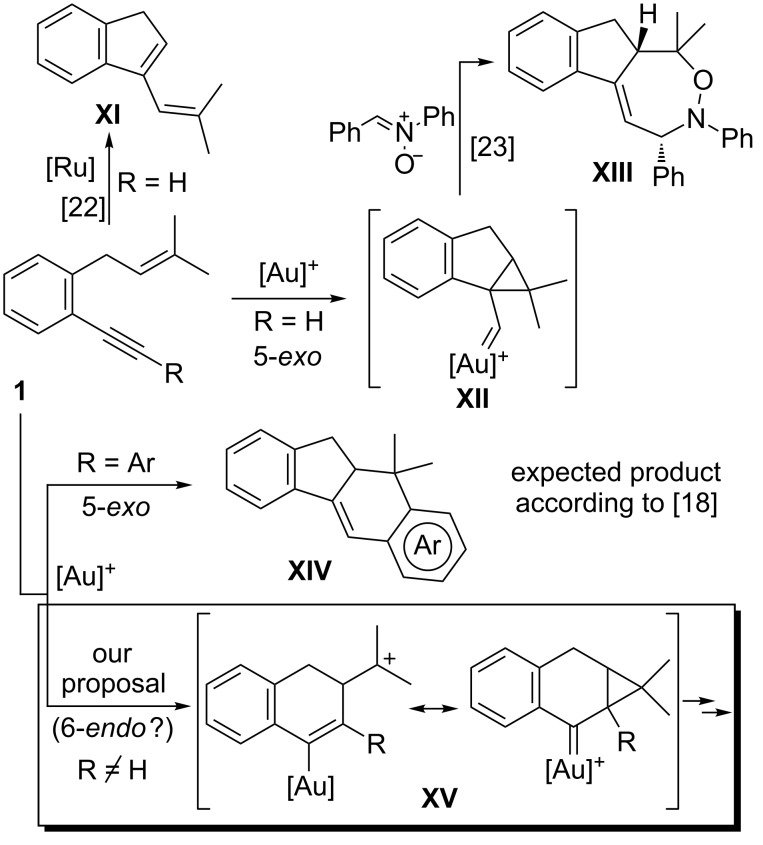
Cyclization of *o*-(alkynyl)-(3-methylbut-2-enyl)benzenes **1**. Previous work and proposed pathways.

Despite the numerous studies about the metal-catalyzed transformations of 1,6-enynes, *o*-(alkynyl)-(3-methylbut-2-enyl)benzenes **1** that are also 1,6-enynes bearing an attached aryl ring at the C3–C4 positions, have been scarcely studied. Only Liu and co-workers have reported the behaviour of terminal substrates **1** (R = H) under ruthenium catalysis, which afford the corresponding metathesis-type product **XI** [22] (Scheme 2). More recently, the same authors have described the gold-catalyzed [2 + 2 + 3] cycloaddition reaction of these compounds with nitrones giving rise to functionalized 1,2-oxazepane derivatives **XIII**. This cascade process takes place through the interception of the 1,4-dipole equivalent **XII** generated by an initial 5-*exo* cyclization, although with some gold catalysts minor amounts of **XI** were also obtained [23] (Scheme 2). Following our interest in the development of new gold-catalyzed reactions [24–31], in this context we thought that it could be interesting to study if the cyclization of easily available compounds **1** bearing an internal acetylene moiety would take place through an initial 5-*exo* cyclization that in the case of aryl-substituted enynes (R = Ar) would give rise to a formal [4 + 2] cycloaddition product **XIV** [18–19], or alternatively, through a relatively less common 6-*endo*-*dig* pathway via gold species **XV**, which could be represented as two resonance structures highlighting both the carbocation or carbenoid nature of this intermediate (Scheme 2).

## Results and Discussion

**Scheme 3:**
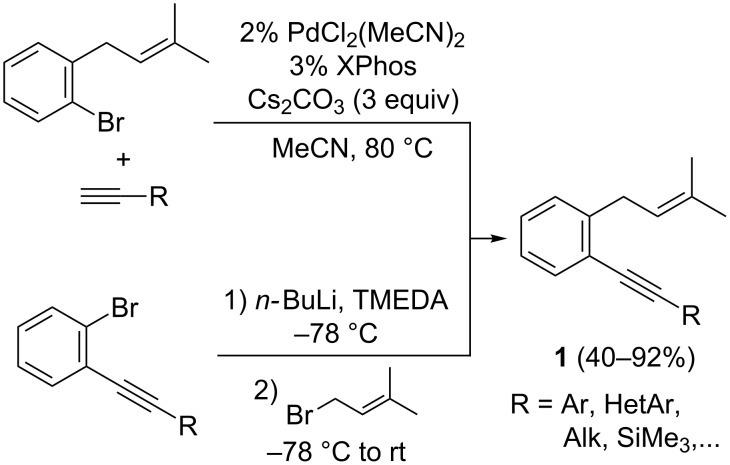
Synthesis of *o*-(alkynyl)-(3-methylbut-2-enyl)benzenes **1**.

As established in Scheme 2, we were intrigued by the possibility that *o*-(alkynyl)-(3-methylbut-2-enyl)benzenes **1** could undergo a 6-*endo*-*dig* cyclization in the presence of cationic gold(I) complexes instead of the usually more favoured 5-*exo*-*dig* pathway. So, we initially prepared a variety of these *o*-disubstituted benzene derivatives **1** by two approaches (see Supporting Information File 1) (Scheme 3). First, *o*-(bromo)-3-(methylbut-2-enyl)benzene was prepared by the reaction of commercially available 2-methyl-1-propenylmagnesium bromide with 2-bromobenzyl bromide in the presence of CuI and 2,2’-bipyridyl [32]. This aryl bromide could be coupled with selected terminal alkynes by using cesium carbonate as a base and PdCl_2_(MeCN)_2_/XPhos as a catalytic system [33]. Alternatively, several *o*-(alkynyl)bromobenzenes [34] could be transformed into the corresponding derivatives **1** by bromine–lithium exchange and further treatment with 3,3-dimethylallyl bromide in the presence of TMEDA [23].

**Scheme 4:**
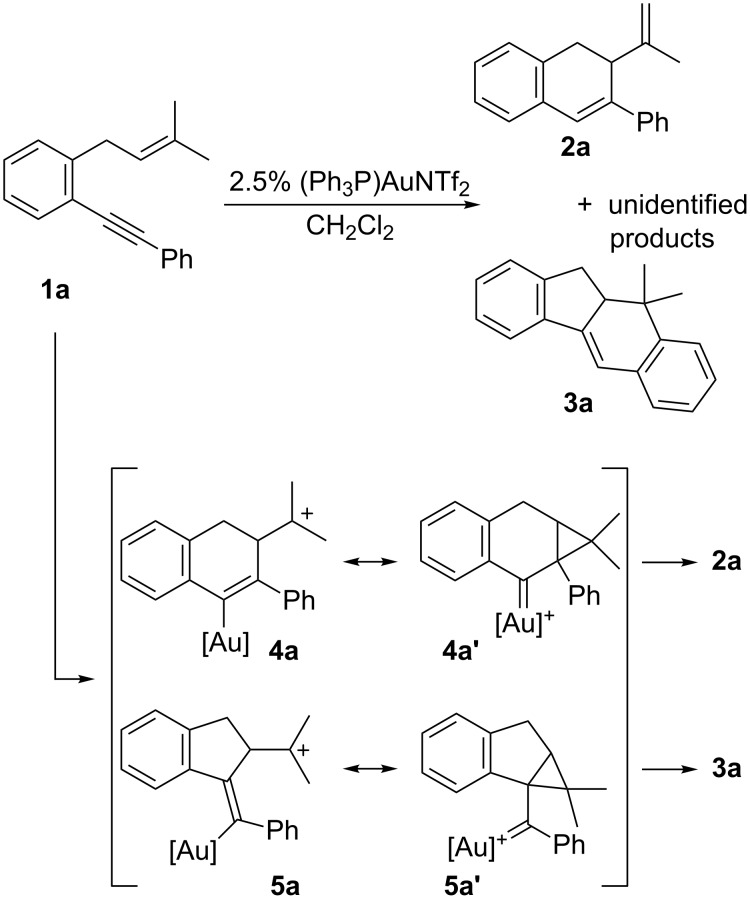
Gold(I)-catalyzed cycloisomerization of **1a**.

We selected 1-(2-(2-(3-methylbut-2-enyl)phenyl)ethynyl)benzene (**1a**) as model substrate for the initial experiments (Scheme 4). Its reaction with (Ph_3_P)AuNTf_2_, reported by Gagosz and co-workers as a very active catalyst for the cycloisomerization of closely related 7-aryl-1,6-enynes [35], gave rise to a ca. 3:1 mixture of dihydronaphthalene derivative **2a** and tetracyclic compound **3a** along with some other unidentified minor products. The two major products resulted to be inseparable by column chromatography and were isolated in 68% overall yield. It is remarkable that compound **2a**, derived from a 6-*endo* cyclization and further proton elimination from intermediate resonance structures **4a** and **4a’**, is generated in preference to **3a** which would be the expected product derived from a formal [4 + 2] cycloaddition initiated by a 5-*exo* cyclization followed by a Friedel–Crafts-type process in intermediate **5a** or **5a’**, as described by Echavarren and co-workers [18–19].

**Scheme 5:**
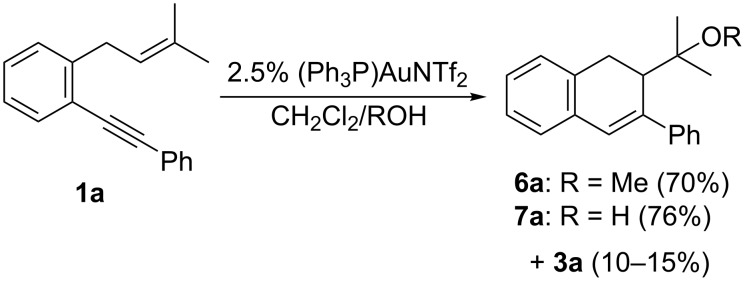
Initial experiments and proof of concept.

Prompted by this result and taking into account the reported results about the 5-*endo* hydroxy- and alkoxycyclization of 1,5-enynes [36], as well as our recent report about the alkoxycyclization of 1,3-dien-5-ynes [31], we wondered if the presence of an external protic nucleophile, such as methanol or water, could have an important influence on controlling the selectivity of the reaction. Encouragingly, when we treated model substrate **1a** with (Ph_3_P)AuNTf_2_ in a 10:1 mixture of CH_2_Cl_2_ and MeOH as the solvent, the methoxyalkyl-substituted derivative **6a** was obtained as the major product along with minor amounts of **3a** (ca. 6:1 ratio) (Scheme 5) [37]. Moreover, the use of H_2_O (20 equiv) also led to a high yield of the hydroxyalkyl-substituted dihydronaphthalene derivative **7a**, whose structure was further confirmed by X-ray analysis [38]. In both cases the high selectivity (>5:1) of these reactions for the 6-*endo*-type cyclization should be noted and only minor amounts (10–15%) of **3a** were also formed.

Due to the unexpected 6-*endo*-favored pathway found for substrate **1a** [39], we attempted to further improve this selectivity in the hydroxycyclization process (Table 1). Switching the ligand from Ph_3_P to XPhos or N-heterocyclic carbene (IPr) slightly decreases the selectivity for the 6-*endo* cyclization (Table 1, entry 1 vs entries 2 and 3). However, when the cationic gold complex (JohnPhos)(NCMe)AuSbF_6_, developed by Echavarren and co-workers [40], was employed as a catalyst a moderate increase in the ratio of **7a** vs **3a** was observed (Table 1, entry 4). Both cationic gold complexes (Ph_3_P)AuNTf_2_ and (JohnPhos)(NCMe)AuSbF_6_ gave rise to a similar yield of isolated alcohol **7a**. Changing the solvent from CH_2_Cl_2_ to a mixture containing other more polar solvent such as acetone or dioxane (Table 1, entries 5 and 6) did not have a significant influence on the selectivity but led to the formation of minor amounts of alcohol **8a**, derived from a 5-*exo* hydroxycyclization reaction. With a 1:1 mixture of CH_2_Cl_2_/dioxane the effect of the selected catalytic systems was checked (Table 1, entries 7–10). We found that the use of JohnPhos as a ligand and SbF_6_ as a counter ion (Table 1, entries 9 and 10) resulted in a slightly better selectivity, although trace amounts of alcohol **8a** were also generated, which make the isolation of **7a** more difficult. Overall, we concluded that both commercially available gold complexes (Ph_3_P)AuNTf_2_ and (JohnPhos)(NCMe)AuSbF_6_ lead to comparable good results in the 6-*endo* hydroxycyclization of **1a**. The type of products derived from the 5-*exo* pathway (**3a** and **8a**) depends on the solvent: in CH_2_Cl_2_ **3a** is mainly obtained, whereas the alcohol **8a** appears when a more polar mixture of solvents was used.

**Table 1:** Effect of the catalyst and reaction conditions on the hydroxycyclization of **1a**. 6-*Endo* vs 5-*exo* cyclization.^a^

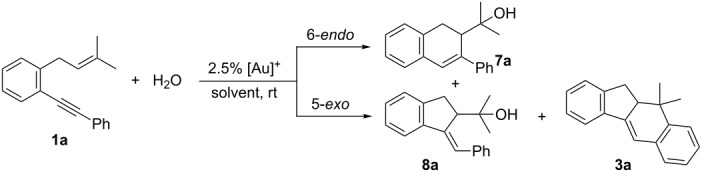				

Entry	Catalyst	Solvent	Ratio^b^ 6-*endo*/5-*exo*	Yield (%)^c^

1	(Ph_3_P)AuNTf_2_	CH_2_Cl_2_	5:1^d^	76
2	XPhosAuNTf_2_^e^	CH_2_Cl_2_	3:1^d^	—
3	IPrAuCl/AgSbF_6_^f^	CH_2_Cl_2_	4:1^d^	—
4	(JohnPhos)(NCMe)AuSbF_6_^g^	CH_2_Cl_2_	6:1^d^	77
5	(Ph_3_P)AuNTf_2_	CH_2_Cl_2_/Me_2_CO (1:1)	4.5:1^h^	—
6	(Ph_3_P)AuNTf_2_	CH_2_Cl_2_/dioxane (1:1)	5:1^h^	75^i^
7	XPhosAuNTf_2_^e^	CH_2_Cl_2_/dioxane (1:1)	3:1^h^	—
8	JohnPhosAuNTf_2_^g^	CH_2_Cl_2_/dioxane (1:1)	3:1^h^	—
9	(JohnPhos)(NCMe)AuSbF_6_^g^	CH_2_Cl_2_/dioxane (1:1)	7:1^h^	77^i^
10	JohnPhosAuCl/AgSbF_6_^g^	CH_2_Cl_2_/dioxane (1:1)	7:1^h^	—

^a^Reactions were carried out by treatment of **1a** (0.1 mmol) with H_2_O (2.2 mmol, 0.04 mL) in 0.4 mL of solvent until complete consumption of the starting material, as judged by GC–MS and/or TLC analysis (overnight). ^b^Determined by ^1^H NMR analysis of the crude reaction mixture. ^c^Isolated yield of **7a**. ^d^The 5-*exo* pathway gives rise to **3a**. ^e^XPhos = 2-dicyclohexylphosphino-2’,4’,6’-tri-isopropylbiphenyl. ^f^IPr = 1,3-bis-(2,6-di-isopropylphenyl)imidazol-2-ylidene. ^g^JohnPhos = 2-(di-*tert*-butylphosphino)biphenyl. ^h^A mixture of **3a** and **8a** was obtained through the 5-*exo* pathway. ^i^Approximately 5% of **8a** was also isolated.

Once we have selected the best conditions to favor the 6-*endo* hydroxycyclization reaction, a selection of substrates **1a–k**, bearing different groups at the triple bond, were reacted under the established conditions (Table 2). When aromatic or alkenyl groups are present as the substituents of the alkyne (Table 2, entries 1–7) the 6-*endo* cyclization takes place in selective or almost exclusively fashion allowing the isolation of 2-(1,2-dihydro-3-substituted naphthalen-2-yl)propan-2-ol derivatives **7** in usually high yields. Interestingly, we have also observed that when starting with enynes possessing an electron-rich aromatic ring or an alkenyl group at the C7-position of the 1,6-enyne the cyclization results almost completely selective via the 6-*endo* mode (Table 2, entries 2,3 and entries 6,7). However, in the case of halogen-containing aromatic substituents at C7 the formation of the corresponding products **3** or **8**, derived from an initial 5-*exo* cyclization, becomes more competitive (Table 2, entries 4 and 5). Then, we turned our attention to alkyl-substituted alkynes (Table 2, entries 8 and 9), which could not undergo the formal [4 + 2] cycloaddition leading to **3**. In these cases, and after some optimization studies, we surprisingly found that the solvent has an important role on the selectivity of the cyclization. When a 1:1 mixture of CH_2_Cl_2_/dioxane was used the 5-*exo* hydroxycyclization that gives rise to alcohols **8** was competitive with the 6-*endo* process (3:1 for **1h** and 1.7:1 for **1i**), allowing the isolation of the corresponding methyleneindene derivatives **8h** and **8i** in 21% and 30% yield, respectively [41]. Gratifyingly, we found that when the same reactions were performed in CH_2_Cl_2_ the 6-*endo* cyclization was completely selective leading to the corresponding alcohols **7** in high yields (Table 2, entries 8 and 9). On the other hand, the reaction of trimethylsilyl-substituted enyne **1j** did not proceed at all (Table 2, entry 10), whereas the presence of a phenylthio group as an R substituent mainly afforded the corresponding 6-*endo* product **7k** although the reaction was significantly slower (Table 2, entry 11). As expected [10–12] the terminal enyne **1l** (R = H) underwent exclusively the 5-*exo* cyclization leading to the corresponding alcohol **8l** in 55% yield (Table 2, entry 12).

**Table 2:** Synthesis of 2-(1,2-dihydro-3-substituted-naphthalen-2-yl)propan-2-ol derivatives **7** by gold-catalyzed 6-*endo* hydroxycyclization of enynes **1**.^a^

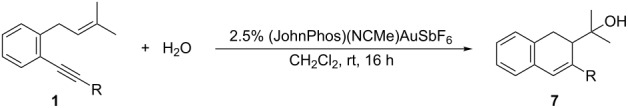				

Entry	Starting material	R	Product	Yield (%)^b^

1	**1a**	Ph	**7a**	77 (12)^c^
2	**1b**	4-MeOC_6_H_4_	**7b**	80
3	**1c**	2,4,5-(Me)_3_C_6_H_2_	**7c**	71
4	**1d**	3-ClC_6_H_4_	**7d**	63 (22)^d^
5	**1e**	2,4-(F)_2_C_6_H_3_	**7e**	75^e^
6	**1f**	thiophen-3-yl	**7f**	82
7	**1g**	*c*-C_6_H_9_	**7g**	79
8^f^	**1h**	*c*-C_3_H_5_	**7h**	77
9	**1i**	*n*-Bu	**7i**	82
10	**1j**	SiMe_3_	—	—^g^
11^h^	**1k**	SPh	**7k**	60
12	**1l**	H	**8l**	55

^a^Reactions were carried out by treatment of **1** (0.3 mmol) with H_2_O (22 equiv, 0.12 mL) in 1.2 mL of solvent until complete consumption of the starting material, as judged by GC–MS and/or TLC analysis (overnight). ^b^Isolated yield of compounds **7** after column chromatography. ^c^Yield of **3a** which could not be isolated in pure form. ^d^Isolated yield of **3d** which was obtained as a mixture of regioisomers with respect to the chlorine atom position. ^e^Isolated along with ≈10% of **8e**. ≈10% of **2e** is also observed. ^f^Carried out with (Ph_3_P)AuNTf_2_. Slightly lower yield (ca. 5%) was obtained with (JohnPhos)(NCMe)AuSbF_6_. ^g^Starting material was recovered. ^h^Reaction time: 48 h.

**Scheme 6:**
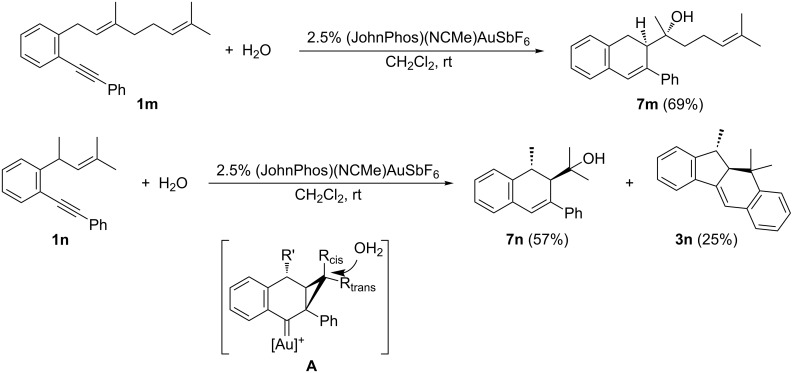
Gold(I)-catalyzed hydroxycyclization of enynes **1m**,**n**.

At this point we wondered if the cyclization would be diastereoselective, so we prepared enynes **1m** by reacting 2-(phenylethynyl)phenyllithium with geranyl bromide and **1n** by two Wittig reactions from 2’-(phenylethynyl)acetophenone (see Supporting Information File 1). First, the hydroxycyclization of **1m**, as a pure *E* isomer, under the previously established conditions afforded the dihydronaphthalene derivative **7m** as a single isomer (Scheme 6), whose relative configuration was assigned by analogy with previously related results reported by Gagosz and co-workers [37]. On the other hand, the reaction of **1n** afforded a ca. 2.5:1 mixture of alcohol **7n** [42] and the tetracyclic product **3n**, derived from an initial 5-*exo* cyclization and subsequent Friedel–Crafts reaction (Scheme 6). Both compounds were isolated as single stereoisomers with a high overall yield [43]. In this case, the 5-*exo* pathway was more competitive compared to the result of model substrate **1a**, probably due to the Thorpe–Ingold-type effect caused by the methyl group at the allylic position. To account for the stereoselectivity of these reactions we proposed the generation of a stabilized gold–carbenoid intermediate such as **A** that undergoes stereoselective attack by water (Scheme 6).

**Scheme 7:**
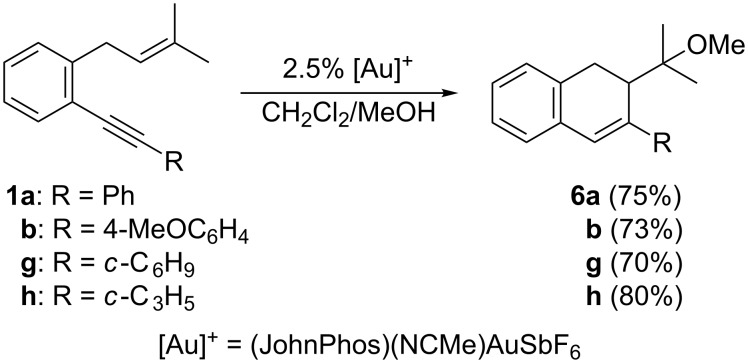
Gold(I)-catalyzed methoxycyclization of selected 1,6-enynes **1** [45].

Furthermore, we have also carried out the methoxycyclization of selected 1,6-enynes **1** by their treatment with catalytic amounts of (JohnPhos)(NCMe)AuSbF_6_ in a 30:1 mixture of CH_2_Cl_2_ and MeOH as the solvent (Scheme 7) [44]. The corresponding methoxy-functionalized dihydronaphthalene derivatives **6** were obtained in high yields although the corresponding minor isomer derived from a 5-*exo* cyclization could not be separated in the case of **6a** and **6h**.

**Scheme 8:**
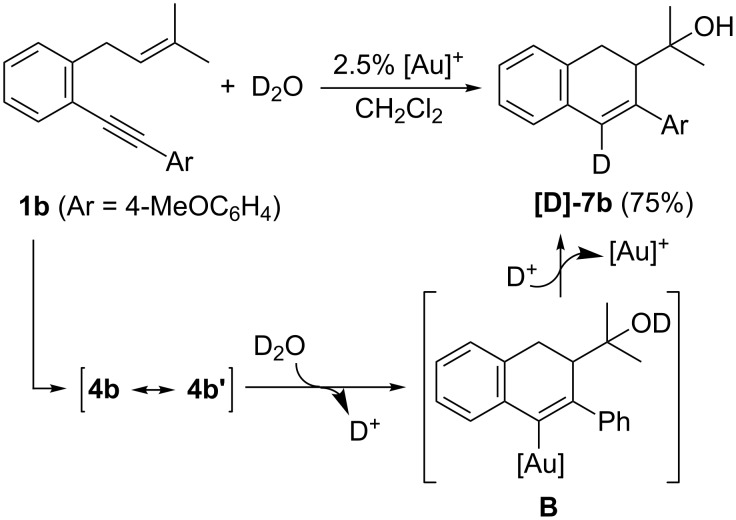
Labelling experiment and proposed mechanism.

Finally, to support the proposed intermediacy of gold–carbenoid intermediate **4** or **4’** (Scheme 4), we treated enyne **1b** with D_2_O instead of water and under the same catalytic conditions we observed the exclusive formation of the deuterated compound [D]-**7b** in 75% yield (>90% deuterium incorporation at C4). The generation of that compound could be explained by deuterodemetallation of the vinylgold species **B** generated by an attack of the nucleophile on intermediate **4b** or **4b’** (Scheme 8).

## Conclusion

We described an efficient gold(I)-catalyzed 6-*endo* hydroxycyclization of 7-substituted 1,6-enynes bearing a condensed aromatic ring at the C3–C4 position of the enyne. This type of cyclization has not been previously observed for 1,6-enynes bearing trisubstituted olefins and represents a new addition to the observed reaction topologies in the gold-catalyzed cycloisomerization of these substrates. The new oxygen-functionalized dihydronaphthalene derivatives have been synthesized in high yields.

## Supporting Information

Experimental procedures and spectroscopic data for all new compounds. Copies of ^1^H NMR and ^13^C NMR spectra for new compounds.

File 1 Experimental and analytical data.

File 2 NMR spectra.
