# Supplementary material for: Gold(I)-catalyzed 6-endo hydroxycyclization of 7-substituted-1,6-enynes
Source: Beilstein J Org Chem. 2013 Oct 29;9:2242–9. doi: 10.3762/bjoc.9.263 (PMC3817472; doi:10.3762/bjoc.9.263)
Supplement: File 1 — Experimental and analytical data. [file Beilstein_J_Org_Chem-09-2242-s001.pdf]

Supporting Information

for

Gold(I)-catalyzed 6-*endo* hydroxycyclization of 7-substituted-1,6-enynes

Ana M. Sanjuán, Alberto Martínez, Patricia García-García, Manuel A. Fernández-Rodríguez and Roberto Sanz*

Área de Química Orgánica, Departamento de Química, Facultad de Ciencias, Universidad de Burgos, Pza. Misael Bañuelos s/n, 09001 Burgos, Spain

Email: Roberto Sanz – rsd@ubu.es

* Corresponding author

Experimental and analytical data

General procedure 1 (GP1) for the synthesis of *o*-(alkynyl)-(3-methylbut-2-enyl)benzenes 1:

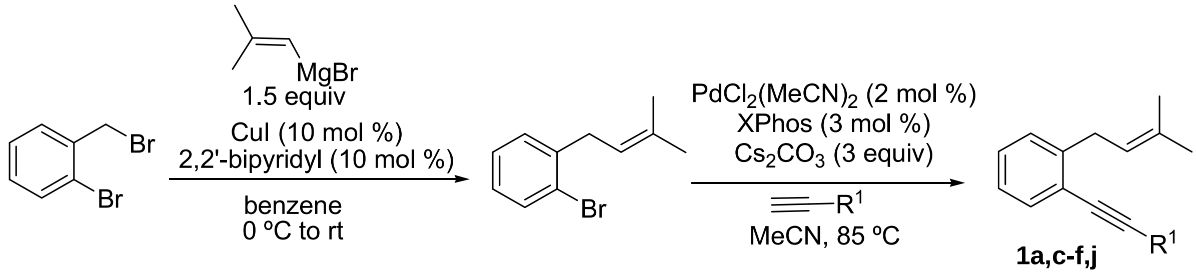

1a,c-f,j

Step 1: To a stirred mixture of 2-bromobenzylbromide (13 mmol, 3.25 g), CuI (10 mol%, 247 mg) and 2,2'-bipyridyl (10 mol %, 203 mg), in benzene (5 mL) at 0 ºC under nitrogen atmosphere was added 2-methyl-1-propenyl magnesium bromide (19.5 mmol, 0.5 M in THF, 39 mL). The cooling bath was removed and the reaction mixture was stirred at rt overnight. Saturated aq NH_4Cl (45 mL) and 25% aq NH_3 (25 mL) were added, and the mixture was stirred for 30 min and extracted with diethyl ether. The organic layer was washed with brine, dried over anhydrous Na_2SO_4, and concentrated under reduced pressure. The crude product was purified by column chromatography on silica gel using hexane as eluent to afford 1-bromo-2-(3-methylbut-2-en-1-yl)benzene in 90% yield.

Step 2: A mixture of 1-bromo-2-(3-methylbut-2-en-1-yl)benzene (6 mmol, 1.35 g), $PdCl_2(MeCN)_2$ (2 mol %, 31 mg), XPhos (3 mol %, 86 mg) and Cs_2CO_3 (18 mmol, 5.86 g), in anhydrous MeCN (12 mL) was stirred under a nitrogen atmosphere at rt for 25 min. Then the corresponding alkyne (9 mmol) was added and the mixture was stirred at 85 ºC until complete consumption of starting material, as monitored by TLC or GC−MS (2−3 h). Ethyl acetate and water were added to the reaction mixture and the aqueous phase was extracted with ethyl acetate. The combined organic layers were dried over anhydrous Na_2SO_4 and concentrated at reduced pressure. The remaining residue was purified by column chromatography on silica gel using mixtures of hexane and ethyl acetate as eluent to give the enynes **1a,c-f,j** (41−76%).

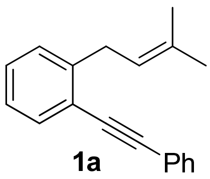

1a

1-(3-Methylbut-2-enyl)-2-(phenylethynyl)benzene (1a):

Yellow oil; yield = 71%; R_f = 0.25 (hexane); ^{1}H NMR (300 MHz, $CDCl_3$): δ 1.81 (s, 6H, 2 CH_3), 3.67 (d, *J* = 7.3 Hz, 2H, CH_2), 5.42−5.48 (m, 1H, =CH), 7.22−7.32 (m, 3H, ArH), 7.33−7.42 (m, 3H, ArH), 7.56−7.61 (m, 3H, ArH) ppm; ^{13}C NMR (75.4 MHz, $CDCl_3$): δ 18.1 (CH_3), 26.0 (CH_3), 33.2 (CH_2), 88.5 (C), 93.3 (C), 122.56 (CH), 122.62 (C), 123.7 (C), 125.9 (CH), 128.3 (CH), 128.5 (2 × CH), 128.57 (CH), 128.63 (CH), 131.6 (2 × CH), 132.3 (CH), 133.0 (C), 143.8 (C) ppm. LRMS (70 eV, EI): *m/z* (%) 246 (M^+, 34), 203 (100), 105 (69); HRMS (EI) calcd for $C_{19}H_{18}$: 246.1409, found: 246.1395.

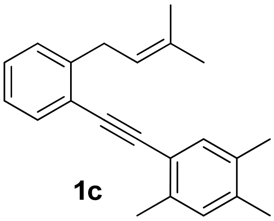

1c

1,2,4-Trimethyl-5-((2-(3-methylbut-2-en-1-yl)phenyl)ethynyl)benzene (1c):

Colourless oil; yield = 41%; R_f = 0.22 (hexane); ^{1}H NMR (300 MHz, $CDCl_3$): δ 1.83 (s, 3H, CH_3), 1.85 (s, 3H, CH_3), 2.30 (s, 3H, Ar-CH_3), 2.32 (s, 3H, Ar-CH_3), 2.55 (s, 3H, Ar-CH_3), 3.71 (d, *J* = 7.1 Hz, 2H, CH_2), 5.42-5.60 (m, 1H, =CH), 7.08 (s, 1H, ArH), 7.15−7.44 (m, 4H, ArH), 7.54−7.69 (m, 1H, ArH) ppm; ^{13}C NMR (75.4 MHz, $CDCl_3$): δ 18.1 (CH_3), 19.2 (CH_3), 19.8 (CH_3), 20.4 (CH_3), 25.9 (CH_3), 33.2 (CH_2), 91.3 (C), 92.8 (C), 120.6 (C), 122.6 (CH), 123.2 (C), 125.8 (CH), 128.3 (CH), 128.4 (CH), 131.0 (CH), 132.2 (CH), 132.92 (CH), 132.94 (C), 133.8 (C), 137.1 (C), 137.4 (C), 143.4 (C) ppm.

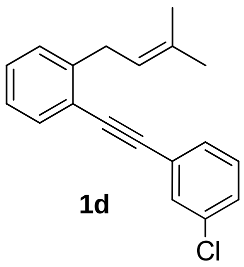

1d

1-((3-Chlorophenyl)ethynyl)-2-(3-methylbut-2-en-1-yl)benzene (1d):

Colourless oil; yield = 51%; R_f = 0.70 (hex/EtOAc 10:1); ^{1}H NMR (300 MHz, $CDCl_3$): δ 1.78 (s, 3H, CH_3), 1.79 (s, 3H, CH_3), 3.61 (d, *J* = 7.1 Hz, 2H, CH_2), 5.31−5.48 (m, 1H, =CH), 7.15−7.37 (m, 5H, ArH), 7.39−7.48 (m, 1H, ArH), 7.49−7.65 (m, 2H, ArH) ppm; ^{13}C NMR (75.4 MHz, $CDCl_3$): δ 18.1 (CH_3), 25.9 (CH_3), 33.2 (CH_2), 89.7 (C), 91.9 (C), 122.1 (C), 122.4 (CH), 125.4 (C), 125.9 (CH), 128.5 (CH), 128.7 (CH), 129.0 (CH), 129.7 (2 × CH), 131.4 (CH), 132.4 (CH), 133.1 (C), 134.3 (C), 144.0 (C) ppm; LRMS (70 eV, EI): *m/z* (%) 282 [$(M+2)^+$, 28], 280 (M^+, 94), 265 (78), 230 (83), 215 (100); HRMS (EI) calcd for $C_{19}H_{17}Cl$: 280.1019, found: 280.1016.

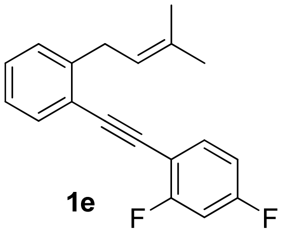

1e

2,4-Difluoro-1-((2-(3-methylbut-2-en-1-yl)phenyl)ethynyl)benzene (1e):

Yellow oil; yield = 63%; R_f = 0.38 (hexane); ^{1}H NMR (300 MHz, $CDCl_3$): δ 1.78 (s, 3H, CH_3), 1.79 (s, 3H, CH_3), 3.64 (d, *J* = 7.2 Hz, 2H, CH_2), 5.35-5.51 (m, 1H, =CH), 6.79-7.01 (m, 2H, ArH), 7.09−7.40 (m, 3H, ArH), 7.41−7.65 (m, 2H, ArH) ppm; ^{13}C NMR (75.4 MHz, $CDCl_3$): δ 18.0 (CH_3), 25.9 (CH_3), 33.1 (CH_2), 85.6 (C), 93.3 (d, *J* = 3.5 Hz, C), 104.4 (t, *J* = 25.4 Hz, CH), 108.7 (d, *J* = 12.1 Hz, C), 111.7 (dd, *J* = 21.9, 3.7 Hz, CH), 122.1 (C), 122.4 (CH), 125.9 (CH), 128.6 (CH), 129.0 (CH), 132.3 (CH), 133.1 (C), 134.13 (dd, *J* = 9.7, 2.6 Hz, CH), 144.0 (C), 162.7 (dd, *J* = 252.0, 11.1 Hz,

C), 163.0 (dd, J = 254.3, 12.1 Hz, C) ppm; LRMS (70 eV, EI): m/z (%) 282 (M^+, 51), 267 (100); HRMS (EI) calcd for $C_{19}H_{16}F_2$: 282.1220, found: 282.1206.

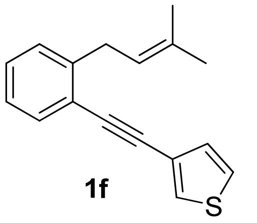

1f

3-((2-(3-Methylbut-2-en-1-yl)phenyl)ethynyl)thiophene (1f):

Yellow oil; yield = 55%; R_f = 0.28 (hexane); ^{1}H NMR (300 MHz, $CDCl_3$): δ 1.82 (s, 6H, 2 CH_3), 3.66 (d, J = 7.3 Hz, 2H, CH_2), 5.36−5.54 (m, 1H, =CH), 7.12−7.41 (m, 5H, ArH), 7.48−7.69 (m, 2H, ArH) ppm; ^{13}C NMR (75.4 MHz, $CDCl_3$): δ 18.1 (CH_3), 25.9 (CH_3), 33.2 (CH_2), 87.9 (C), 88.4 (C), 120.4 (C), 122.5 (CH), 122.6 (C), 125.4 (CH), 125.8 (CH), 128.4 (CH), 128.6 (2 × CH), 129.9 (CH), 132.2 (CH), 132.9 (C), 143.7 (C) ppm. LRMS (70 eV, EI): m/z (%) 252 (M^+, 100), 237 (82), 221 (51); HRMS (EI) calcd for $C_{17}H_{16}S$: 252.0973, found: 252.0984.

General procedure 2 (GP2) for the synthesis of *o*-(alkynyl)-(3-methylbut-2-enyl)benzenes 1:

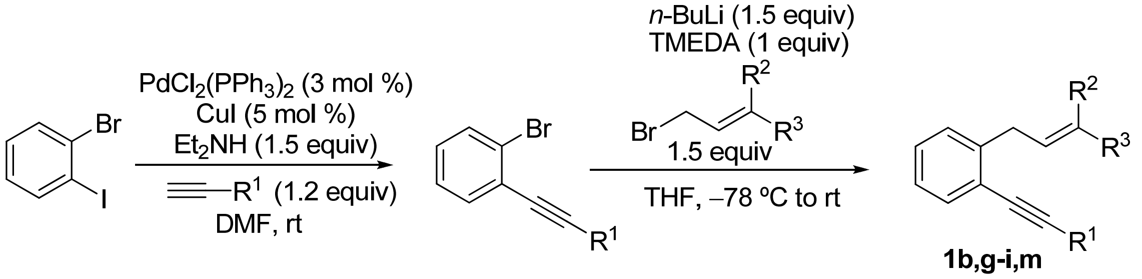

Step 1: A mixture of 1-bromo-2-iodobenzene (7 mmol, 1.98 g), the corresponding alkyne (8.4 mmol), $PdCl_2(PPh_3)_2$ (3 mol %, 148 mg), CuI (5 mol %, 66.5 mg) and Et_2NH (10.5 mmol, 1.25 mL), in anhydrous DMF (28 mL) was stirred under a nitrogen atmosphere at rt until complete consumption of starting material, as monitored by

TLC or GC-MS (3−6 h). CH_2Cl_2 and water were added to the reaction mixture and the aqueous phase was extracted with CH_2Cl_2. The combined organic layers were washed with water, dried over anhydrous Na_2SO_4 and concentrated at reduced pressure. The remaining residue was purified by column chromatography on silica gel using mixtures of hexane and ethyl acetate as eluent to afford the corresponding *o*-alkynylbromobenzenes (74−97%) which were used in the next step.

Step 2: A solution of the corresponding *o*-alkynylbromobenzene (6 mmol) in THF (8 mL) was treated with *n*-BuLi (9 mmol, 1.6 M in hexanes, 5.65 mL) at –78 ºC under a nitrogen atmosphere and the resulting mixture stirred for 20 min. Tetramethylethylenediamine (6 mmol, 0.91 mL) was then added and the mixture was stirred for additional 20 min before addition of the corresponding bromide (9 mmol). The resulting solution was allowed to react at rt overnight before it was quenched with water and extracted with diethyl ether. The organic layer was dried over anhydrous Na_2SO_4, concentrated under reduced pressure and purified by column chromatography on silica gel using mixtures of hexane and ethyl acetate as eluent to afford the corresponding enynes **1b,g-i,m** (68-92%).

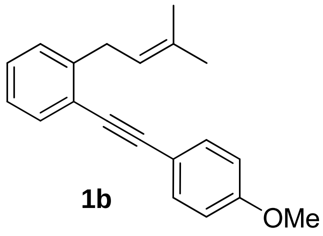

1b

1-((4-Methoxyphenyl)ethynyl)-2-(3-methylbut-2-en-1-yl)benzene (1b):

Colourless oil; yield = 86%; R_f = 0.36 (hex/AcOEt 10:1); ^{1}H NMR (400 MHz, $CDCl_3$): δ 1.77 (s, 6H, 2 CH_3), 3.61 (d, *J* = 7.3 Hz, 2H, CH_2), 3.83 (s, 3H, OCH_3), 5.32−5.48 (m, 1H, =CH), 6.89 (d, *J* = 8.9 Hz, 2H, ArH), 7.14-7.36 (m, 3H, ArH), 7.49 (d, *J* = 8.9 Hz, 2H, ArH), 7.47-7.57 (m, 1H, ArH) ppm; ^{13}C NMR (100.6 MHz, $CDCl_3$): δ 18.1

(CH_3), 25.9 (CH_3), 33.2 (CH_2), 55.4 (CH_3), 87.1 (C), 93.3 (C), 114.1 (2 × CH), 115.8 (C), 122.6 (CH), 122.9 (C), 125.8 (CH), 128.3 (CH), 128.5 (CH), 131.6 (C), 132.1 (CH), 133.0 (2 × CH), 143.6 (C), 159.6 (C) ppm. LRMS (70 eV, EI): *m/z* (%) 276 (M^+, 100), 208 (68), 161 (49); HRMS (EI) calcd for $C_{20}H_{20}O$: 276.1514, found: 276.1515.

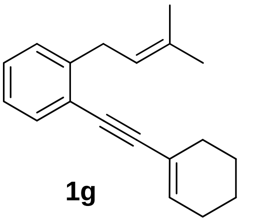

1g

1-((Cyclohex-1-en-yl)ethynyl)-2-(3-methylbut-2-enyl)benzene (1g):

Yellow oil; yield = 62%; R_f = 0.50 (hexane); ^{1}H NMR (400 MHz, $CDCl_3$): δ 1.53−1.90 (m, 4H, *c*-C_6H_9), 1.75 (s, 3H, CH_3), 1.76 (s, 3H, CH_3), 2.09−2.21 (m, 2H, *c*-C_6H_9), 2.22−2.36 (m, 2H, *c*-C_6H_9), 3.53 (d, *J* = 7.3 Hz, 2H, CH_2), 5.28−5.46 (m, 1H, =CH), 6.11−6.31 (m, 1H, =CH), 7.04−7.34 (m, 3H, ArH), 7.35−7.51 (m, 1H, ArH) ppm; ^{13}C NMR (100.6 MHz, $CDCl_3$): δ 18.1 (CH_3), 21.7 (CH_2), 22.5 (CH_2), 25.9 (CH_2), 25.9 (CH_3), 29.4 (CH_2), 33.1 (CH_2), 85.8 (C), 95.3 (C), 121.1 (C), 122.6 (CH), 123.1 (C), 125.7 (CH), 128.1 (CH), 128.4 (CH), 131.5 (C), 132.1 (CH), 134.8 (CH), 143.5 (C) ppm. LRMS (70 eV, EI): *m/z* (%) 250 (M^+, 50), 235 (100), 165 (95); HRMS (EI) calcd for $C_{19}H_{22}$: 250.1722, found: 250.1729.

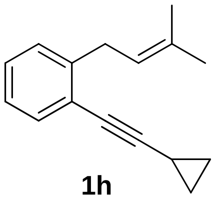

1h

1-(Cyclopropylethynyl)-2-(3-methylbut-2-enyl)benzene (1h):

Colourless oil; yield = 74%; R_f = 0.30 (hexane); ^{1}H NMR (400 MHz, $CDCl_3$): δ 0.72−1.01 (m, 4H, 2 CH_2, *c*-C_3H_5), 1.41−1.57 (m, 1H, CH, *c*-C_3H_5), 1.75 (s, 3H, CH_3),

1.77 (s, 3H, CH_3), 3.49 (d, *J* = 7.5 Hz, 2H, CH_2), 5.22−5.45 (m, 1H, =CH), 7.10 (td, *J* = 7.5 Hz, *J* = 2.0 Hz, 1H, ArH), 7.14−7.30 (m, 2H, ArH), 7.37 (dd, *J* = 7.6 Hz, *J* = 1.0 Hz, 1H, ArH) ppm; ^{13}C NMR (100.6 MHz, $CDCl_3$): δ 0.5 (CH), 8.9 (2 × CH_2), 18.0 (CH_3), 25.9 (CH_3), 33.1 (CH_2) 74.6 (C), 97.4 (C), 122.7 (CH), 123.2 (C), 125.6 (CH), 127.7 (CH), 128.3 (CH), 132.2 (CH), 132.6 (C), 143.7 (C) ppm. LRMS (70 eV, EI): *m/z* (%) 210 (M^+, 5), 195 (76), 167 (100); HRMS (EI) calcd for $C_{16}H_{18}$: 210.1409, found: 210.1408.

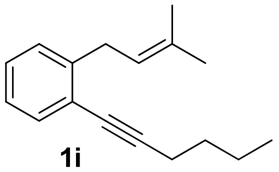

1i

1-(Hex-1-yn-1-yl)-2-(3-methylbut-2-enyl)benzene (1i):

Colourless oil; yield = 89%; R_f = 0.28 (hexane); ^{1}H NMR (300 MHz, $CDCl_3$): δ 0.97 (t, *J* = 7.3 Hz, 3H, $CH_3CH_2CH_2CH_2$), 1.46-1.66 (m, 4H, CH_3*CH_2CH_2*CH_2), 1.76 (s, 3H, CH_3), 1.77 (s, 3H, CH_3), 2.47 (t, *J* = 6.7 Hz, 2H, $CH_3CH_2CH_2$*CH_2*), 3.52 (d, *J* = 7.3 Hz, 2H, CH_2), 5.26−5.44 (m, 1H, =CH), 7.02−7.31 (m, 3H, ArH), 7.34−7.49 (m, 1H, ArH) ppm; ^{13}C NMR (75.4 MHz, $CDCl_3$): δ 13.8 (CH_3), 18.0 (CH_3), 19.4 (CH_2), 22.2 (CH_2), 25.9 (CH_3), 31.1 (CH_2), 33.1 (CH_2), 79.4 (C), 94.4 (C), 127.7 (CH), 123.4 (C), 125.7 (CH), 127.8 (CH), 128.3 (CH), 132.2 (CH), 132.7 (C), 143.5 (C) ppm. LRMS (70 eV, EI): *m/z* (%) 226 (M^+, 14), 155 (100), 141 (81); HRMS (EI) calcd for $C_{17}H_{22}$: 226.1722, found: 226.1729.

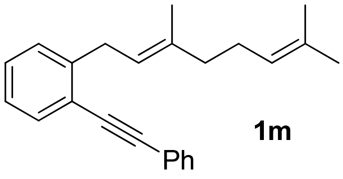

1m

(*E*)-1-(3,7-Dimethylocta-2,6-dien-1-yl)-2-(phenylethynyl)benzene (1m):

Colourless oil; yield = 58%; R_f = 0.21 (hexane); ^{1}H NMR (400 MHz, $CDCl_3$): δ 1.62 (s, 3H, CH_3), 1.70 (s, 3H, CH_3), 1.77 (s, 3H, CH_3), 1.96−2.34 (m, 4H, 2 CH_2), 3.65 (d, J = 7.2 Hz, 2H, CH_2), 5.03−5.24 (m, 1H, =CH), 5.35−5.55 (m, 1H, =CH), 7.20 (td, J = 7.5, 2.0 Hz, 1H, ArH), 7.24−7.46 (m, 5H, ArH), 7.47−7.69 (m, 3H, ArH) ppm; ^{13}C NMR (100.6 MHz, $CDCl_3$): δ 16.4 (CH_3), 17.8 (CH_3), 25.8 (CH_3), 26.8 (CH_2), 33.0 (CH_2), 39.9 (CH_2), 88.5 (C), 93.4 (C), 122.4 (CH), 122.7 (C), 123.7 (C), 124.4 (CH), 125.8 (CH), 128.3 (CH), 128.46 (2 × CH), 128.51 (CH), 128.6 (CH), 131.57 (C), 131.61 (2 × CH), 132.2 (CH), 136.7 (C), 143.8 (C) ppm. LRMS (70 eV, EI): *m/z* (%) 314 (M^+,10), 245 (100), 215 (69); HRMS (EI) calcd for $C_{24}H_{26}$: 314.2035, found: 314.2043.

Trimethyl((2-(3-methylbut-2-en-1-yl)phenyl)ethynyl)silane (1j) and 1-Ethynyl-2-(3-methylbut-2-enyl)benzene (1l):

Both substrates were prepared following a reported procedure [1].

Procedure for the synthesis of 1-ethynyl-2-(3-methylbut-2-enyl)benzene (1k):

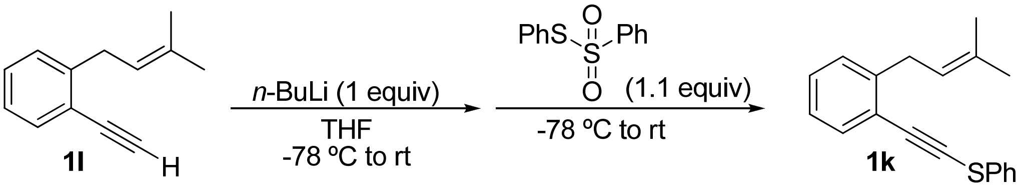

To a solution of the terminal enyne (**1l,** 0.76 mmol, 130 mg) in THF (2 mL) at –78 ºC, under nitrogen atmosphere, was added *n*-BuLi (0.76 mmol, 1.6 M in THF, 0.48 mL). The solution was stirred at rt for 30 min, the cooled to –78 ºC. S-phenyl benzenesulfonothioate (0.84 mmol, 210 mg) was added at –78 ºC and then the mixture was allowed to react at rt for 3 h before it was quenched with saturated aq NH_4Cl. The mixture was extracted with CH_2Cl_2 and the organic layer was dried over anhydrous Na_2SO_4 and concentrated at reduced pressure. The crude residue was chromatographed on a silica column using hexane as eluent to give enyne **1k** as a

yellow oil in 65% yield; R_f = 0.31 (hexane); ^{1}H NMR (300 MHz, $CDCl_3$): δ 1.79 (s, 3H, CH_3), 1.82 (s, 3H, CH_3), 3.64 (d, J = 7.2 Hz, 2H, CH_2), 5.32−5.49 (m, 1H, =CH), 7.16−7.46 (m, 6H, ArH), 7.49−7.66 (m, 3H, ArH) ppm; ^{13}C NMR (75.4 MHz, $CDCl_3$): δ 18.1 (CH_3), 25.9 (CH_3), 33.3 (CH_2), 78.9 (C), 96.9 (C), 122.3 (C), 122.4 (CH), 125.9 (CH), 126.2 (2 × CH), 126.5 (CH), 128.7 (CH), 129.0 (CH), 129.3 (2 × CH), 132.6 (CH), 133.1 (C), 133.3 (C), 144.2 (C) ppm. LRMS (70 eV, EI): *m/z* (%) 278 (M^+, 100), 169 (43), 128 (38).

Procedure for the synthesis of 2-(4-methylpent-3-en-2-yl)-1-(2-phenylethynyl)benzene (1n):

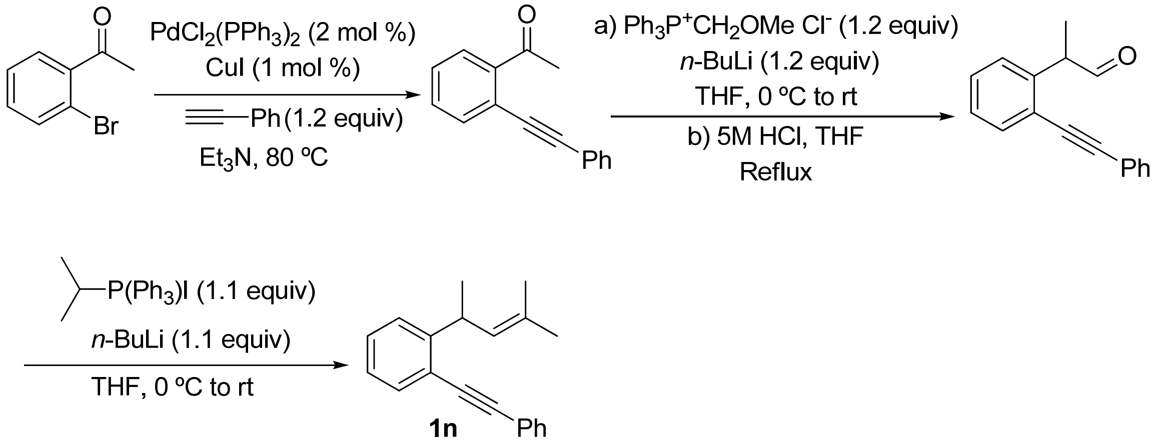

Step 1: To a solution of 2-bromoacetophenone (20 mmol, 3.98 g) in Et_3N (60 mL), under nitrogen atmosphere, phenylacetylene (24 mmol, 2.45 g) and $PdCl_2(PPh_3)_2$ (2 mol%, 280 mg) were added. The reaction was stirred at rt for 15 min, then CuI (1 mol%, 40 mg) was added. The reaction mixture was stirred at 80 ºC until complete consumption of starting material was detected by TLC analysis (2 h). Then, the solvent was evaporated under reduced pressure and the crude purified by flash chromatography over a silica gel column using a 10:1 mixture of hexane and EtOAc as eluent to afford the corresponding *o*-alkynylacetophenone in 95% yield.

Step 2: To a solution of (methoxymethyl)triphenylphosphonium chloride (14.4 mmol, 4.94 g) in THF (27 mL) at 0 ºC, under nitrogen atmosphere, was added *n*-BuLi (14.4 mmol, 1.6 M in hexanes, 9 mL). The mixture was stirred at 0 ºC for 30 min. Then a solution of *o*-alkynylacetophenone (12 mmol, 2.46 g) in THF (5 mL) was added. The reaction was allowed to stir at rt until TLC analysis revealed consumption of the ketone (2 h). The mixture was quenched with saturated NH_4Cl and diluted with diethyl ether. The mixture was extracted with diethyl ether, and the combined organic layers were washed with brine, dried over anhydrous Na_2SO_4, and concentrated in vacuo. The residue was purified by column chromatography on silica gel using a mixture of hexane and EtOAc 10:1 as eluent. The enol ether was dissolved in THF (20 mL). HCl (4M, 10 mL) was added to the reaction mixture, which was then refluxed until TLC analysis revealed complete hydrolysis of the enol ether (5 h). After cooling, the reaction mixture was diluted with diethyl ether and neutralized with saturated $NaHCO_3$. The mixture was then extracted with diethyl ether. The combined organic layers were washed with brine, dried over Na_2SO_4, and concentrated under reduced pressure. The crude product was purified by column chromatography on silica gel using a mixture of hexane and ethyl acetate 10:1 as eluent to afford 2-(2-(phenylethynyl)phenyl)propanal in 54% yield.

Step 3: To a solution of isopropyltriphenyl phosphonium iodide (5.9 mmol, 2.57 g) in THF (12 mL) at 0 ºC, under nitrogen atmosphere, was added *n*-BuLi (5.9 mmol, 1.6 M in hexanes, 3.7 mL). The mixture was stirred for 30 min at rt, then cooled to 0 ºC. A solution of 2-(2-(phenylethynyl)phenyl)propanal (5.4 mmol, 1.25 g) in THF (10 mL) was added and the reaction stirred at rt until TLC analysis revealed consumption of the aldehyde (3 h). CH_2Cl_2 and water were added to the reaction mixture. The separated aqueous phase was extracted with CH_2Cl_2. The combined organic layers were dried over anhydrous Na_2SO_4, and concentrated under reduced pressure. The

crude product was purified by column chromatography on silica gel using hexane as eluent to give the enyne **1n** as a colourless oil in 46% yield. R_f = 0.66 (hexane); ^{1}H NMR (300 MHz, $CDCl_3$): δ 1.40 (d, *J* = 7.0 Hz, 3H, CH_3), 1.75 (d, *J* = 1.3 Hz, 3H, CH_3), 1.77 (d, *J* = 1.3 Hz, 3H, CH_3), 4.38 (dq, *J* = 9.0 Hz, *J* = 7.0 Hz, 1H, CH), 5.44 (dsept, *J* = 9.0 Hz, *J* = 1.4 Hz, 1H, =CH), 7.16−7.24 (m, 1H, ArH), 7.28−7.49 (m, 5H, ArH), 7.51−7.70 (m, 3H, ArH) ppm; ^{13}C NMR (75.4 MHz, $CDCl_3$): δ 18.4 (CH_3), 22.9 (CH_3), 25.9 (CH_3), 36.3 (CH), 88.6 (C), 93.0 (C), 121.8 (C), 123.8 (C), 125.6 (CH), 126.4 (CH), 128.2 (CH), 128.5 (2 × CH), 128.9 (CH), 129.7 (CH), 131.5 (2 × CH), 131.6 (C), 132.5 (CH), 149.7 (C) ppm. LRMS (70 eV, EI): *m/z* (%) 260 (M^+, 100), 245 (61), 215 (79); HRMS (EI) calcd for $C_{20}H_{20}$: 260.1565, found: 260.1564.

.

Gold-catalyzed cycloisomerization of 1,6-enyne 1a:

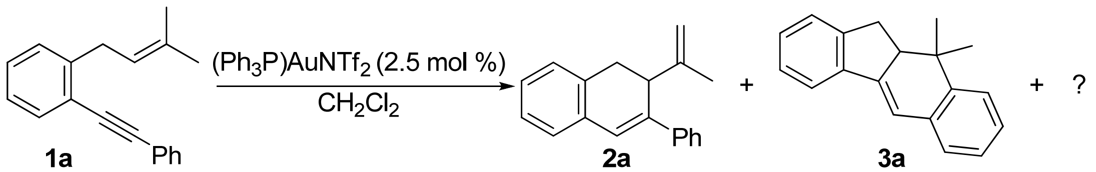

1a **2a** **3a**

(Ph_3P)$AuNTf_2$ (2.5 mol %, 5.5 mg) was added to a solution of 1,6-enyne **1a** (0.3 mmol, 74 mg) in DCM (0.6 mL) and the resulting mixture was stirred overnight (complete consumption of starting material was confirmed by TLC). The solution was diluted with hex/EtOAc: 9/1 and filtered through a pad of celite. The solvent was removed and the crude residue was purified by column chromatography on silica gel using hexane as eluent giving rise to a ca. 3/1 inseparable mixture of 3-phenyl-2-(prop-1-en-2-yl)-1,2-dihydronaphthalene (**2a)** and tetracyclic compound 10,10-dimethyl-10a,11-dihydro-10*H*-benzo[*b*]fluorene (**3a)** in 68% overall yield. R_f = 0.58 (hex/EtAcOEt 10:1); ^{1}H NMR (300 MHz, $CDCl_3$): δ 1.09 (s, 3H, CH_3, **3a**), 1.64 (s, 3H, CH_3, **3a**), 1.87 (s, 3H, CH_3, **2a**), 3.09 (dd, *J* = 15.7, 2.1 Hz, 1H, CH, **2a**), 3.10−3.29 (m, 3H, CH_2+CH, **3a**), 3.36 (dd, *J* = 15.7, 7.6 Hz, 1H, C*H*H, **2a**), 3.56 (d, *J* = 6.9 Hz,

1H, CH*H*, **2a**), 4.84 (s, 1H, =C*H*H, **2a**), 4.89 (s, 1H, =CH*H*, **2a**), 6.99 (s, 1H, =CH, **3a**), 7.13 (s, 1H, =CH **2a**), 7.19−7.79 (m, 17H, ArH, **2a** + **3a**) ppm; ^{13}C NMR (75.4 MHz, $CDCl_3$): δ 21.3 (CH_3, **2a**), 22.0 (CH_3, **3a**), 25.7 (CH_3, **3a**), 31.5 (CH_2, **3a**), 33.8 (CH_2, **2a**), 37.2 (C, **3a**), 43.8 (CH, **2a**), 48.5 (CH, **3a**), 112.4 (CH_2, **2a**), 115.7 (CH, **3a**), 121.0 (CH, **3a**), 123.8 (CH, **3a**), 125.4 (CH, **2a**), 125.6 (CH, **3a**), 125.7 (2 × CH , **2a**), 126.4 (CH, **3a**), 126.5 (CH, **2a**), 126.7 (CH, **2a**), 126.8 (CH, **3a**), 127.1 (CH, **3a**), 127.2 (CH, **3a**), 127.38 (CH, **2a**), 127.40 (CH, **2a**), 127.7 (CH, **2a**), 128.48 (2 × CH, **2a**), 128.51 (CH, **3a**), 133.6 (C, **2a**), 134.5 (C, **2a**), 139.90 (C, **2a**), 139.93 (C, **3a**), 140.7 (C, **2a**), 144.0 (C, **2a** + **3a**), 144.6 (C, **3a**), 145.4 (C, **3a**), 146.7 (C, **3a**) ppm.

General procedure for the gold-catalyzed synthesis of 2-(1,2-dihydronaphthalen-2-yl)propan-2-ol derivatives 7:

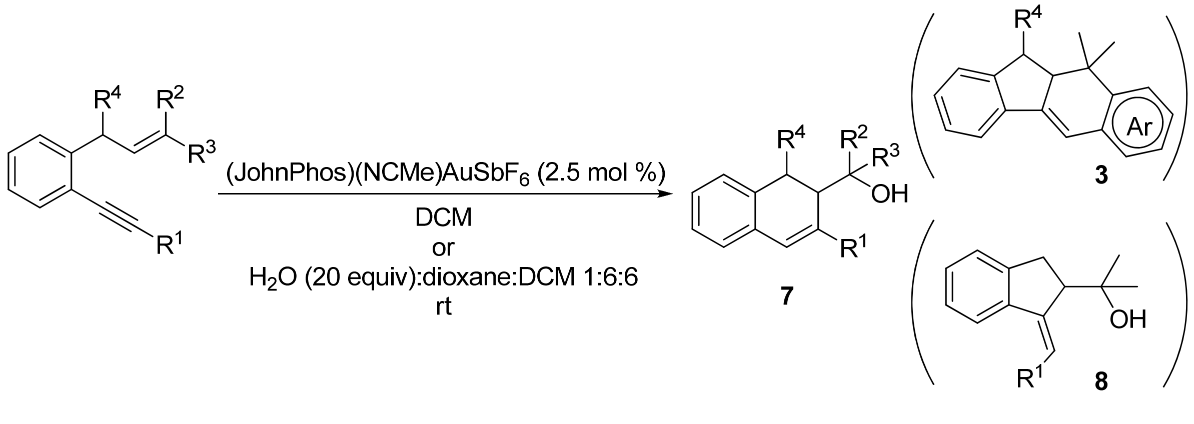

Water (20 equiv, 0.108 mL) was added to a solution of (JohnPhos)(NCMe)$AuSbF_6$ (2.5 mol %, 6 mg) in DCM (0.6 mL) (or in a mixture of DCM (0.33 mL)) and dioxane (0.65 mL), see Tables 1 and 2 and Scheme 6. After stirring at rt for 5−10 min a solution of the corresponding enyne **1** (0.3 mmol) in DCM (0.6 mL (or 0.32 mL for reactions performed in DCM/dioxane)) was added and the resulting mixture was stirred overnight (complete consumption of starting material was confirmed by TLC). The solution was diluted with hex/EtOAc: 9/1 and filtered through a pad of celite. The solvent was removed and the crude residue was purified by column chromatography

on silica gel using mixtures of hexane and EtOAc as eluents to afford the corresponding dihydronaphthalene derivatives **7** in the yields reported in Table 2 and Scheme 6. In reactions where minor amounts of **3** and/or **8** were formed they were isolated and characterized.

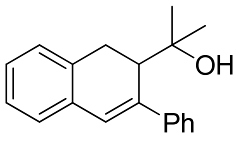

7a

2-(3-Phenyl-1,2-dihydronaphthalen-2-yl)propan-2-ol (7a):

White solid; yield = 77%; R_f = 0.19 (hex/AcOEt 5:1); m.p. = 114−116 ºC; ^{1}H NMR (300 MHz, $CDCl_3$): δ 0.93 (s, 3H, CH_3), 1.03 (s, 3H, CH_3), 1.28 (s, 1H, OH), 3.14 (dd, *J* = 6.0, 3.2 Hz, 1H, CH), 3.19−3.41 (m, 2H, CH_2), 6.88 (s, 1H, =CH), 7.08−7.33 (m, 5H, ArH), 7.35−7.50 (m, 2H, ArH), 7.50−7.71 (m, 2H, ArH) ppm; ^{13}C NMR (75.4 MHz, $CDCl_3$): δ 27.6 (CH_3), 29.0 (CH_3), 30.9 (CH_2), 45.5 (CH), 75.2 (C), 126.3 (CH), 126.5 (CH), 126.7 (2 × CH), 127.1 (CH), 127.4 (CH), 127.7 (CH), 128.0 (CH), 128.8 (2 × CH), 134.5 (C), 135.2 (C), 140.1 (C), 143.1 (C) ppm; LRMS (70 eV, EI): *m/z* (%) 268 (M^+), 206 (100), 205 (24), 91 (11), 59 (26); HRMS (EI) calcd for $C_{19}H_{20}O$: 264.1514, found: 264.1510.

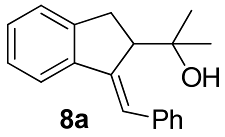

8a

(*E*)-2-(1-Benzylidene-2,3-dihydro-1*H*-inden-2-yl)propan-2-ol (8a):

Yellow oil; R_f = 0.24 (hex/AcOEt 5:1); ^{1}H NMR (300 MHz, $CDCl_3$): δ 0.84 (s, 3H, CH_3), 1.11 (s, 3H, CH_3), 1.56 (s, 1H, OH), 2.94 (d, *J* = 16.6 Hz, 1H, C*H*H), 3.19 (dd, *J* = 16.6, 7.7 Hz, 1H, CH*H*), 3.88 (d, *J* = 7.7 Hz, 1H, CH) 7.14 (s, 1H, =CH), 7.17−7.32 (m, 4H, ArH), 7.32–7.42 (m, 2H, ArH), 7.51–7.70 (m, 3H, ArH) ppm; ^{13}C NMR (100.6

MHz, $CDCl_3$): δ 26.8 (CH_3), 27.8 (CH_3), 34.8 (CH_2), 51.4 (CH), 75.3 (C), 119.8 (CH), 123.8 (CH), 124.9 (CH), 126.9 (CH), 127.1 (CH), 128.4 (CH), 128.7 (2 × CH), 129.1 (2 × CH), 138.1 (C), 143.4 (C), 144.4 (C), 144.7 (C) ppm.

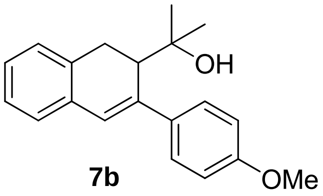

7b

2-(3-(4-Methoxyphenyl)-1,2-dihydronaphthalen-2-yl)propan-2-ol (7b):

White solid; yield = 80%; R_f = 0.08 (hex/AcOEt 9:1); m.p. = 108−110 ºC; ^{1}H NMR (400 MHz, $CDCl_3$): δ 0.90 (s, 3H, CH_3), 1.02 (s, 3H, CH_3), 1.28 (s, 1H, OH), 3.08 (dd, J = 6.3, 3.0 Hz, 1H, CH), 3.16−3.30 (m, 2H, CH_2), 3.82 (s, 3H, OCH_3), 6.80 (s, 1H, =CH), 6.92 (d, J = 8.8 Hz, 2H, ArH), 7.03−7.21 (m, 4H, ArH), 7.48 (d, J = 8.8 Hz, 2H, ArH) ppm; ^{13}C NMR (100.6 MHz, $CDCl_3$): δ 27.7 (CH_3), 28.9 (CH_3), 31.0 (CH_2), 45.6 (CH), 55.4 (CH_3), 75.1 (C), 114.1 (2 × CH), 126.1 (CH), 126.5 (CH), 126.6 (CH), 127.1 (CH), 127.4 (CH), 127.9 (2 × CH), 134.8 (C), 135.0 (C), 135.5 (C), 139.6 (C), 159.1 (C) ppm; LRMS (70 eV, EI): m/z (%) 294 (M^+, 4), 236 (100), 59 (19); HRMS (EI) calcd for $C_{20}H_{22}O_2$: 294.1620, found: 294.1620.

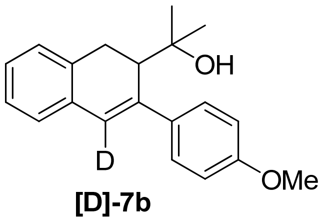

[D]-7b

2-(3-(4-Methoxyphenyl)-4-deutero-1,2-dihydronaphthalen-2-yl)propan-2-ol ([D]-7b):

White solid; yield = 75%. This compound was prepared following the general procedure using D_2O instead of water. R_f = 0.13 (hex/AcOEt 5:1); m.p. = 102−104 ºC; ^{1}H NMR (400 MHz, $CDCl_3$): δ 0.92 (s, 3H, CH_3), 1.03 (s, 3H, CH_3), 1.39 (s, 1H,

OH), 3.10 (dd, J = 6.3 Hz, J = 3.0 Hz, 1H, CH), 3.18−3.33 (m, 2H, CH_2), 3.83 (s, 3H, OCH_3), 6.94 (d, J = 8.9 Hz, 2H, ArH), 7.05−7.23 (m, 4H, ArH), 7.50 (d, J = 8.9 Hz, 2H, ArH) ppm; ^{13}C NMR (100.6 MHz, $CDCl_3$): δ 27.6 (CH_3), 28.9 (CH_3), 30.9 (CH_2), 45.5 (CH), 55.3 (CH_3), 75.1 (C), 114.1 (2 × CH), 126.0 (CH), 126.4 (CH), 127.0 (CH), 127.3 (CH), 127.8 (2 × CH), 134.7 (C), 135.0 (C), 135.4 (C), 139.4 (C), 159.1 (C) ppm; the signal corresponding to C−D was not observed. HRMS (EI) calcd for $C_{17}H_{14}DO$ [M^+−$C(CH_3)_2OH$]: 237.2264, found: 237.1271.

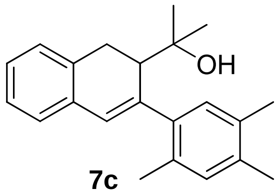

7c

2-(3-(2,4,5-Trimethylphenyl)-1,2-dihydronaphthalen-2-yl)propan-2-ol (7c):

White solid; yield = 71%; R_f = 0.21 (hex/AcOEt 5:1); m.p. = 51−53 ºC; ^{1}H NMR (300 MHz, $CDCl_3$): δ 0.95 (s, 3H, CH_3), 1.10 (s, 3H, CH_3), 1.42 (s, 1H, OH), 2.26 (s, 3H, Ar-CH_3), 2.27 (s, 3H, Ar-CH_3), 2.42 (s, 3H, Ar-CH_3), 2.87 (d, J = 7.9 Hz, 1H, CH), 3.20 (d, J = 16.5 Hz, 1H, C*H*H), 3.34 (dd, J = 16.5, 7.9 Hz, 1H, CH*H*), 6.56 (s, 1H, =CH), 7.03 (s, 1H, ArH), 7.07 (s, 1H, ArH), 7.04−7.13 (m, 1H, ArH), 7.14−7.23 (m, 3H, ArH), ppm; ^{13}C NMR (75.4 MHz, $CDCl_3$): δ 19.42 (CH_3), 19.44 (CH_3), 20.1 (CH_3), 27.8 (CH_3), 28.4 (CH_3), 30.9 (CH_2), 48.3 (CH), 75.3 (C), 126.0 (CH), 126.5 (CH), 127.2 (CH), 127.5 (CH), 129.8 (CH), 130.0 (CH), 132.0 (C), 132.4 (CH), 134.1 (C), 134.5 (C), 134.6 (C), 135.5 (C), 140.7 (C), 141.4 (C) ppm; LRMS (70 eV, EI): *m/z* (%) 306 (M^+, <5), 288 (28), 248 (100), 233 (56); HRMS (EI) calcd for $C_{19}H_{19}$ [M^+-$C(CH_3)_2OH$]: 248.1565, found: 248.1562.

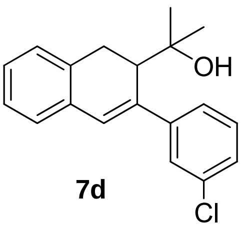

7d

2-(3-(3-Chlorophenyl)-1,2-dihydronaphthalen-2-yl)propran-2-ol (7d):

Colourless oil; yield = 63%; R_f = 0.20 (hex/AcOEt 5:1); ^{1}H NMR (300 MHz, $CDCl_3$): δ 0.92 (s, 3H, CH_3), 1.01 (s, 3H, CH_3), 1.25 (s, 1H, OH), 3.05 (dd, *J* = 6.8, 2.5 Hz, 1H, CH), 3.13−3.41 (m, 2H, CH_2), 6.86 (s, 1H, =CH), 7.01−7.37 (m, 6H, ArH), 7.37−7.49 (m, 1H, ArH), 7.49−7.69 (m, 1H, ArH) ppm; ^{13}C NMR (75.4 MHz, $CDCl_3$): δ 27.6 (CH_3), 29.1 (CH_3), 30.7 (CH_2), 45.4 (CH), 75.1 (C), 124.9 (CH), 126.58 (CH), 126.61 (CH), 126.7 (CH), 127.2 (CH), 127.3 (CH), 128.1 (CH), 129.0 (CH), 129.9 (CH), 134.1 (C), 134.6 (C), 135.2 (C), 138.8 (C), 145.2 (C) ppm; LRMS (70 eV, EI): *m/z* (%) 298 (M^+, <5), 280 (8), 240 (100), 202 (42); HRMS (EI) calcd for $C_{19}H_{19}ClO$: 298.1124, found: 298.1123.

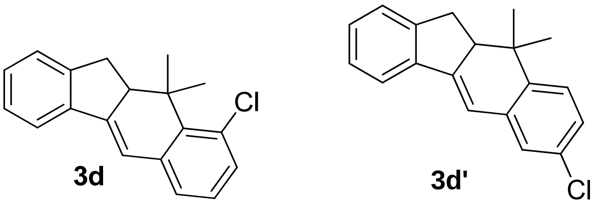

3d **3d'**

9-Chloro-10,10-dimethyl-10a,11-dihydro-10*H*-benzo[*b*]fluorene (3d):

7-Chloro-10,10-dimethyl-10a,11-dihydro-10*H*-benzo[*b*]fluorene (3d'):

Colourless oil; yield = 22%; R_f = 0.66 (hex/AcOEt 10:1); isolated as a 1:1 mixture of isomers; ^{1}H NMR (300 MHz, $CDCl_3$): δ 0.94 (s, 3H, CH_3), 1.12 (s, 3H, CH_3), 1.51 (s, 3H, CH_3), 1.88 (s, 3H, CH_3), 2.89-2.22 (m, 5H, CH + 2 x CH_2), 3.22−3.33 (m, 1H, CH), 6.76 (d, *J* = 3.1 Hz, 1H, =CH), 6.79 (d, *J* = 2.6 Hz, 1H, =CH), 7.02−7.45 (m, 12H, ArH), 7.49−7.65 (m, 2H, ArH) ppm; ^{13}C NMR (75.4 MHz, $CDCl_3$): δ 18.7 (CH_3), 21.9 (CH_3), 25.7 (CH_3), 27.4 (CH_3), 31.5 (CH_2), 32.3 (CH_2), 37.1 (C), 40.1 (C), 48.5

(CH), 49.9 (CH), 114.6 (CH), 115.7 (CH), 121.1 (CH), 121.2 (CH), 125.3 (CH), 125.6 (CH), 125.7 (CH), 126.57 (CH), 126.60 (CH), 126.7 (CH), 126.9 (CH), 127.0 (CH), 127.4 (CH), 127.9 (C), 128.9 (CH), 129.0 (CH), 129.8 (C), 130.6 (CH), 132.0 (C), 133.5 (C), 136.4 (C), 137.9 (C), 139.5 (C), 143.0 (C), 144.8 (C), 146.5 (C), 147.0 (C), 147.1 (C) ppm.

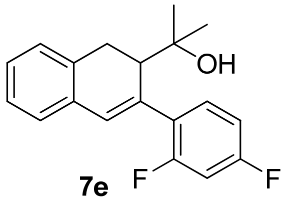

7e

2-(3-(2,4-Difluorophenyl)-1,2-dihydronaphthalen-2-yl)propan-2-ol (7e): Colourless oil; yield = 75%; R_f = 0.18 (hex/AcOEt 5:1); Isolated along with ≈10% of **8e**; ^{1}H NMR (300 MHz, $CDCl_3$): δ 0.93 (s, 3H, CH_3), 1.02 (s, 3H, CH_3), 1.28 (s, 1H, OH), 2.98 (dd, *J* = 7.1 Hz, *J* = 1.6 Hz, 1H, CH), 3.17−3.37 (m, 2H, CH_2), 6.74 (s, 1H, =CH), 6.77−6.99 (m, 2H, ArH), 7.01−7.29 (m, 4H, ArH), 7.30−7.45 (m, 1H, ArH) ppm; ^{13}C NMR (75.4 MHz, $CDCl_3$): δ 27.5 (CH_3), 28.8 (CH_3), 30.5 (CH_2), 46.8 (d, *J* = 3.1 Hz, CH), 75.0 (C), 104.4 (dd, *J* = 26.8, 25.2 Hz, CH), 111.5 (dd, *J* = 20.9, 3.6 Hz, CH), 126.5 (2 × CH), 127.2 (CH), 128.1 (CH), 130.7 (dd, *J* = 9.3, 5.3 Hz, CH), 131.1 (d, *J* = 1.6 Hz, CH), 133.9 (C), 134.3 (d, *J* = 1.7 Hz, C), 135.0 (C), 159.7 (dd, *J* = 249.7, 12.0 Hz, C), 162.0 (dd, *J* = 248.1, 11.2 Hz, C) ppm; the signal corresponding to a quaternary carbon was not observed; LRMS (70 eV, EI): *m/z* (%) 300 (M^+, <5), 242 (100), 220 (24); HRMS (EI) calcd for $C_{19}H_{18}F_2O$: 300.1326, found: 300.1319.

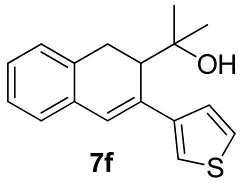

7f

2-(3-(Thiophen-3-yl)-1,2-dihydronaphthalen-2-yl)propan-2-ol (7f):

White solid; yield = 82%; R_f = 0.21 (hex/AcOEt 4:1); m.p. = 105−107 ºC; ^{1}H NMR (300 MHz, $CDCl_3$): δ 0.96 (s, 3H, CH_3), 1.12 (s, 3H, CH_3), 1.47 (s, 1H, OH), 3.05 (dd, J = 6.9, 2.3 Hz, 1H, CH), 3.09−3.37 (m, 2H, CH_2), 6.98 (s, 1H, =CH), 7.06−7.26 (m, 4H, ArH), 7.34 (dd, J = 4.9, 3.0 Hz, 1H, ArH), 7.39 (dd, J = 3.0, 1.6 Hz, 1H, ArH), 7.41 (dd, J = 4.9, 1.6 Hz, 1H, ArH) ppm; ^{13}C NMR (75.4 MHz, $CDCl_3$): δ 28.0 (CH_3), 28.5 (CH_3), 30.9 (CH_2), 46.2 (CH), 75.0 (C), 121.3 (CH), 126.0 (CH), 126.1 (CH), 126.2 (CH), 126.3 (CH), 126.5 (CH), 127.1 (CH), 127.6 (CH), 134.3 (C), 134.5 (C), 135.1 (C), 143.9 (C) ppm; LRMS (70 eV, EI): m/z (%) 270 (M^+, 2), 212 (100), 211 (36), 59 (27); HRMS (EI) calcd for $C_{17}H_{18}OS$: 270.1078, found: 270.1081.

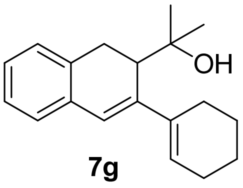

7g

2-(3-Cyclohexenyl-1,2-dihydronaphthalen-2-yl)propan-2-ol (7g):

Yellow oil; yield = 79%; R_f = 0.19 (hex/AcOEt 9:1); ^{1}H NMR (400 MHz, $CDCl_3$): δ 0.91 (s, 3H, CH_3), 1.13 (s, 3H, CH_3), 1.50−1.87 (m, 4H, c-C_6H_9), 1.73 (s, 1H, OH), 2.12−2.31 (m, 3H, c-C_6H_9), 2.40−2.61 (m, 1H, c-C_6H_9), 2.91 (dd, J = 6.7 Hz, J = 2.3 Hz, 1H, CH), 2.96−3.21 (m, 2H, CH_2), 6.12 (s, 1H, =CH), 6.64 (s, 1H, =CH), 6.85−7.23 (m, 4H, ArH) ppm; ^{13}C NMR (100.6 MHz, $CDCl_3$): δ 22.3 (CH_2), 23.2 (CH_2), 26.2 (CH_2), 27.0 (CH_2), 27.6 (CH_3), 28.3 (CH_3), 31.1 (CH_2), 42.7 (CH), 75.0 (C), 123.6 (CH), 126.3 (CH), 126.4 (CH), 126.6 (CH), 126.9 (CH), 127.1 (CH), 134.9 (C), 135.2 (C), 138.4 (C), 140.4 (C) ppm; LRMS (70 eV, EI): m/z (%) 268 (M^+, 4), 210 (100), 141 (46); HRMS (EI) calcd for $C_{19}H_{24}O$: 268.1827, found: 268.1830.

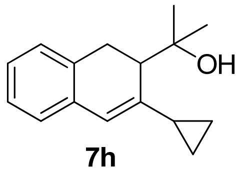

7h

2-(3-Cyclopropyl-1,2-dihydronaphthalen-2-yl)propan-2-ol (7h):

Yellow oil; yield = 77%; R_f = 0.14 (hex/AcOEt 9:1); ^{1}H NMR (400 MHz, $CDCl_3$): δ 0.58−0.79 (m, 2H, CH_2, *c*-C_3H_5), 0.81−0.95 (m, 2H, CH_2, *c*-C_3H_5), 1.05 (s, 3H, CH_3), 1.18 (s, 3H, CH_3), 1.47−1.62 (1H, m, CH, *c*-C_3H_5), 1.67 (s, 1H, OH), 2.47 (dd, *J* = 7.8, 1.5 Hz, 1H, CH), 2.98 (dd, *J* = 16.5, 1.5 Hz, 1H, C*H*H), 3.08 (dd, *J* = 16.5, 7.8 Hz, 1H, CH*H*), 6.09 (s, 1H, =CH), 6.82−6.87 (m, 1H, ArH), 6.97−7.20 (m, 3H, ArH) ppm; ^{13}C NMR (100.6 MHz, $CDCl_3$): δ 9.3 (CH_2), 9.5 (CH_2), 18.2 (CH), 28.0 (CH_3), 28.2 (CH_3), 31.2 (CH_2), 49.6 (CH), 74.8 (C), 120.1 (CH), 125.3 (CH), 126.4 (CH), 126.6 (CH), 127.0 (CH), 134.57 (C), 134.64 (C), 143.9 (C) ppm; LRMS (70 eV, EI): *m/z* (%) 228 (M^+, 4), 170 (100), 59 (65); HRMS (EI) calcd for $C_{16}H_{20}O$: 228.1514, found: 228.1522.

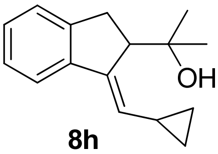

8h

(*E*)-2-(1-(Cyclopropylmethylene)-2,3-dihydro-1*H*-inden-2-yl)propan-2-ol (8h):

Colourless oil; yield = 21%; R_f = 0.20 (hex/AcOEt 9:1); ^{1}H NMR (300 MHz, $CDCl_3$): δ 0.44−0.58 (m, 2H, CH_2, *c*-C_3H_5), 0.81−0.98 (m, 2H, CH_2, *c*-C_3H_5), 0.92, (s, 3H, CH_3), 1.24 (s, 3H, CH_3), 1.77−1.91 (m, 1H, CH, *c*-C_3H_5), 2.02 (s, 1H, OH), 2.80 (d, *J* = 16.6 Hz, 1H, C*H*H), 3.12 (dd, *J* = 16.6, 8.2 Hz, 1H, CH*H*), 3.31 (d, *J* = 8.2 Hz, 1H, CH), 5.46 (dd, *J* = 10.3, 1.5 Hz, 1H =CH), 6.99−7.22 (m, 3H, ArH), 7.26−7.36 (m, 1H, ArH) ppm; ^{13}C NMR (75.4 MHz, $CDCl_3$): δ 7.4 (CH_2), 8.9 (CH_2), 13.0 (CH), 26.1 (CH_3), 27.5 (CH_3), 34.6 (CH_2), 52.0 (CH), 74.9 (C), 119.0 (CH), 124.8 (CH), 126.6 (CH),

127.3 (CH), 129.4 (CH), 140.8 (C), 142.7(C), 143.8 (C) ppm; LRMS (70 eV, EI): *m/z* (%) 228 (M^+, 13), 155 (100).

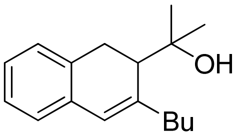

7i

2-(3-Butyl-1,2-dihydronaphthalen-2-yl)propan-2-ol (7i):

Yellow oil; yield = 82%; R_f = 0.15 (hex/AcOEt 12:1); ^{1}H NMR (400 MHz, $CDCl_3$): δ 0.95 (t, *J* = 7.3 Hz, 3H, $\mathit{CH_3}CH_2CH_2CH_2$), 1.04 (s, 3H, CH_3), 1.14 (s, 3H, CH_3), 1.31 (s, 1H, OH), 1.27−1.70 (m, 4H, $CH_3\mathit{CH_2CH_2}CH_2$), 2.28 (dd, *J* = 7.1, 2.0 Hz, 1H, CH), 2.31−2.48 (m, 2H, $CH_3CH_2CH_2\mathit{CH_2}$), 2.9 (dd, *J* = 16.4, 2.0 Hz, 1H, C*H*H), 3.05 (dd, *J* = 16.4, 7.1 Hz, 1H, CH*H*), 6.39 (s, 1H, =CH), 6.87−7.01 (m, 1H, ArH), 7.02−7.25 (m, 3H, ArH) ppm; ^{13}C NMR (100.6 MHz, $CDCl_3$): δ 14.2 (CH_3), 22.7 (CH_2), 28.3 (CH_3), 28.8 (CH_3), 31.5 (CH_2), 31.7 (CH_2), 38.2 (CH_2), 46.7 (CH), 75.1 (C), 125.0 (CH), 125.3 (CH), 126.4 (CH), 126.7 (CH), 127.0 (CH), 134.9 (C), 135.1 (C), 142.8 (C) ppm; LRMS (70 eV, EI): *m/z* (%) 227 ([M^+-OH], 16), 141 (46) 129 (100), 59 (62); HRMS (EI) calcd for $C_{17}H_{23}$ [M^+-OH]: 227.1800, found: 227.1801.

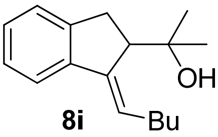

8i

(*E*)-2-(1-Pentylidene-2,3-dihydro-1*H*-inden-2-yl)propan-2-ol (8i):

Yellow oil; yield = 30%; R_f = 0.18 (hex/AcOEt 12:1); ^{1}H NMR (300 MHz, $CDCl_3$): δ 0.91 (t, *J* = 6.9 Hz, 3H, $\mathit{CH_3}CH_2CH_2CH_2$), 0.91 (s, 3H, CH_3), 1.18 (s, 3H, CH_3), 1.29−1.52 (m, 4H, $CH_3\mathit{CH_2CH_2}CH_2$) 1.63 (s, 1H, OH), 2.17−2.47 (m, 2H, $CH_3CH_2CH_2\mathit{CH_2}$), 2.81 (d, *J* = 16.2 Hz, 1H, C*H*H), 3.06 (dd, *J* = 16.2, 7.9 Hz, 1H,

CH*H*), 3.15 (d, *J* = 7.9 Hz, 1H, CH), 6.06 (ddd, *J* = 9.1, 5.8, 1.4 Hz, 1H, =CH), 7.04−7.26 (m, 3H, ArH), 7.31−7.47 (m, 1H, ArH) ppm; ^{13}C NMR (75.4 MHz, $CDCl_3$): δ 14.2 (CH_3), 22.6 (CH_2), 26.6 (CH_3), 27.5 (CH_3), 30.5 (CH_2), 32.4 (CH_2), 34.5 (CH_2), 51.8 (CH), 74.5 (C), 119.4 (CH), 124.8 (CH), 125.9 (CH), 126.6 (CH), 127.5 (CH), 142.1 (C), 143.0 (C), 144.2 (C) ppm; LRMS (70 eV, EI): *m/z* (%) 227 ([M^+–OH], 18), 173 (100), 132 (67); HRMS (EI) calcd for $C_{17}H_{23}$ [M^+–OH]: 227.1800, found: 227.1800.

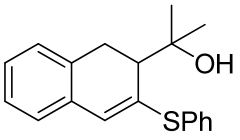

7k

2-(1,2-Dihydro-3-(phenylthio)naphthalen-2-yl)propan-2-ol (7k):

White solid; yield = 60%; R_f = 0.14 (hex/AcOEt 9:1). m.p. = 97−99 ºC; ^{1}H NMR (300 MHz, $CDCl_3$): δ 1.01 (s, 3H, CH_3), 1.20 (s, 3H, CH_3), 2.49−2.64 (m, 1H, CH), 2.54 (s, 1H, OH), 3.02 (dd, *J* = 16.5, 1.6 Hz, 1H, C*H*H), 3.17 (dd, *J* = 16.5, 7.8 Hz, 1H, CH*H*), 6.61 (s, 1H, =CH), 6.86−6.97 (m, 1H, ArH), 7.01−7.23 (m, 3H, ArH), 7.27−7.47 (m, 3H, ArH), 7.47−7.73 (m, 2H, ArH) ppm; ^{13}C NMR (75.4 MHz, $CDCl_3$): δ 27.5 (CH_3), 28.3 (CH_3), 31.9 (CH_2), 48.6 (CH), 74.7 (C), 125.5 (CH), 126.7 (CH), 127.2 (CH), 127.8 (CH), 128.1 (CH), 129.5 (2 × CH), 130.0 (CH), 132.5 (2 × CH), 133.3 (C), 133.9 (C), 134.1 (C), 136.1 (C) ppm; LRMS (70 eV, EI): *m/z* (%) 296 (M^+, 4), 238 (74), 128 (100); HRMS (EI) calcd for $C_{19}H_{20}OS$: 296.1235, found: 296.1235.

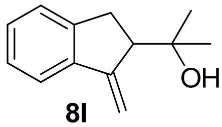

8l

2-(1-Methylene-2,3-dihydro-1*H*-inden-2-yl)propan-2-ol (8l):

Yellow oil; yield = 55%; R_f = 0.16 (hex/AcOEt 9:1); ^{1}H NMR (300 MHz, $CDCl_3$): δ 1.02 (s, 3H, CH_3), 1.21 (s, 3H, CH_3), 1.95 (s, 1H, OH), 2.85 (dd, *J* = 16.7, 1.8 Hz, 1H, CH),

3.00 (m, 1H, C*H*H), 3.15 (dd, *J* = 16.7, 8.5 Hz, 1H, CH*H*), 5.22 (s, 1H, =C*H*H), 5.65 (s, 1H, =CH*H*), 7.05−7.34 (m, 3H, ArH), 7.38−7.57 (m, 1H, ArH) ppm; ^{13}C NMR (75.4 MHz, $CDCl_3$): δ 25.5 (CH_3), 27.4 (CH_3), 34.2 (CH_2), 54.2 (CH), 72.9 (C), 106.5 (CH_2), 120.4 (CH), 125.1 (CH), 126.7 (CH), 128.7 (CH), 141.5 (C), 145.4 (C), 151.2 (C) ppm; LRMS (70 eV, EI): *m/z* (%) 186 (M^+ ,62), 129 (100), 83 (88), 59 (83); HRMS (EI) calcd for $C_{13}H_{16}O$: 186.1409, found: 186.1412.

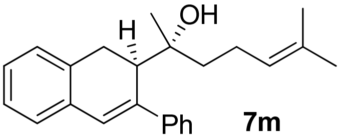

7m

6-Methyl-2-(3-phenyl-1,2-dihydronaphthalen-2-yl)hept-5-en-2-ol (7m):

Colourless oil; yield = 69%; R_f = 0.22 (hex/AcOEt 9:1); ^{1}H NMR (400 MHz, $CDCl_3$): δ 0.92 (s, 3H, CH_3), 1.27 (s, 1H, OH), 1.33 (m, 2H, CH_2), 1.54 (s, 3H, CH_3), 1.64 (s, 3H, CH_3), 1.82−2.02 (m, 2H, CH_2), 3.14 (dd, *J* = 7.7, 1.2 Hz, 1H, CH), 3.25 (dd, *J* = 16.4, 7.7 Hz, 1 H, C*H*H), 3.34 (dd, *J* = 16.4, 1.2 Hz, 1 H, CH*H*), 4.81−4.97 (m, 1H, =CH), 6.85 (s, 1H, =CH), 7.02−7.23 (m, 4H, ArH), 7.23−7.35 (m, 1H, ArH), 7.35−7.47 (m, 2H, ArH), 7.47−7.67 (m, 2H, ArH) ppm; ^{13}C NMR (100.6 MHz, $CDCl_3$): δ 17.8 (CH_3), 22.5 (CH_2), 25.1 (CH_3), 25.8 (CH_3), 30.5 (CH_2), 40.5 (CH_2), 43.8 (CH), 77.0 (C), 124.6 (CH), 126.3 (CH), 126.5 (CH), 126.7 (2 × CH), 127.0 (CH), 127.3 (CH), 127.7 (CH), 128.5 (CH), 128.7 (2 × CH), 131.6 (C), 134.6 (C), 135.4 (C), 140.2 (C), 143.3 (C) ppm; LRMS (70 eV, EI): *m/z* (%) 332 (M^+ ,0.7), 206 (100), 109 (18); HRMS (EI) calcd for $C_{24}H_{28}O$: 332.2140, found: 332.2144.

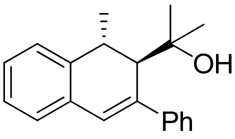

7n

2-(1-Methyl-3-phenyl-1,2-dihydronaphthalen-2-yl)propan-2-ol (7n):

Colourless oil; yield = 57%. R_f = 0.12 (hex/AcOEt 10:1); ^{1}H NMR (300 MHz, $CDCl_3$): δ 0.91 (s, 3H, CH_3), 0.97 (s, 3H, CH_3), 1.29 (s, 1H, OH), 1.30 (d, *J* = 7.2 Hz, 3H, CH_3), 2.90 (s, 1H, CH), 3.41 (q, *J* = 7.1 Hz, 1H, CH), 6.83 (s, 1H, =CH), 7.04−7.23 (m, 4H, ArH), 7.23−7.34 (m, 1H, ArH), 7.35−7.46 (m, 2H, ArH), 7.46−7.62 (m, 2H, ArH) ppm; ^{13}C NMR (75.4 MHz, $CDCl_3$): δ 23.7 (CH_3), 27.3 (CH_3), 29.3 (CH_3), 34.6 (CH), 53.8 (CH), 75.2 (C), 126.3 (2 × CH), 126.5 (CH), 126.8 (CH), 127.1 (CH), 127.2 (CH), 127.3 (CH), 128.0 (CH), 128.7 (2 × CH), 132.9 (C), 137.7 (C), 141.1 (C), 144.0 (C) ppm; LRMS (70 eV, EI): *m/z* (%) 219 ([M^+–$C(CH_3)_2OH$], 84), 205 (100), 59 (13). HRMS (EI) calcd for $C_{20}H_{22}O$: 278.1671, found: 278.1668.

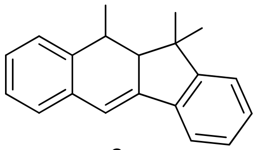

3n

10,11,11-Trimethyl-10*a*,11-dihydro-10*H*-benzo[*b*]fluorene (3n):

Colorless oil; yield = 25%; R_f = 0.74 (hex/AcOEt 10:1); ^{1}H NMR (300 MHz, $CDCl_3$): δ 0.97 (s, 3H, CH_3), 1.49 (d, *J* = 6.8 Hz, 3H, CH_3), 1.60 (s, 3H, CH_3), 2.67 (dd, *J* = 6.0, 3.0 Hz, 1H, CH), 3.28 (quint, *J* = 6.7 Hz, 1H, CH), 6.88 (d, *J* = 3.0 Hz, 1H, =CH), 7.14−7.37 (m, 6H, ArH), 7.37−7.47 (m, 1H, ArH), 7.55−7.68 (m, 1H, ArH) ppm; ^{13}C NMR (75.4 MHz, $CDCl_3$): δ 22.50 (CH_3), 22.55 (CH_3), 26.0 (CH), 37.9 (CH), 38.0 (C), 56.8 (CH), 115.4 (CH), 120.7 (CH), 123.5 (CH), 124.6 (CH), 126.5 (CH), 127.0 (2 × CH), 127.1 (CH), 128.7 (CH), 134.5 (C), 139.0 (C), 144.97 (C), 145.02 (C), 151.9 (C) ppm; LRMS (70 eV, EI): *m/z* (%) 260 (M^+, 50), 245 (100), 215 (52); HRMS (EI) calcd for $C_{20}H_{20}$: 260.1565, found: 260.1562.

General procedure for the gold-catalyzed methoxycyclization of selected 1,6-enynes 1:

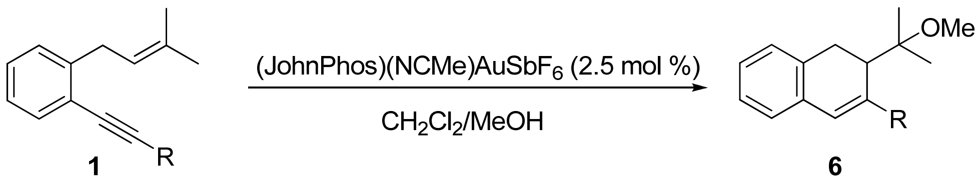

MeOH (3 equiv, 42 μL) was added to a solution of (JohnPhos)(NCMe)AuSbF$_6$ (2.5 mol %, 6 mg) in DCM (0.6 mL). After stirring at rt for 5−10 min a solution of the corresponding enyne **1** (0.3 mmol) in DCM (0.6 mL) was added and the resulting mixture was stirred overnight (complete consumption of starting material was confirmed by TLC). The solution was diluted with hex/EtOAc: 9/1 and filtered through a pad of celite. The solvent was removed and the crude residue was purified by column chromatography on silica gel using mixtures of hexane and EtOAc as eluents to afford the corresponding dihydronaphthalene derivatives **6** in the yields reported in Scheme 7.

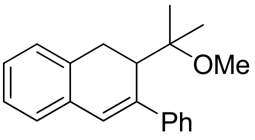

6a

2-(2-Methoxypropan-2-yl)-3-phenyl-1,2-dihydronaphthalene (6a):

Yellow solid; yield = 75%. Contaminated with trace amounts of the corresponding 5-membered isomer (derived from the 5-*exo* cyclization). R_f = 0.49 (hex/AcOEt 9:1); ^{1}H NMR (300 MHz, CDCl$_3$): δ 0.72 (s, 3H, CH$_3$), 0.85 (s, 3H, CH$_3$), 2.97−3.25 (m, 1H, C*H*H), 3.17 (s, 3H, OCH$_3$), 3.29 (d, *J* = 8.5 Hz, 1H, CH), 3.38 (d, *J* = 16.2 Hz, 1H, CH*H*), 6.79 (s, 1H, =CH), 7.03−7.23 (m, 4H, ArH), 7.24−7.33 (m, 1H, ArH), 7.33−7.46 (m, 2H, ArH), 7.47−7.65 (m, 2H, ArH) ppm; ^{13}C NMR (75.4 MHz, CDCl$_3$): δ 22.4 (CH$_3$), 25.1 (CH$_3$), 29.6 (CH$_2$), 41.6 (CH), 49.0 (CH$_3$), 79.0 (C), 126.28 (CH), 126.29

(CH), 126.7 (2 × CH), 127.1 (CH), 127.3 (CH), 127.6 (CH), 128.5 (2 × CH), 128.6 (CH), 134.6 (C), 135.8 (C), 140.4 (C), 143.8 (C) ppm; LRMS (70 eV, EI): *m/z* (%) 278 (M^+, 2), 73 (100), 204 (17); HRMS (EI) calcd for $C_{20}H_{22}O$: 278.1671, found: 278.1670.

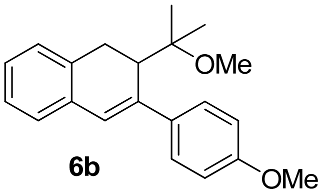

6b

3-(4-Methoxyphenyl)-2-(2-methoxypropan-2-yl)-1,2-dihydronaphthalene (6b): White solid; yield = 73%; R_f = 0.49 (hex/AcOEt 10:1); m.p. = 88−90 ºC; ^{1}H NMR (300 MHz, $CDCl_3$): δ 0.74 (s, 3H, CH_3), 0.85 (s, 3H, CH_3), 2.97−3.30 (m, 2H, C*H*H and CH), 3.18 (s, 3H, OCH_3), 3.38 (d, *J* = 16.1 Hz, 1H, CH*H*), 3.84 (s, 3H, OCH_3), 6.74 (s, 1H, =CH), 6.93 (d, *J* = 8.8 Hz, 2H), 7.04−7.26 (m, 4H, ArH), 7.45 (d, *J* = 8.8 Hz, 2H) ppm; ^{13}C NMR (75.4 MHz, $CDCl_3$): δ 22.4 (CH_3), 25.2 (CH_3), 29.6 (CH_2), 41.6 (CH), 49.0 (CH_3), 55.3 (CH_3), 79.0 (C), 113.9 (2 × CH), 126.0 (CH), 126.2 (CH), 127.17 (CH), 127.23 (CH), 127.3 (CH), 127.8 (2 × CH), 134.8 (C), 135.7 (C), 136.2 (C), 139.9 (C), 158.9 (C) ppm; LRMS (70 eV, EI): *m/z* (%) 308 (M^+, <2), 234 (18), 73 (100); HRMS (EI) calcd for $C_{21}H_{24}O_2$: 308.1776, found: 308.1764.

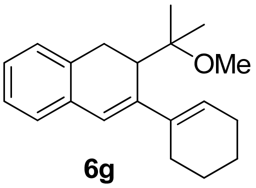

6g

2-(3-(Cyclohex-1-en-1-yl)-2-(2-methoxypropan-2-yl)-1,2-dihydronaphthalene (6g): White solid; yield = 70%. R_f = 0.45 (hex/AcOEt 10:1); m.p. = 81−83 ºC; ^{1}H NMR (300 MHz, $CDCl_3$): δ 0.78 (s, 3H, CH_3), 0.99 (s, 3H, CH_3), 1.49−1.89 (m, 4H, *c*-C_6H_9), 2.04−2.30 (m, 3H, *c*-C_6H_9), 2.43−2.61 (m, 1H, *c*-C_6H_9), 2.94 (dd, *J* = 16.1, 8.1 Hz,

1H, C*H*H), 3.04 (d, J = 8.1 Hz, 1H, CH), 3.15−3.32 (m, 1H, CH*H*), 3.22 (s, 3H, OCH_3), 5.95−6.06 (m, 1H, =CH), 6.56 (s, 1H, =CH), 6.95−7.19 (m, 4H, ArH) ppm; ^{13}C NMR (75.4 MHz, $CDCl_3$): δ 22.5 (CH_2), 22.8 (CH_3), 23.3 (CH_2), 24.5 (CH_3), 26.1 (CH_2), 27.5 (CH_2), 29.6 (CH_2), 38.9 (CH), 49.1 (CH_3), 79.1 (C), 123.7 (CH), 125.3 (CH), 126.06 (CH), 126.13 (CH), 127.01 (CH), 127.04 (CH), 135.0 (C), 136.2 (C), 137.9 (C), 141.3 (C) ppm; LRMS (70 eV, EI): *m/z* (%) 282 (M^+, <2), 73 (100); HRMS (EI) calcd for $C_{20}H_{26}O$: 282.1984, found: 226.1992.

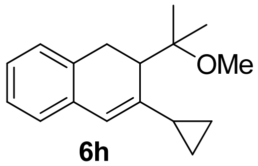

6h

3-Cyclopropyl-2-(2-methoxypropan-2-yl)-1,2-dihydronaphthalene (6h):
Colourless oil; yield = 80%. Isolated as a 4:1 mixture of the title compound and the corresponding 5-membered isomer (derived from the 5-*exo* cyclization). R_f = 0.49 (hex/AcOEt 10:1); ^{1}H NMR (300 MHz, $CDCl_3$): δ 0.44−0.61 (m, 2H, *c*-C_3H_5), 0.69−0.94 (m, 2H, *c*-C_3H_5), 0.96 (s, 3H, CH_3), 1.12 (s, 3H, CH_3), 1.47−1.62 (m, 1H, *c*-C_3H_5), 2.56−2.71 (m, 1H, C*H*H), 2.85−2.15 (m, 2H, CH*H* and CH), 3.25 (s, 3H, OCH_3), 6.10 (s, 1H, =CH), 6.85−6.95 (m, 1H, ArH), 6.96−7.32 (m, 3H, ArH) ppm; ^{13}C NMR (75.4 MHz, $CDCl_3$): δ 8.47 (CH_2), 9.42 (CH_2), 17.93 (CH), 23.1 (CH_3), 23.9 (CH_3), 30.3 (CH_2), 45.5 (CH), 49.0 (CH_3), 78.7 (C), 120.7 (CH), 125.2 (CH), 126.2 (CH), 126.5 (CH), 126.7 (CH), 134.8 (C), 135.0 (C), 144.4 (C) ppm; LRMS (70 eV, EI): *m/z* (%) 168 ([M^+–$C(CH_3)_2OCH_3$], <2), 73 (100); HRMS (EI) calcd for $C_{13}H_{13}$ [M^+–$C(CH_3)_2OCH_3$]: 168.0939, found: 168.0939.

References

1. Gawade, S. A.; Bhunia, S.; Liu, R.-S. *Angew. Chem. Int. Ed.* **2012**, *51*, 7835–7838.
