# Supplementary material for: Gold(I)-catalyzed 6-endo hydroxycyclization of 7-substituted-1,6-enynes
Source: Beilstein J Org Chem. 2013 Oct 29;9:2242–9. doi: 10.3762/bjoc.9.263 (PMC3817472; doi:10.3762/bjoc.9.263)
Supplement: File 2 — NMR spectra. [file Beilstein_J_Org_Chem-09-2242-s002.pdf]

# Supporting Information

for

# Gold(I)-catalyzed 6-*endo* hydroxycyclization of 7-substituted-1,6-enynes

Ana M. Sanjuán, Alberto Martínez, Patricia García-García, Manuel A. Fernández-Rodríguez and Roberto Sanz*

Address: Área de Química Orgánica, Departamento de Química, Facultad de Ciencias, Universidad de Burgos, Pza. Misael Bañuelos s/n, 09001 Burgos, Spain

Email: Roberto Sanz - rsd@ubu.es

* Corresponding author

## NMR spectra

## $^{1}H$ and $^{13}C$ NMR spectra

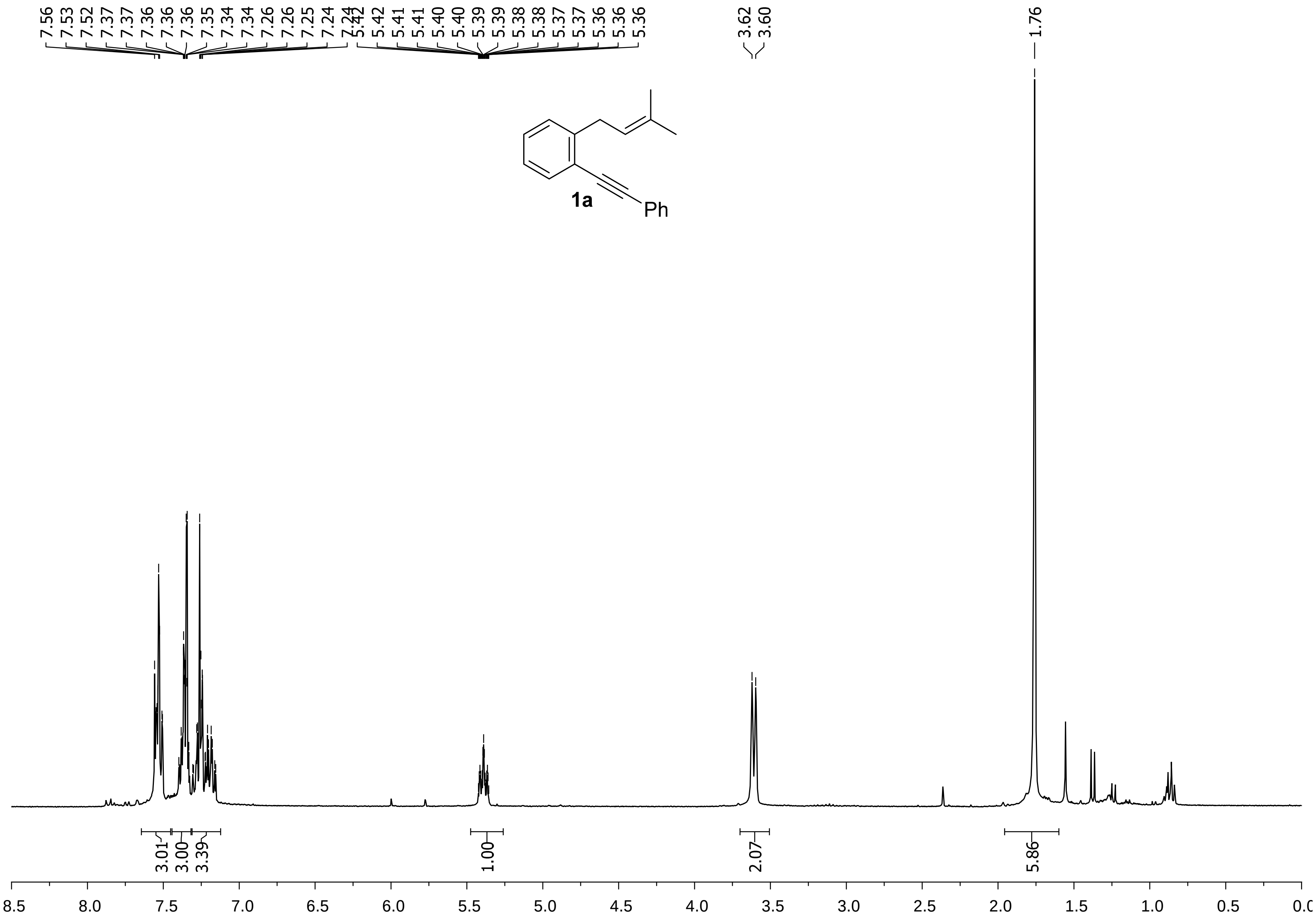

7.56 7.53 7.52 7.37 7.37 7.36 7.36 7.36 7.35 7.34 7.34 7.26 7.26 7.25 7.24 7.24
5.42 5.42 5.41 5.41 5.40 5.40 5.39 5.39 5.38 5.38 5.37 5.37 5.36 5.36 5.36
3.62 3.60
1.76
1a
Ph
3.01 3.00 3.39
1.00
2.07
5.86
8.5 8.0 7.5 7.0 6.5 6.0 5.5 5.0 4.5 4.0 3.5 3.0 2.5 2.0 1.5 1.0 0.5

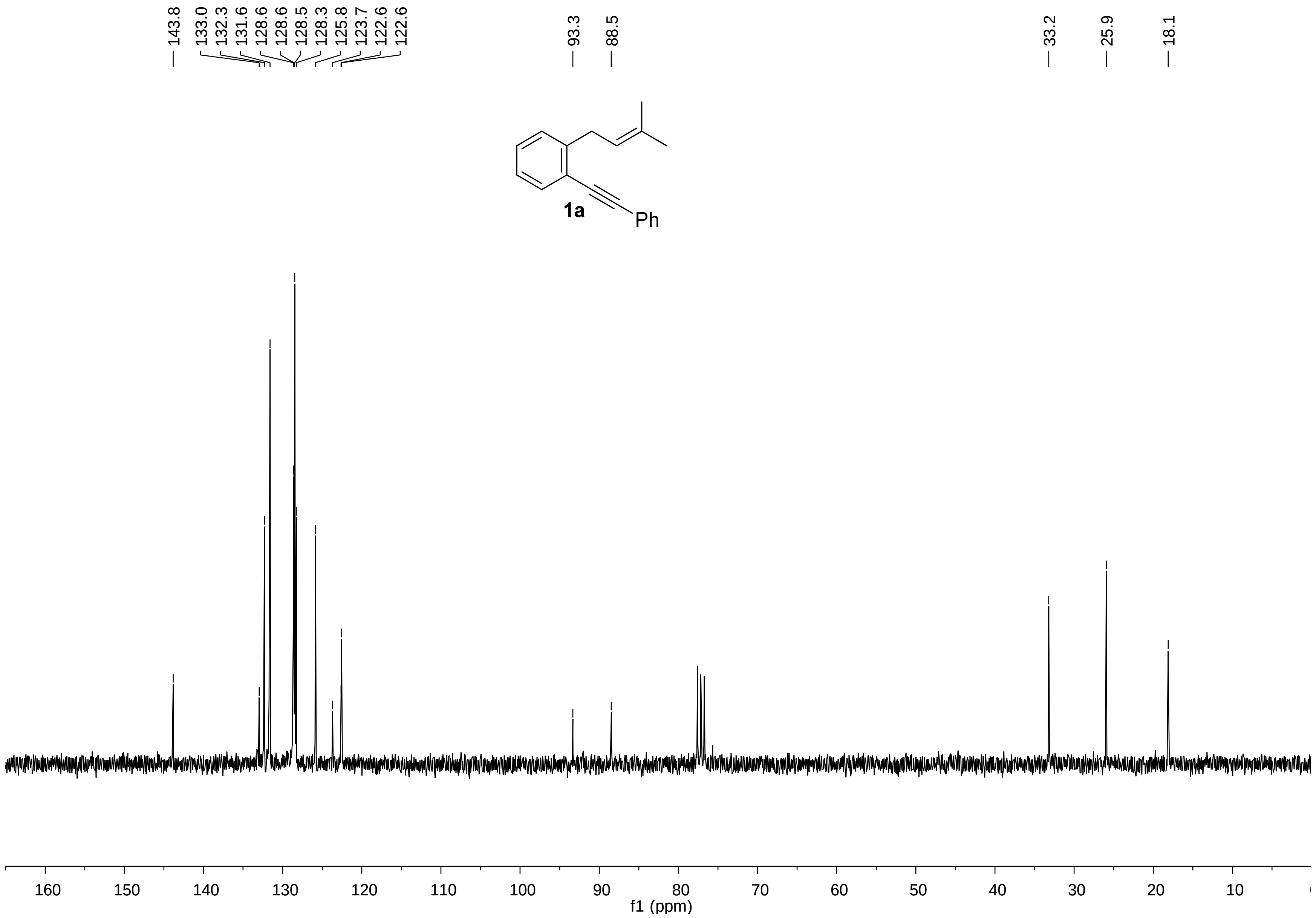
143.8
133.0
132.3
131.6
128.6
128.6
128.5
128.3
125.8
123.7
122.6
122.6
93.3
88.5
33.2
25.9
18.1
1a
Ph
f1 (ppm)

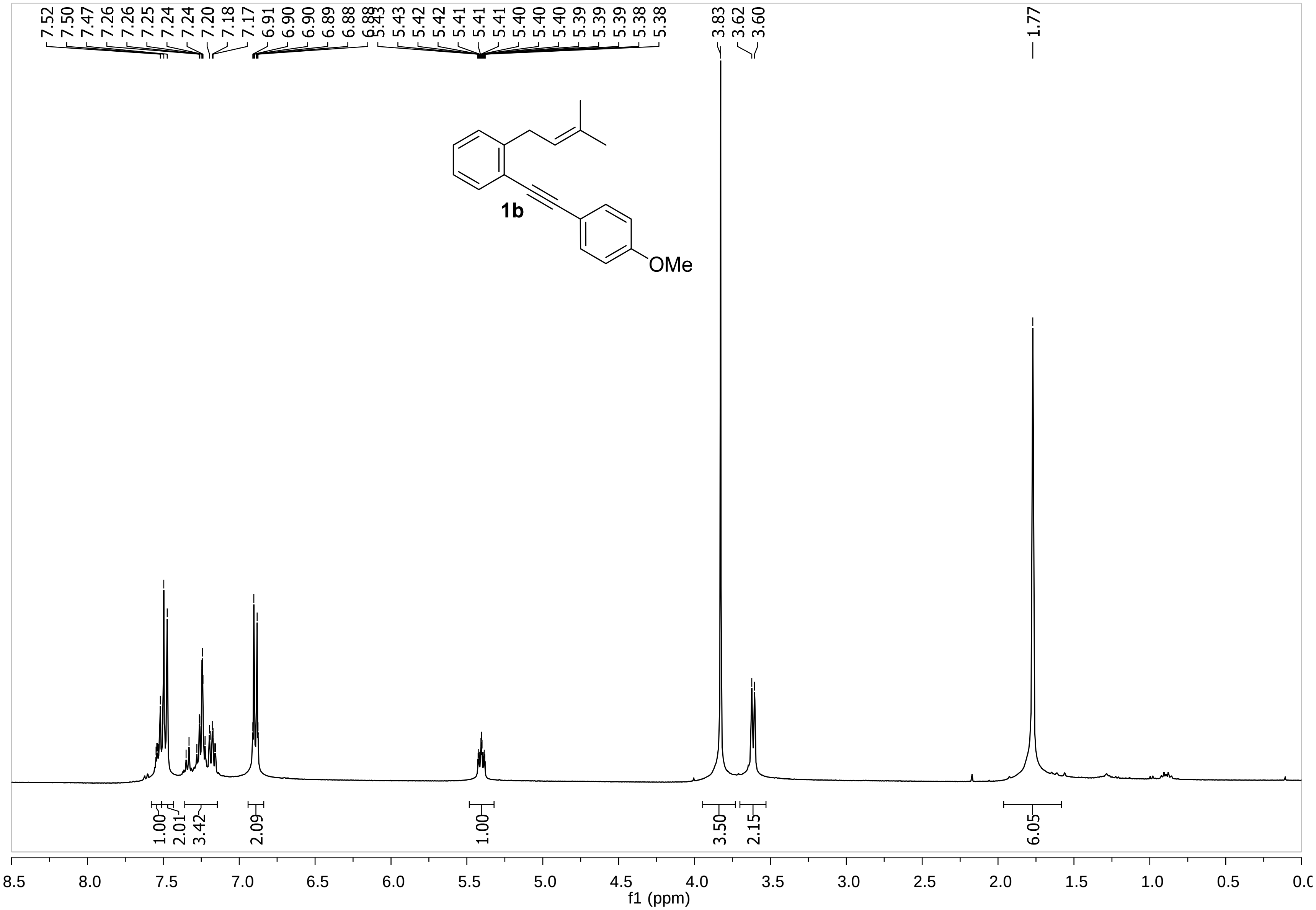
7.52 7.50 7.47 7.26 7.26 7.25 7.24 7.24 7.20 7.18 7.17 6.91 6.90 6.90 6.89 6.88 6.88 5.43 5.43 5.42 5.42 5.41 5.41 5.41 5.40 5.40 5.40 5.39 5.39 5.39 5.38 5.38
3.83 3.62 3.60
1.77
1b
OMe
1.00 2.01 3.42 2.09 1.00 3.50 2.15 6.05
8.5 8.0 7.5 7.0 6.5 6.0 5.5 5.0 4.5 4.0 3.5 3.0 2.5 2.0 1.5 1.0 0.5
f1 (ppm)

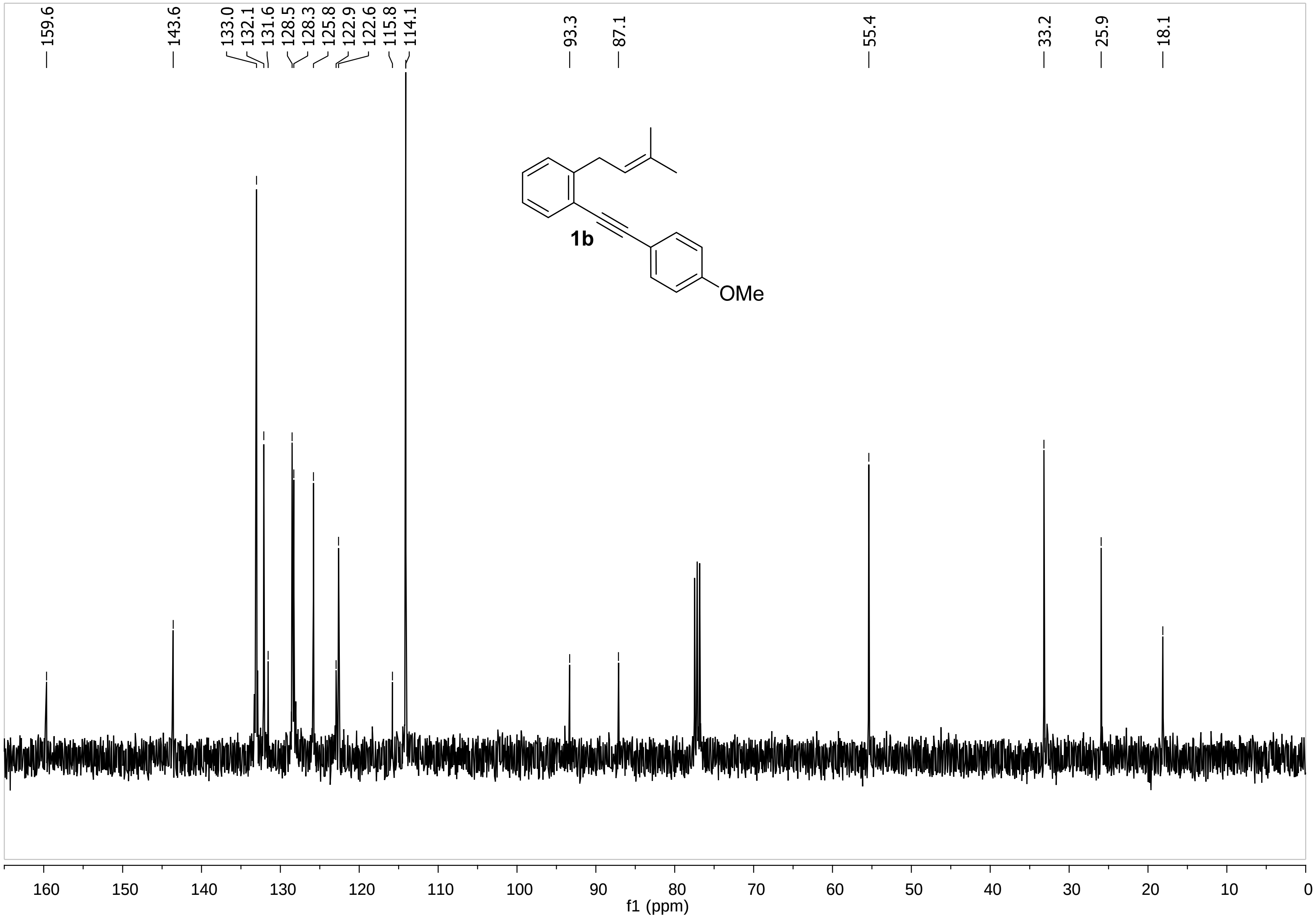
159.6
143.6
133.0
132.1
131.6
128.5
128.3
125.8
122.9
122.6
115.8
114.1
93.3
87.1
55.4
33.2
25.9
18.1
1b
OMe
160 150 140 130 120 110 100 90 80 70 60 50 40 30 20 10 0
f1 (ppm)

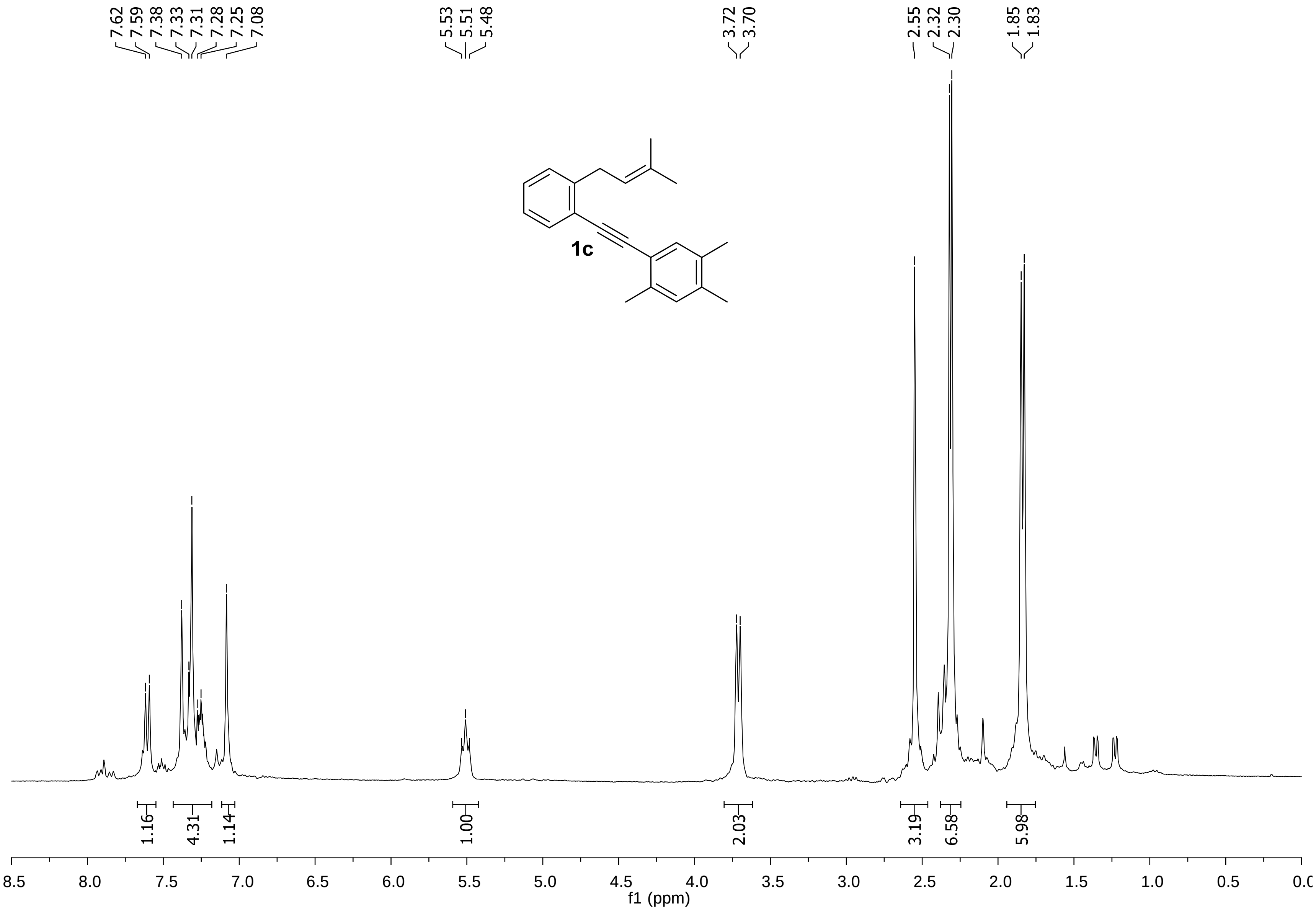
7.62
7.59
7.38
7.33
7.31
7.28
7.25
7.08
5.53
5.51
5.48
3.72
3.70
2.55
2.32
2.30
1.85
1.83
1c
1.16
4.31
1.14
1.00
2.03
3.19
6.58
5.98
8.5 8.0 7.5 7.0 6.5 6.0 5.5 5.0 4.5 4.0 3.5 3.0 2.5 2.0 1.5 1.0 0.5
f1 (ppm)

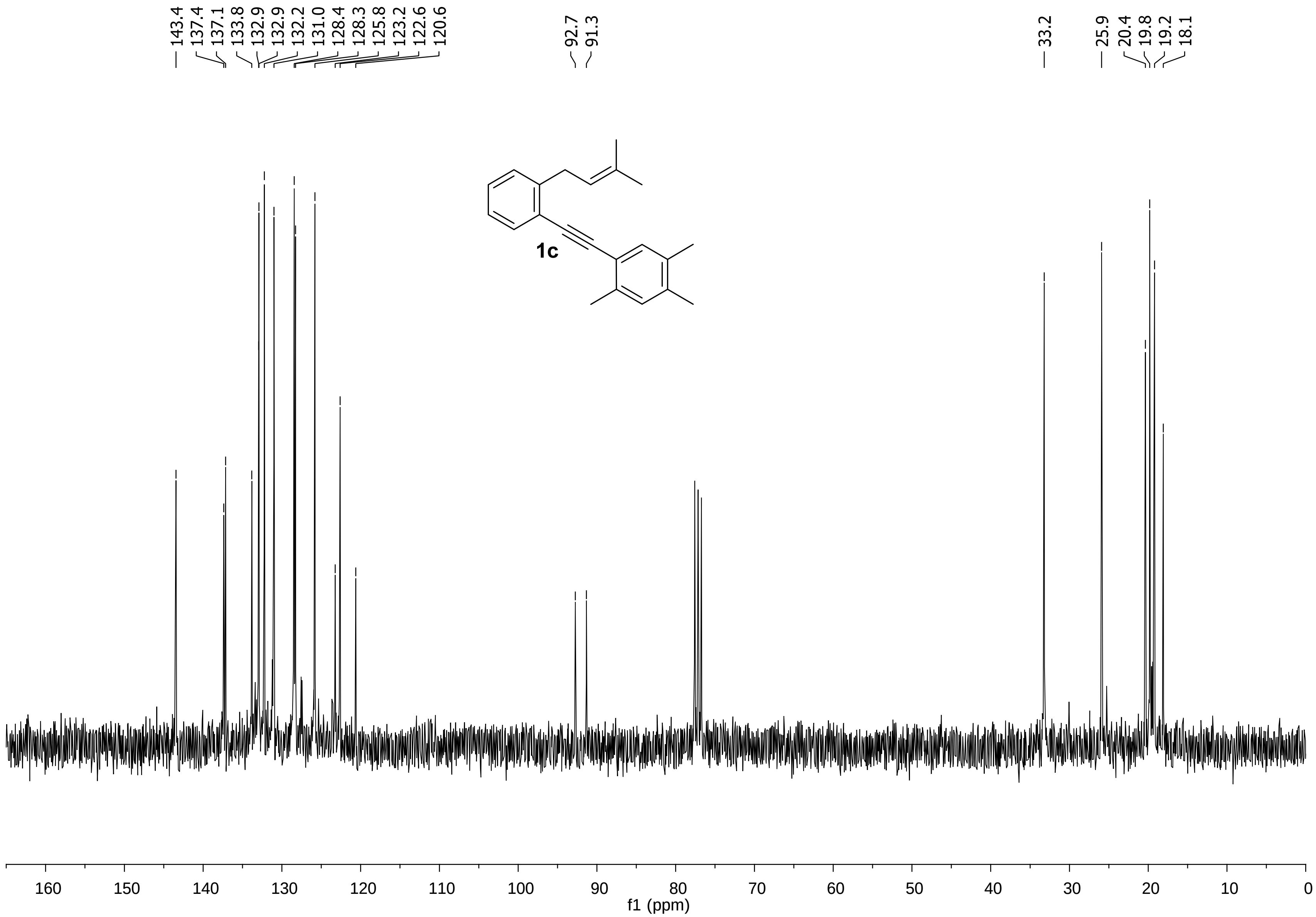
143.4
137.4
137.1
133.8
132.9
132.9
132.2
131.0
128.4
128.3
125.8
123.2
122.6
120.6
92.7
91.3
33.2
25.9
20.4
19.8
19.2
18.1
1c
160
150
140
130
120
110
100
90
80
70
60
50
40
30
20
10
0
f1 (ppm)

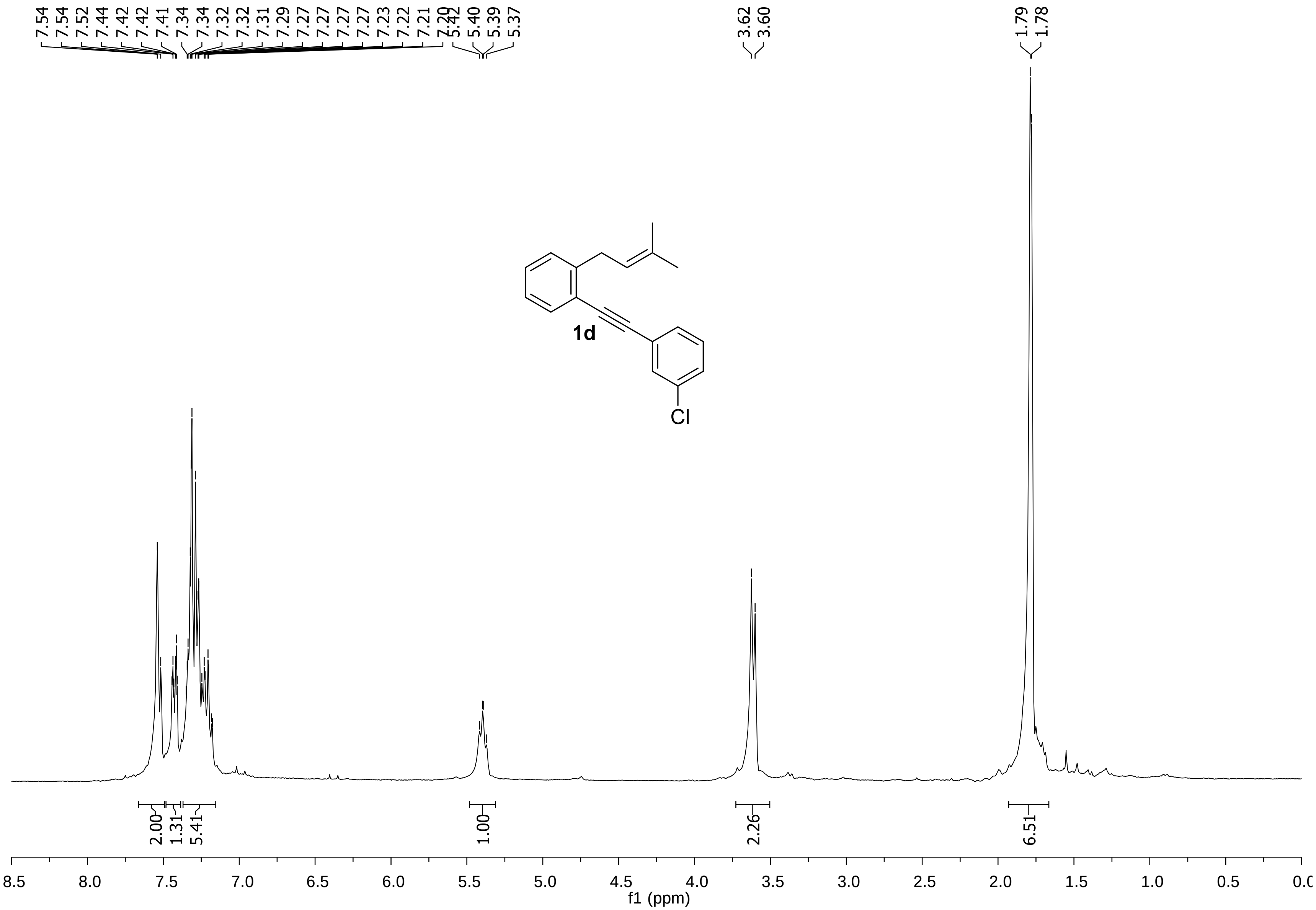
7.54
7.54
7.52
7.44
7.42
7.42
7.41
7.34
7.34
7.32
7.32
7.31
7.29
7.27
7.27
7.27
7.27
7.23
7.22
7.21
7.20
5.42
5.40
5.39
5.37
3.62
3.60
1.79
1.78
1d
Cl
2.00
1.31
5.41
1.00
2.26
6.51
8.5
8.0
7.5
7.0
6.5
6.0
5.5
5.0
4.5
4.0
3.5
3.0
2.5
2.0
1.5
1.0
0.5
f1 (ppm)

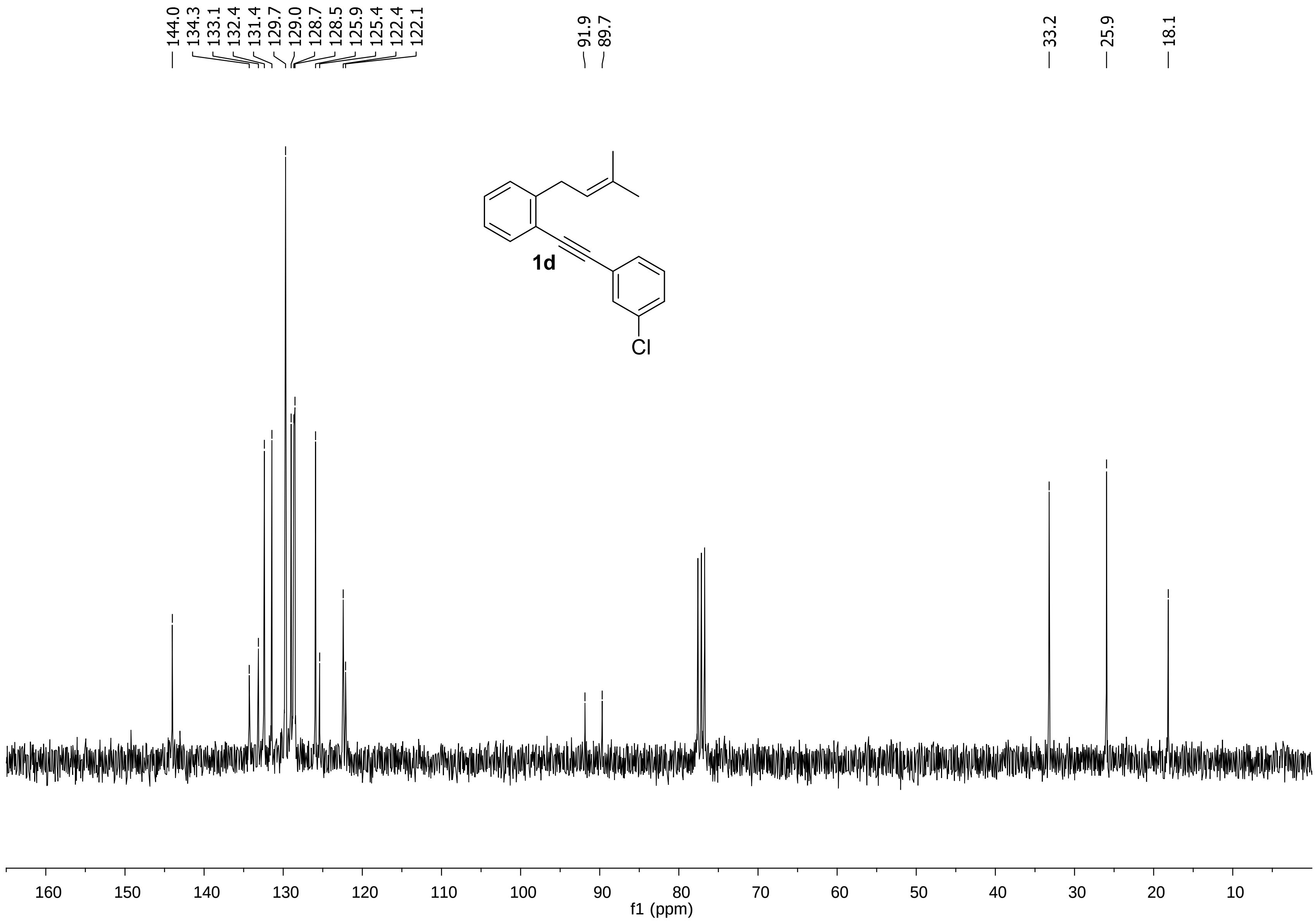
144.0
134.3
133.1
132.4
131.4
129.7
129.0
128.7
128.5
125.9
125.4
122.4
122.1
91.9
89.7
33.2
25.9
18.1
1d
Cl
160 150 140 130 120 110 100 90 80 70 60 50 40 30 20 10
f1 (ppm)

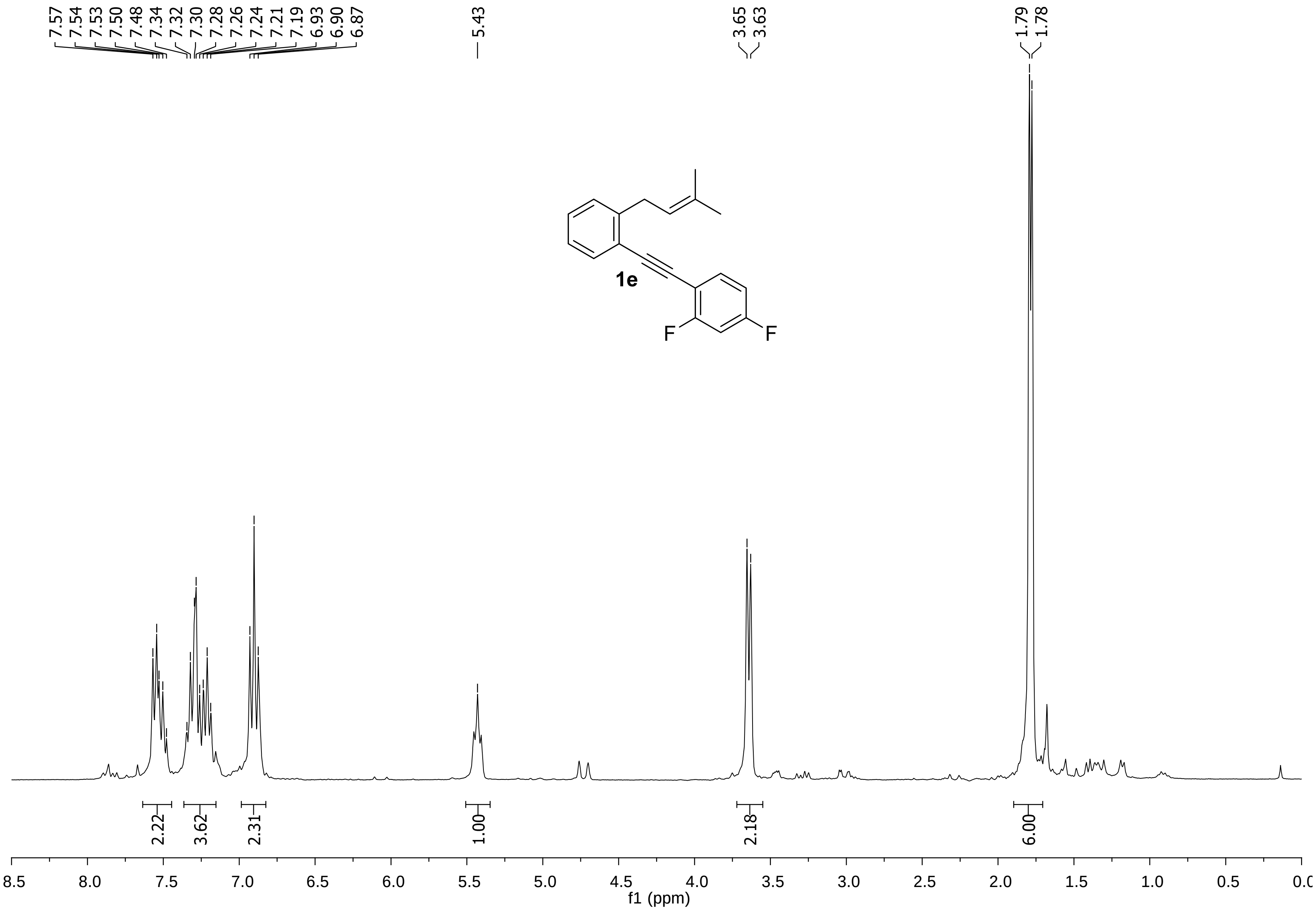
7.57 7.54 7.53 7.50 7.48 7.34 7.32 7.30 7.28 7.26 7.24 7.21 7.19 6.93 6.90 6.87
5.43
3.65 3.63
1.79 1.78
1e
F
F
2.22
3.62
2.31
1.00
2.18
6.00
8.5 8.0 7.5 7.0 6.5 6.0 5.5 5.0 4.5 4.0 3.5 3.0 2.5 2.0 1.5 1.0 0.5
f1 (ppm)

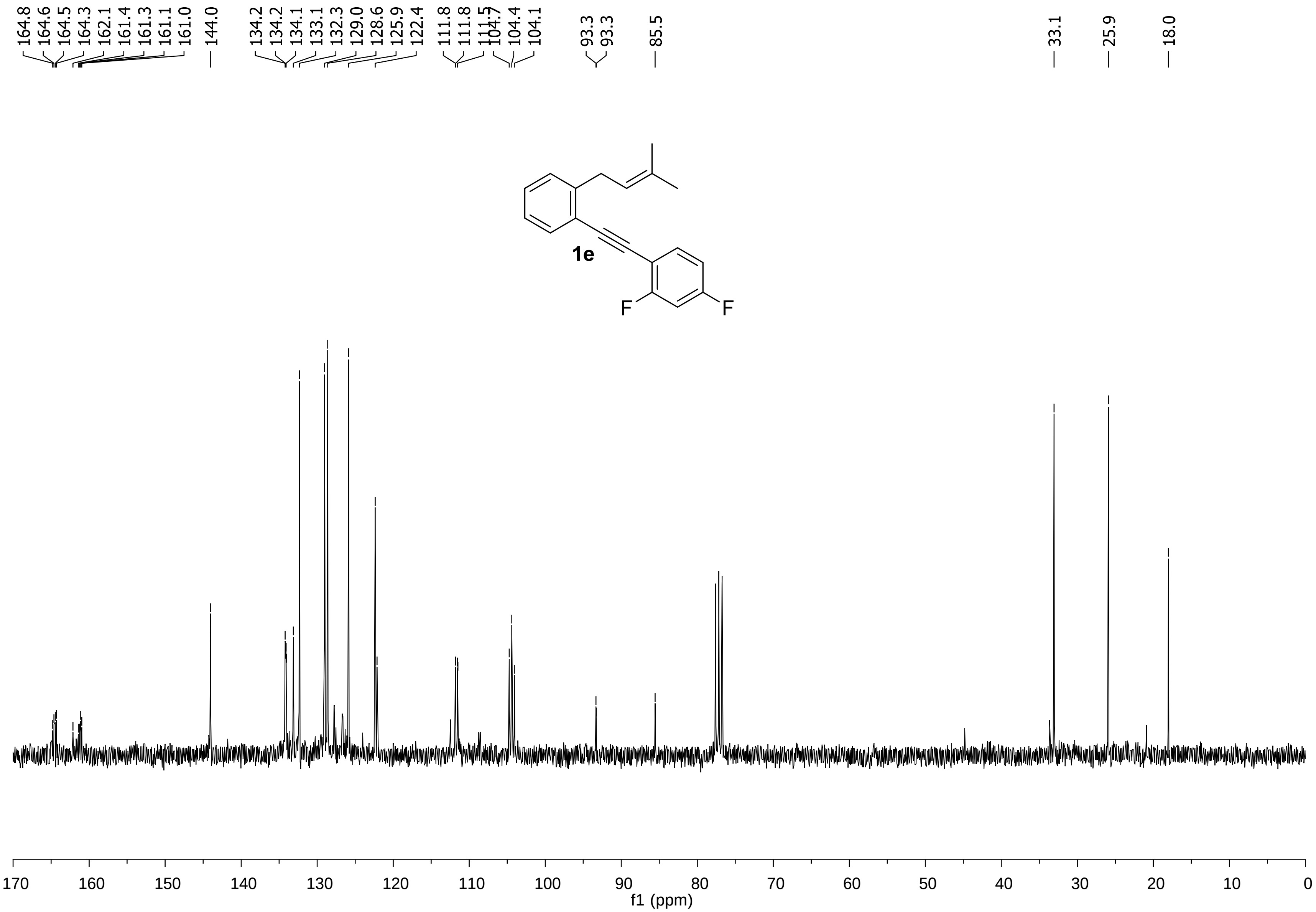
164.8
164.6
164.5
164.3
162.1
161.4
161.3
161.1
161.0
144.0
134.2
134.2
134.1
133.1
132.3
129.0
128.6
125.9
122.4
111.8
111.8
111.5
104.7
104.4
104.1
93.3
93.3
85.5
33.1
25.9
18.0
1e
F
F
170
160
150
140
130
120
110
100
90
80
70
60
50
40
30
20
10
0
f1 (ppm)

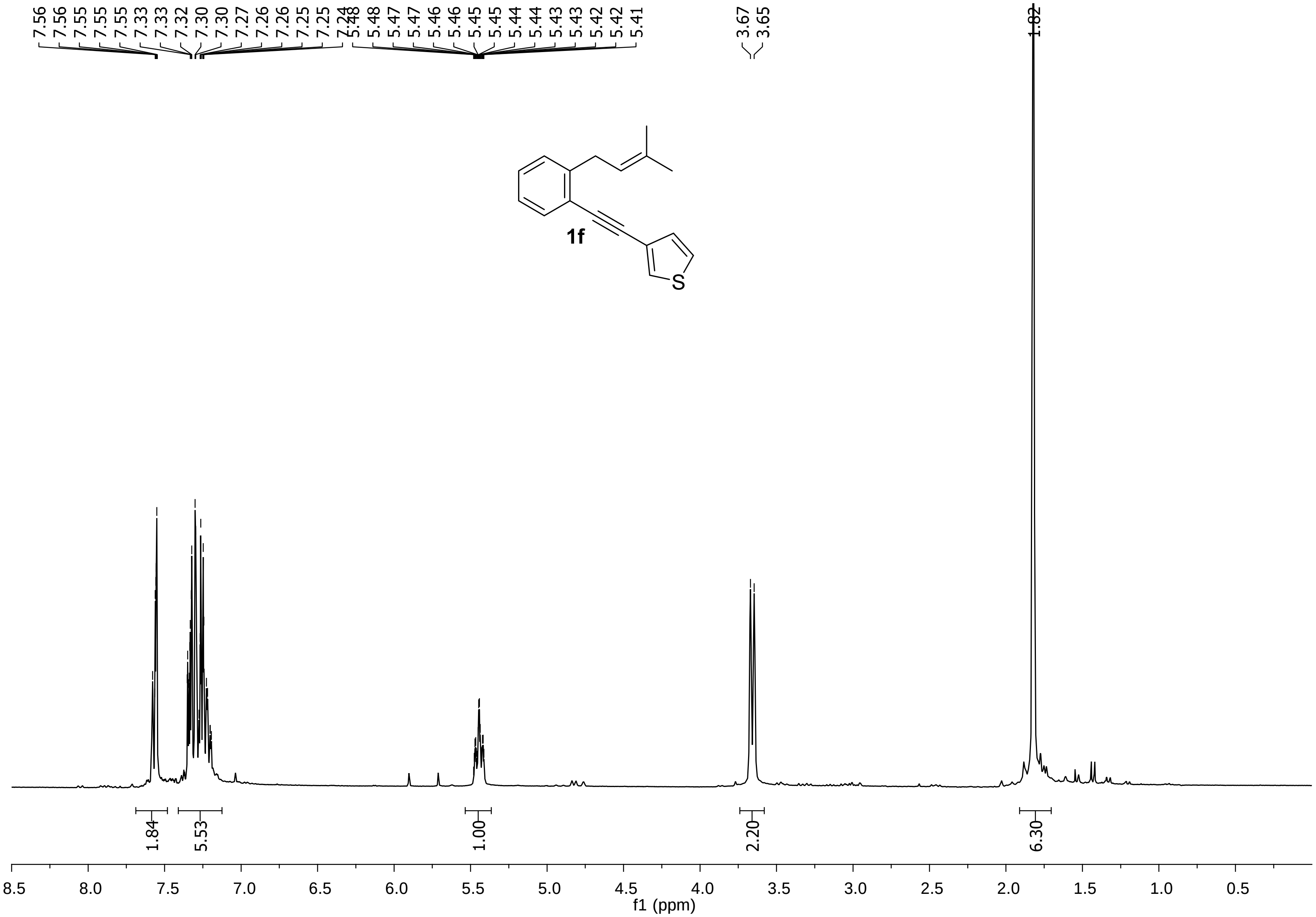
7.56 7.56 7.55 7.55 7.55 7.33 7.33 7.32 7.30 7.30 7.27 7.26 7.26 7.25 7.25 7.24 5.48 5.48 5.47 5.47 5.46 5.46 5.45 5.45 5.44 5.44 5.43 5.43 5.42 5.42 5.41
3.67 3.65
1.82
1f
S
1.84
5.53
1.00
2.20
6.30
8.5 8.0 7.5 7.0 6.5 6.0 5.5 5.0 4.5 4.0 3.5 3.0 2.5 2.0 1.5 1.0 0.5
f1 (ppm)

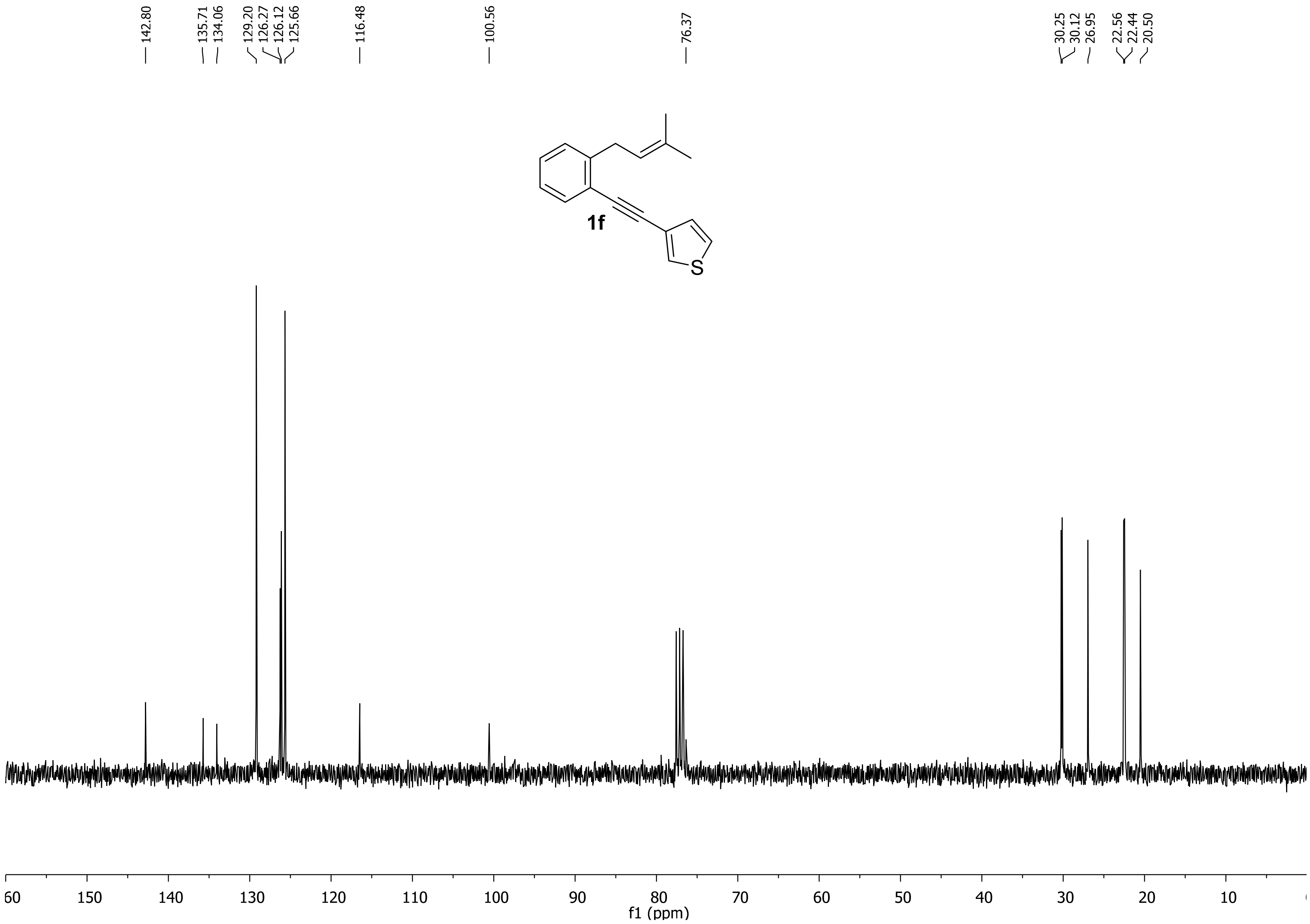
142.80
135.71
134.06
129.20
126.27
126.12
125.66
116.48
100.56
76.37
30.25
30.12
26.95
22.56
22.44
20.50
1f
S
60
150
140
130
120
110
100
90
80
70
60
50
40
30
20
10
f1 (ppm)

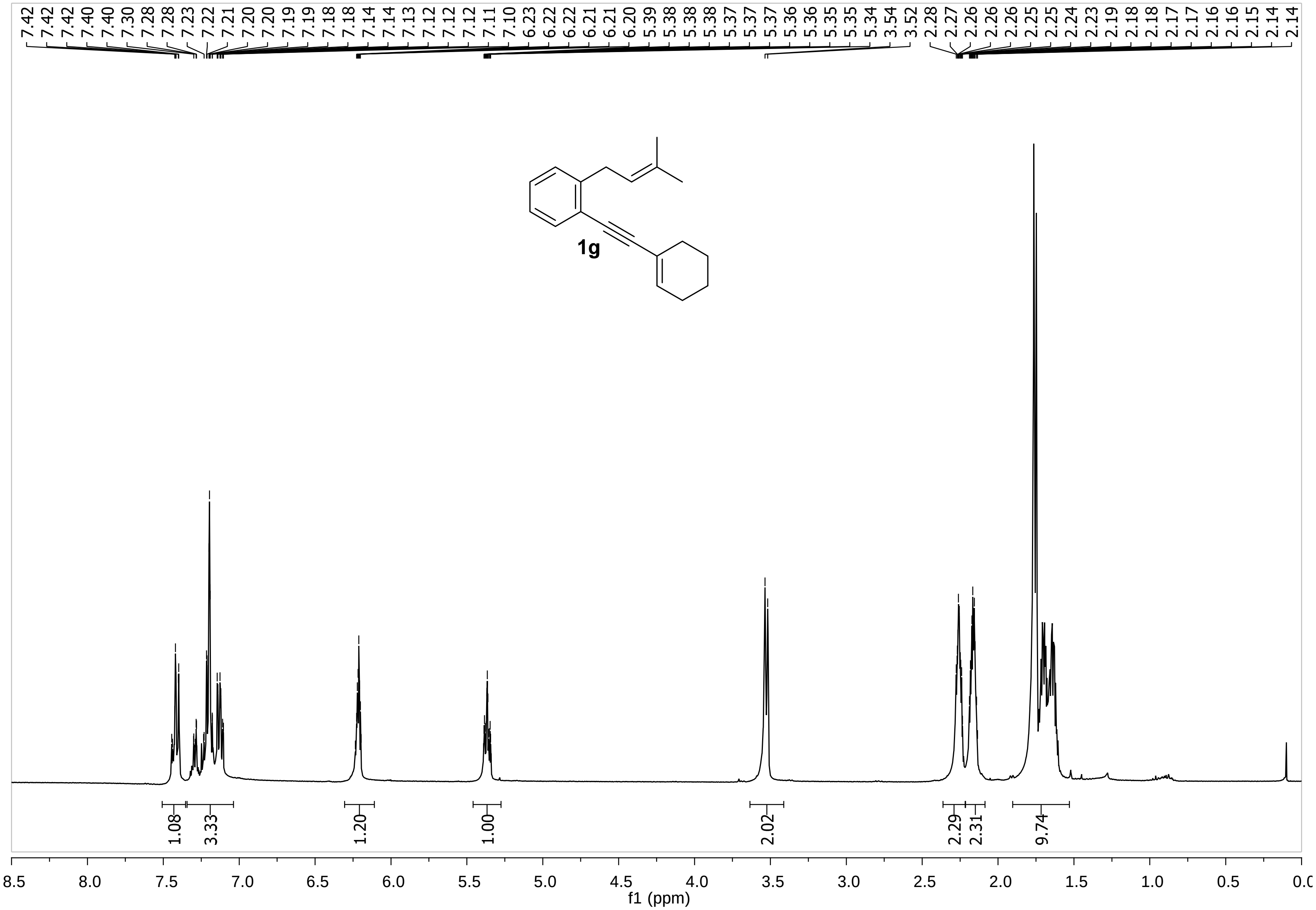
1g
f1 (ppm)
8.5
8.0
7.5
7.0
6.5
6.0
5.5
5.0
4.5
4.0
3.5
3.0
2.5
2.0
1.5
1.0
0.5
1.08
3.33
1.20
1.00
2.02
2.29
2.31
9.74

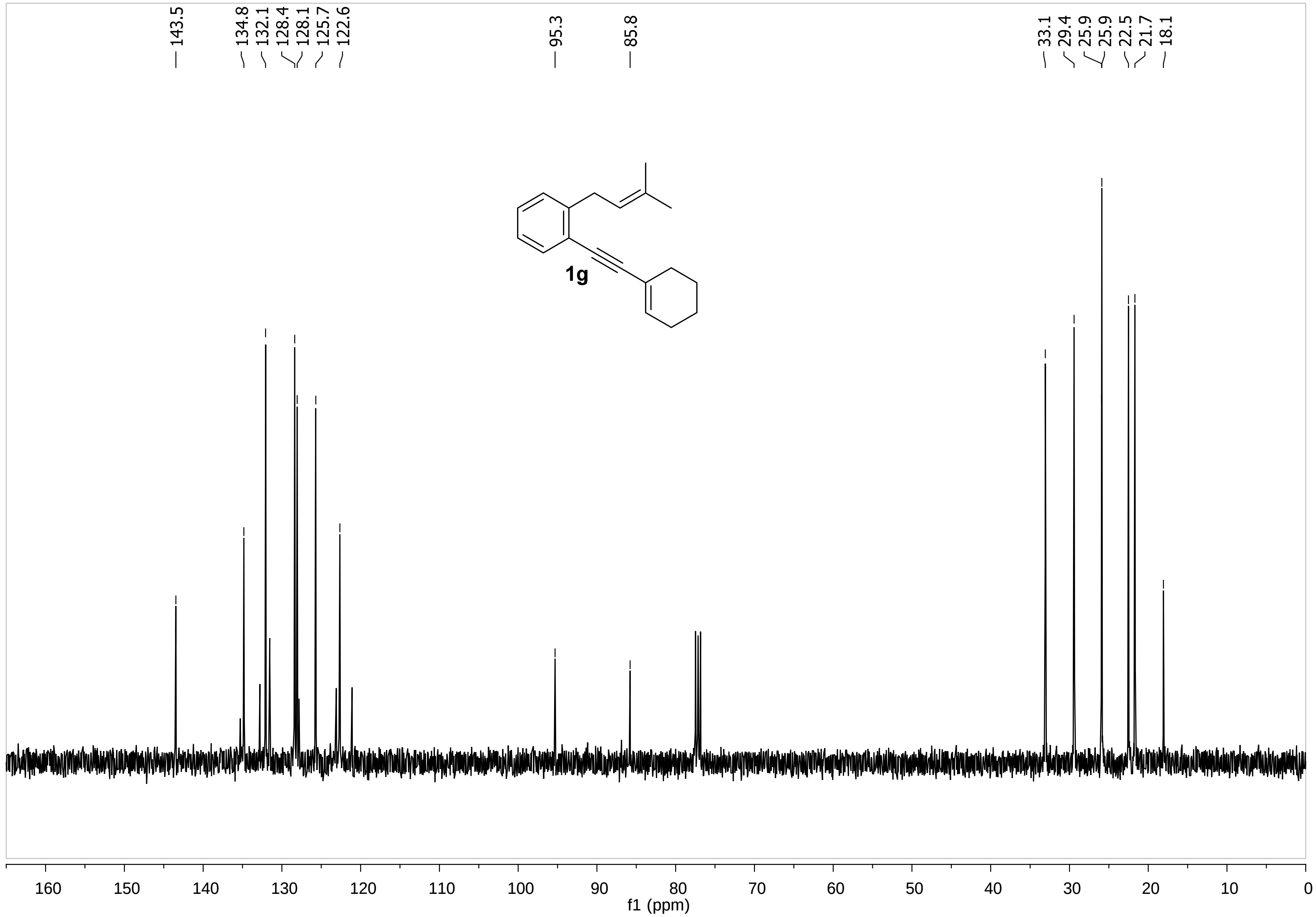
143.5
134.8
132.1
128.4
128.1
125.7
122.6
95.3
85.8
33.1
29.4
25.9
25.9
22.5
21.7
18.1
1g
160
150
140
130
120
110
100
90
80
70
60
50
40
30
20
10
0
f1 (ppm)

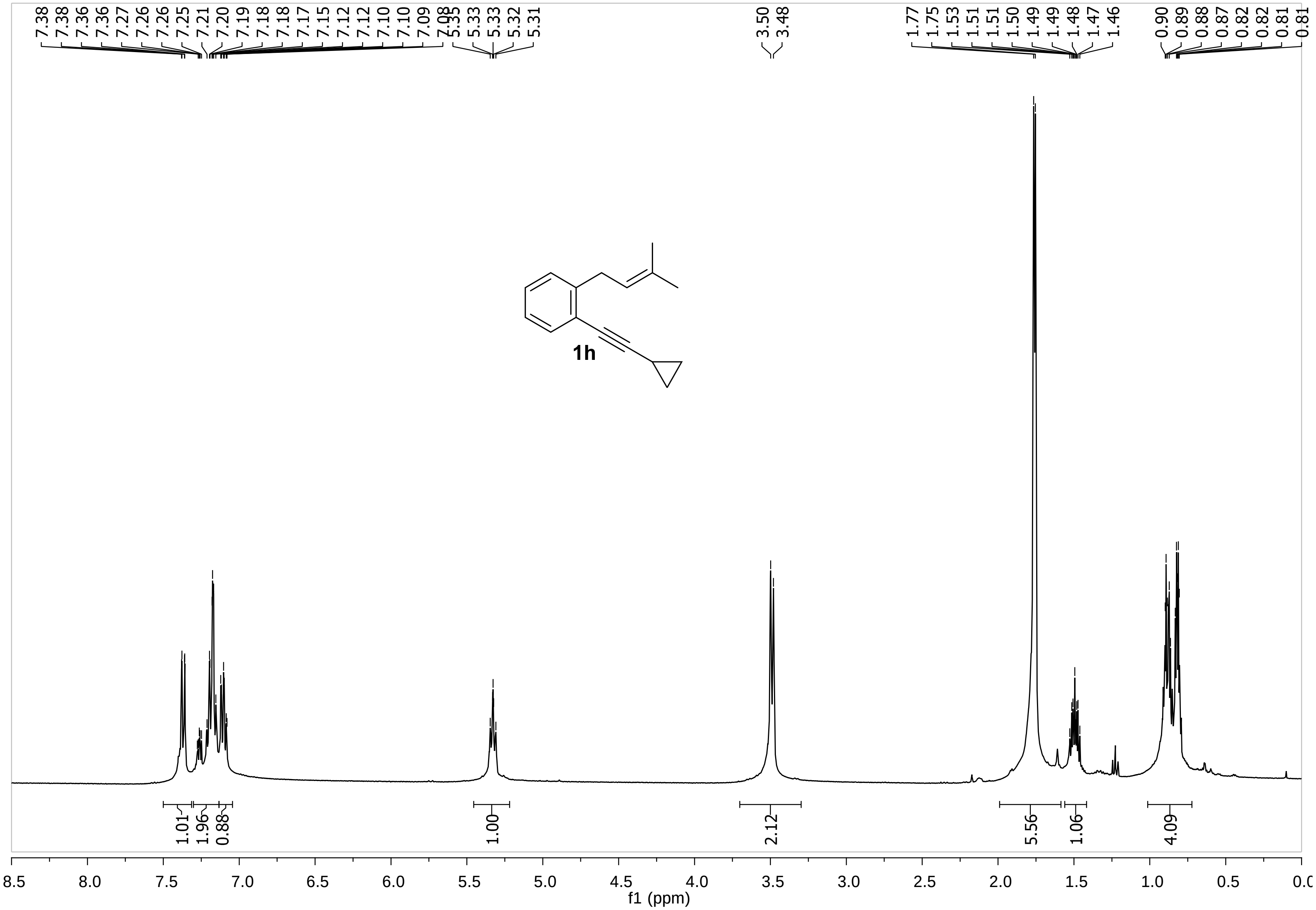
7.38 7.38 7.36 7.36 7.27 7.26 7.26 7.25 7.21 7.20 7.19 7.18 7.18 7.17 7.15 7.12 7.12 7.10 7.10 7.09 7.08 5.35 5.33 5.33 5.32 5.31
3.50 3.48
1.77 1.75 1.53 1.51 1.51 1.50 1.49 1.49 1.48 1.47 1.46
0.90 0.89 0.88 0.87 0.82 0.82 0.81 0.81
1h
1.01 1.96 0.88 1.00 2.12 5.56 1.06 4.09
f1 (ppm)

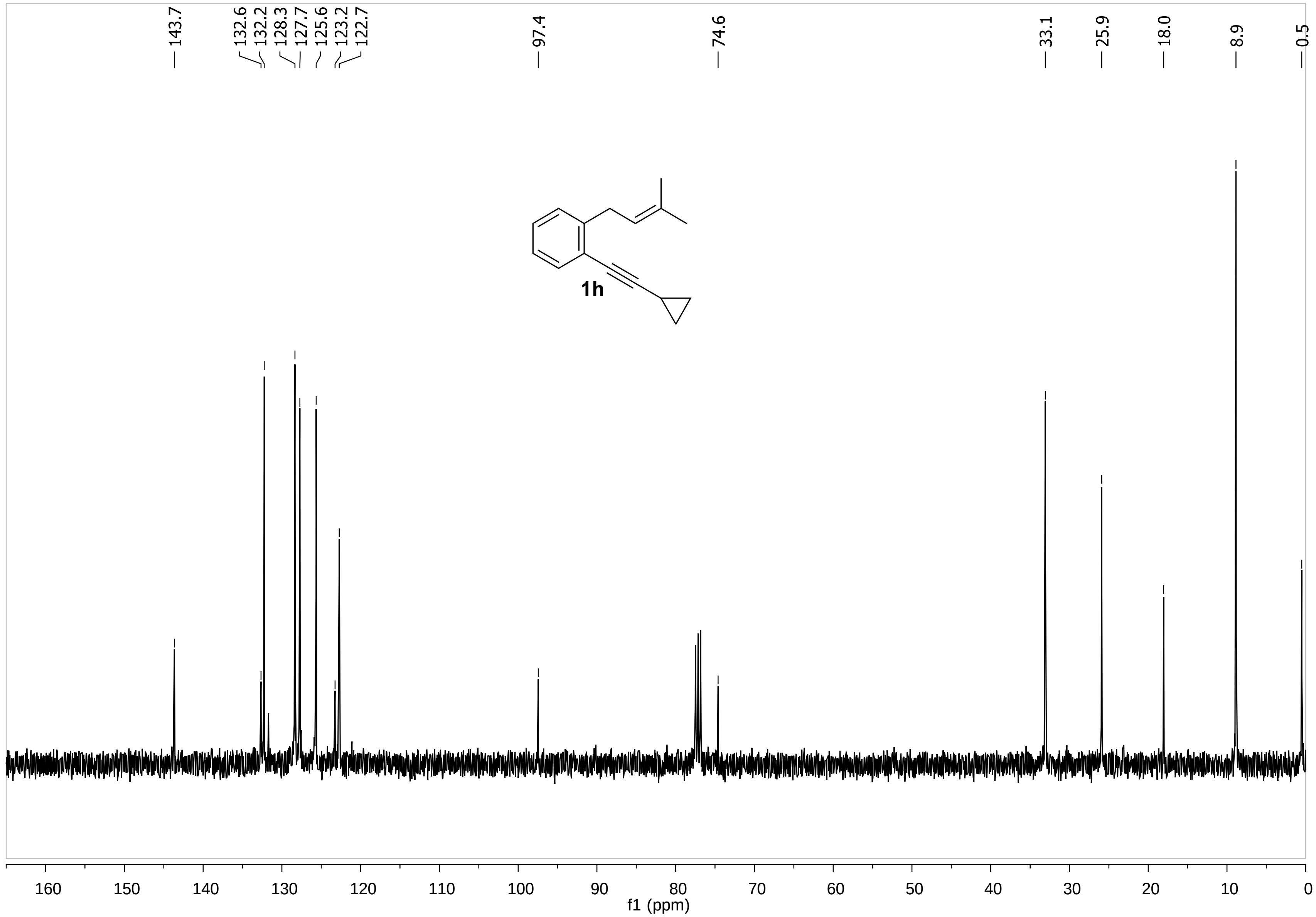
143.7
132.6
132.2
128.3
127.7
125.6
123.2
122.7
97.4
74.6
33.1
25.9
18.0
8.9
0.5
1h
160
150
140
130
120
110
100
90
80
70
60
50
40
30
20
10
0
f1 (ppm)

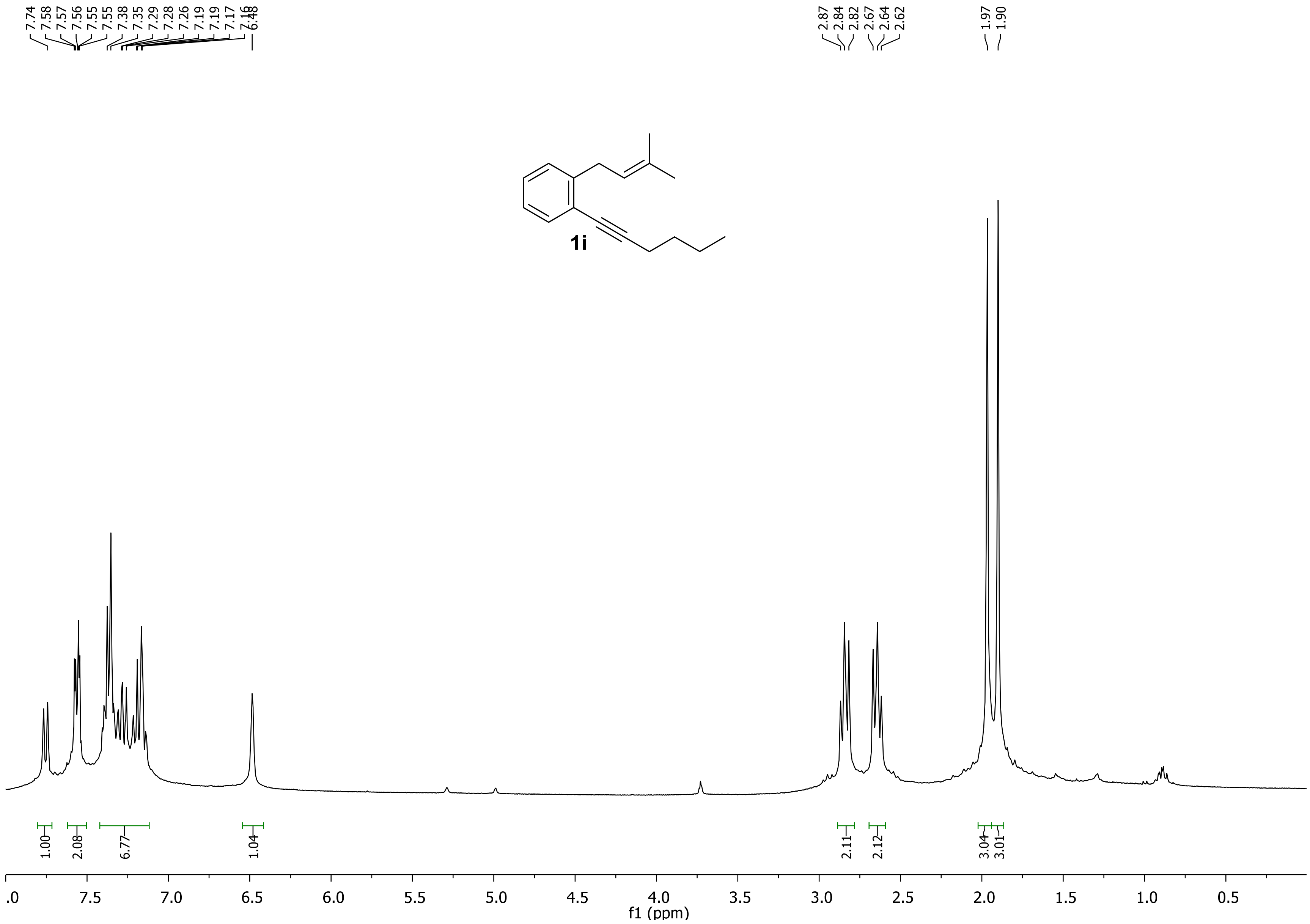
7.74
7.58
7.57
7.56
7.55
7.55
7.38
7.35
7.29
7.28
7.26
7.19
7.19
7.17
7.16
6.48
2.87
2.84
2.82
2.67
2.64
2.62
1.97
1.90
1i
1.00
2.08
6.77
1.04
2.11
2.12
3.04
3.01
.0
7.5
7.0
6.5
6.0
5.5
5.0
4.5
4.0
3.5
3.0
2.5
2.0
1.5
1.0
0.5
f1 (ppm)

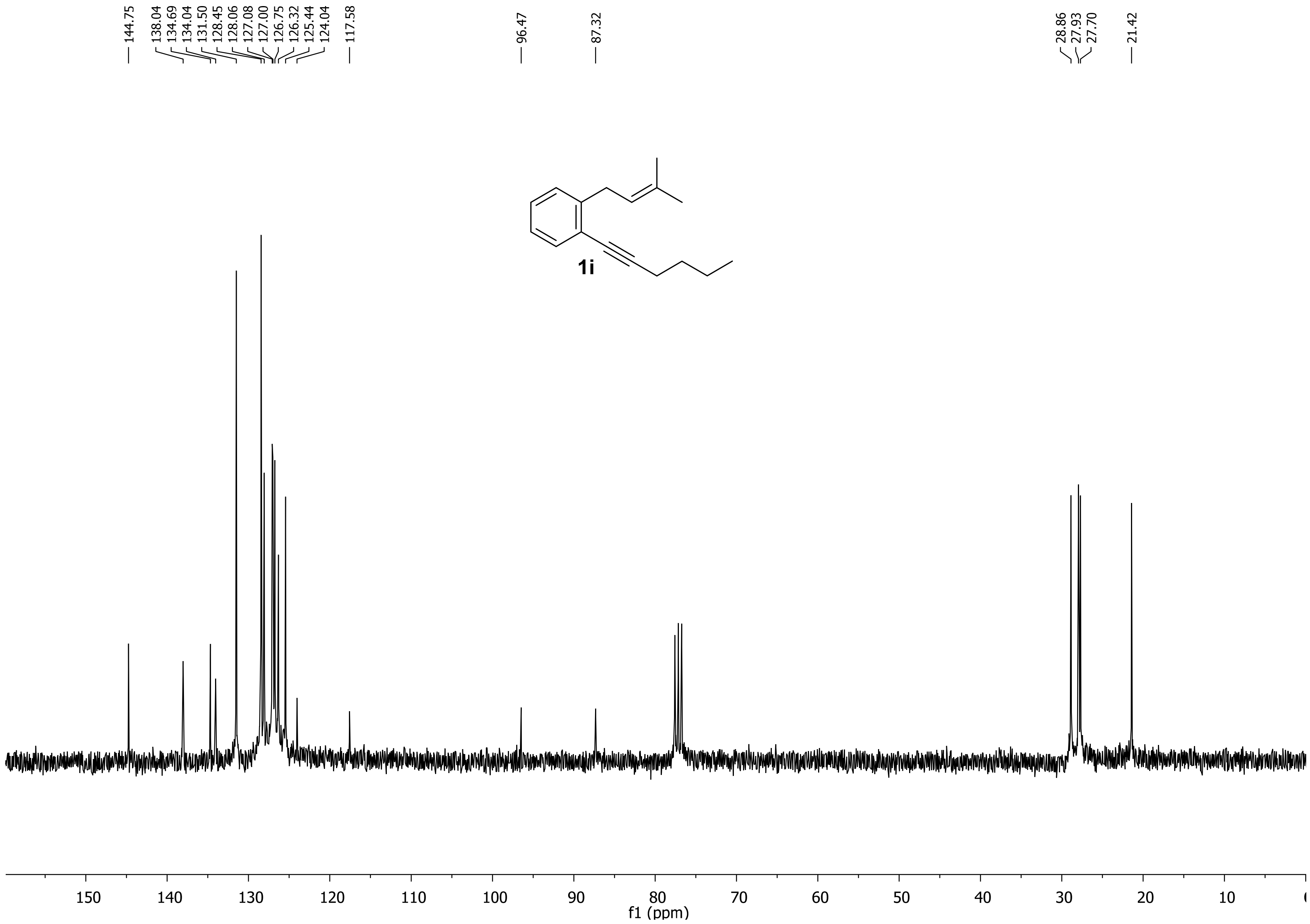
144.75
138.04
134.69
134.04
131.50
128.45
128.06
127.08
127.00
126.75
126.32
125.44
124.04
117.58
96.47
87.32
28.86
27.93
27.70
21.42
1i
150
140
130
120
110
100
90
80
70
60
50
40
30
20
10
f1 (ppm)

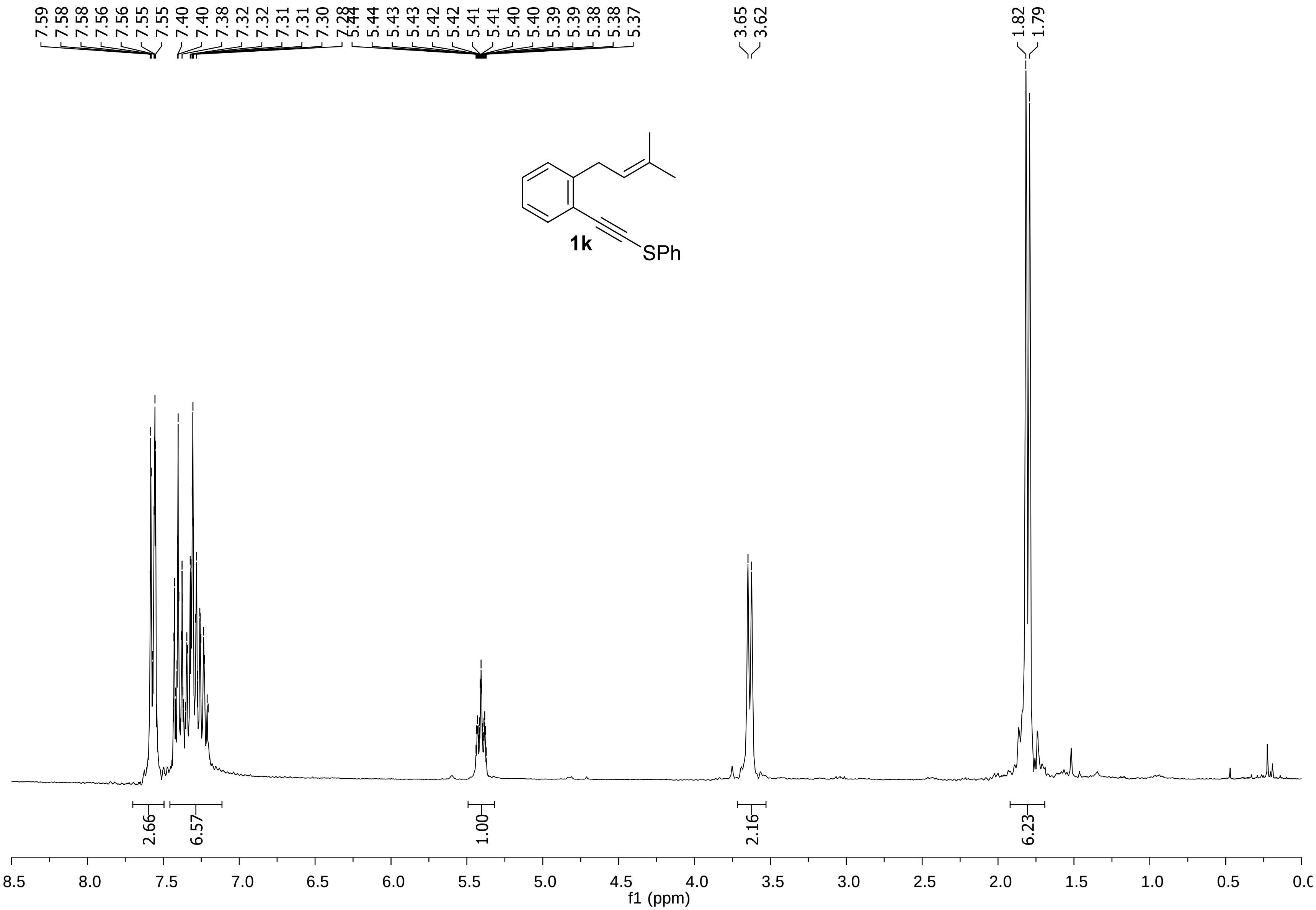
7.59
7.58
7.58
7.56
7.56
7.55
7.55
7.40
7.40
7.38
7.32
7.32
7.31
7.31
7.30
7.28
5.44
5.44
5.43
5.43
5.42
5.42
5.41
5.41
5.40
5.40
5.39
5.39
5.38
5.38
5.37
3.65
3.62
1.82
1.79
1k
SPh
2.66
6.57
1.00
2.16
6.23
8.5
8.0
7.5
7.0
6.5
6.0
5.5
5.0
4.5
4.0
3.5
3.0
2.5
2.0
1.5
1.0
0.5
f1 (ppm)

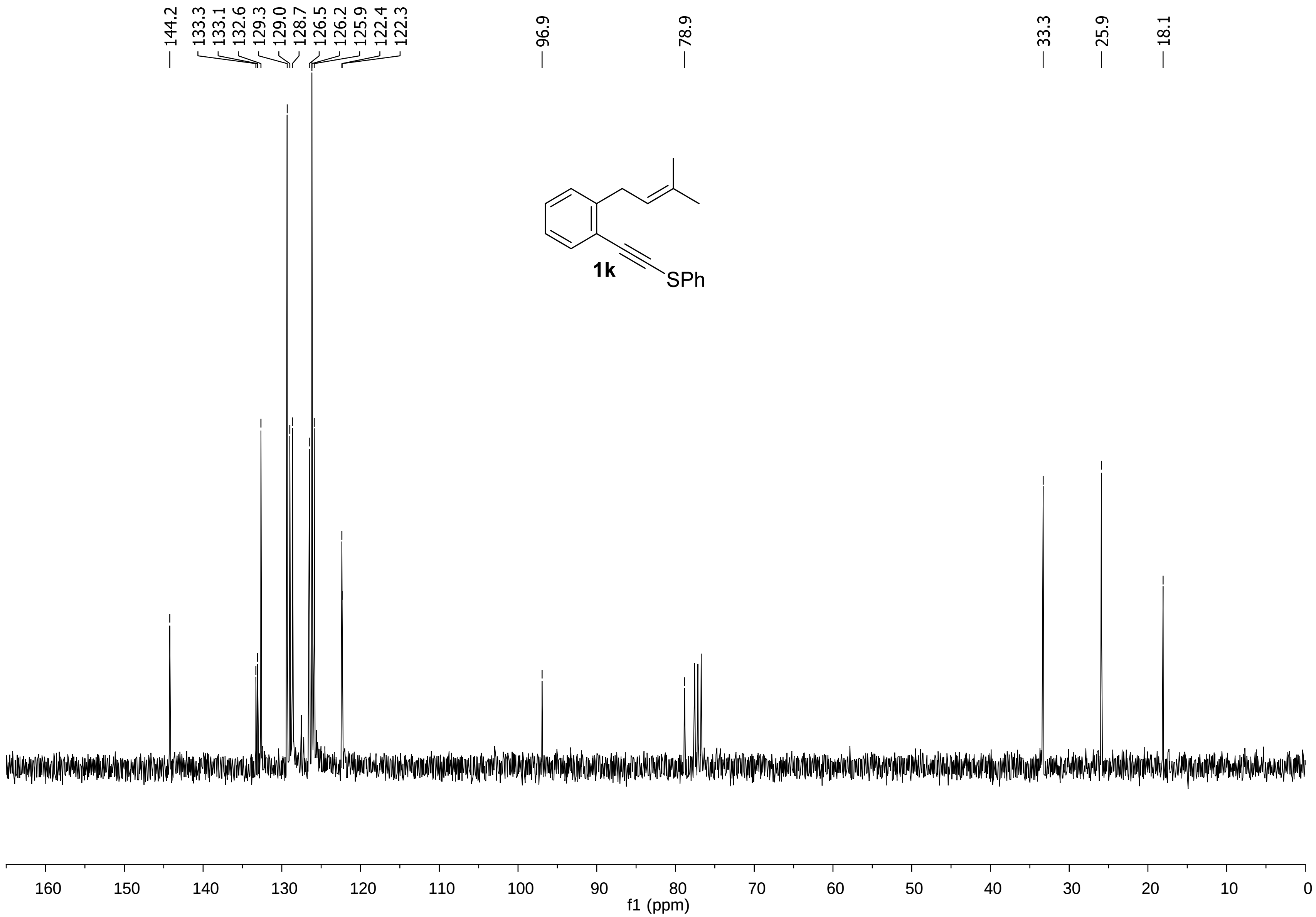
144.2
133.3
133.1
132.6
129.3
129.0
128.7
126.5
126.2
125.9
122.4
122.3
96.9
78.9
33.3
25.9
18.1
1k
SPh
160
150
140
130
120
110
100
90
80
70
60
50
40
30
20
10
0
f1 (ppm)

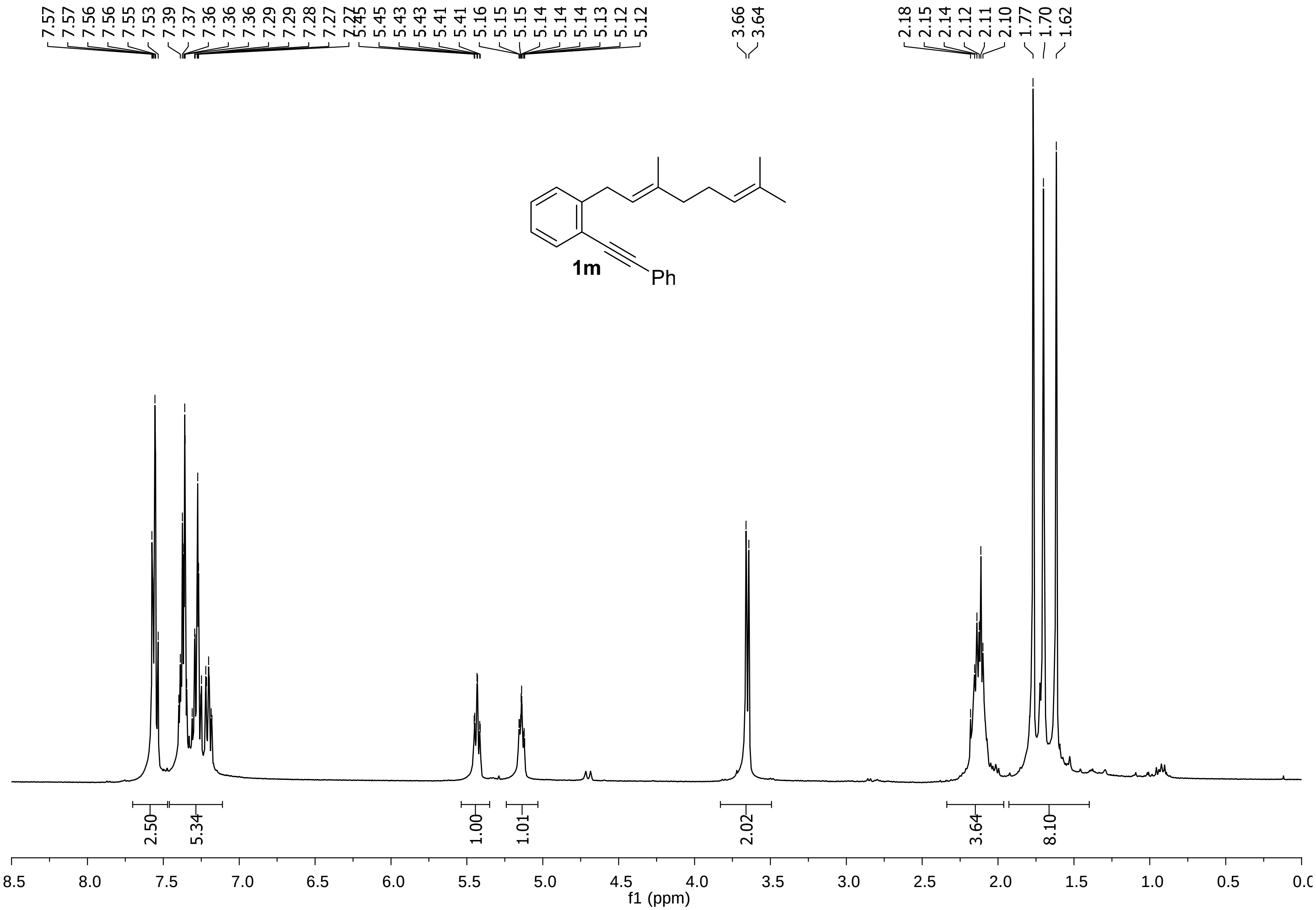
7.57 7.57 7.56 7.56 7.55 7.53 7.39 7.37 7.36 7.36 7.36 7.29 7.29 7.28 7.27 7.27 5.45 5.45 5.43 5.43 5.41 5.41 5.16 5.15 5.15 5.14 5.14 5.14 5.13 5.12 5.12
3.66 3.64
2.18 2.15 2.14 2.12 2.11 2.10 1.77 1.70 1.62
1m
Ph
2.50 5.34 1.00 1.01 2.02 3.64 8.10
8.5 8.0 7.5 7.0 6.5 6.0 5.5 5.0 4.5 4.0 3.5 3.0 2.5 2.0 1.5 1.0 0.5
f1 (ppm)

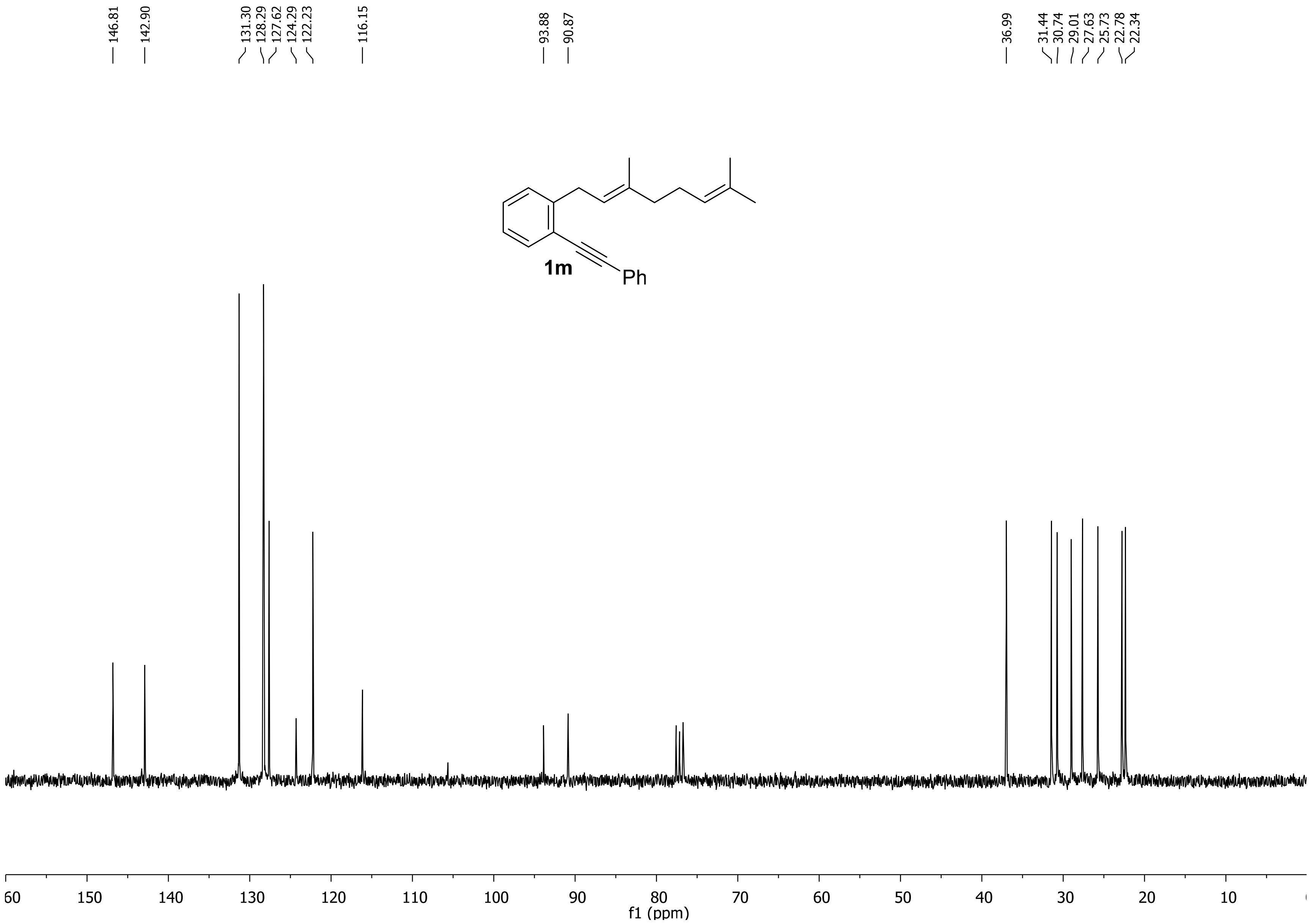

146.81
142.90
131.30
128.29
127.62
124.29
122.23
116.15
93.88
90.87
36.99
31.44
30.74
29.01
27.63
25.73
22.78
22.34
1m
Ph
f1 (ppm)

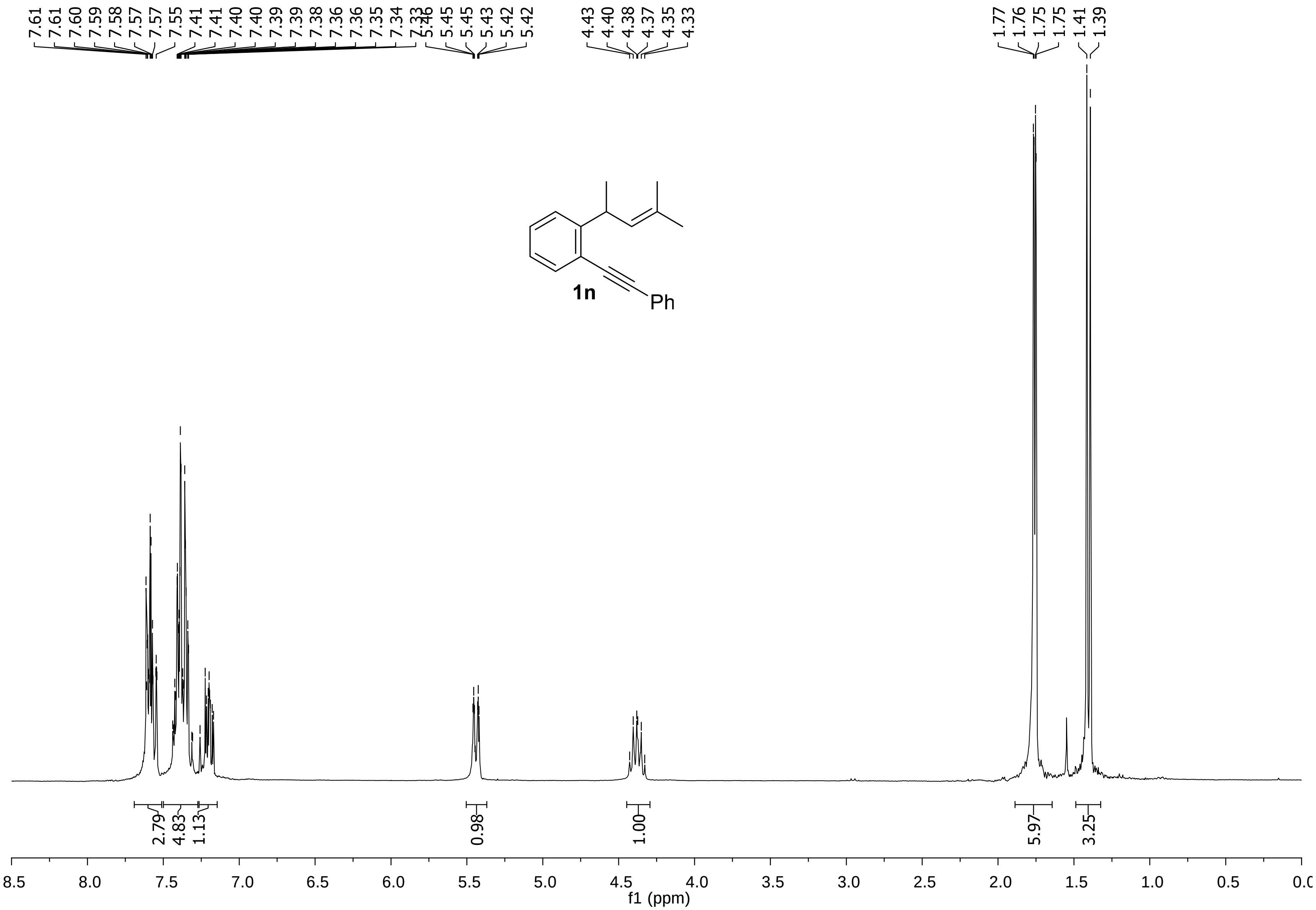
7.61
7.61
7.60
7.59
7.58
7.57
7.57
7.55
7.41
7.41
7.40
7.40
7.39
7.39
7.38
7.36
7.36
7.35
7.34
7.33
5.46
5.45
5.45
5.43
5.42
5.42
4.43
4.40
4.38
4.37
4.35
4.33
1.77
1.76
1.75
1.75
1.41
1.39
1n
Ph
2.79
4.83
1.13
0.98
1.00
5.97
3.25
f1 (ppm)

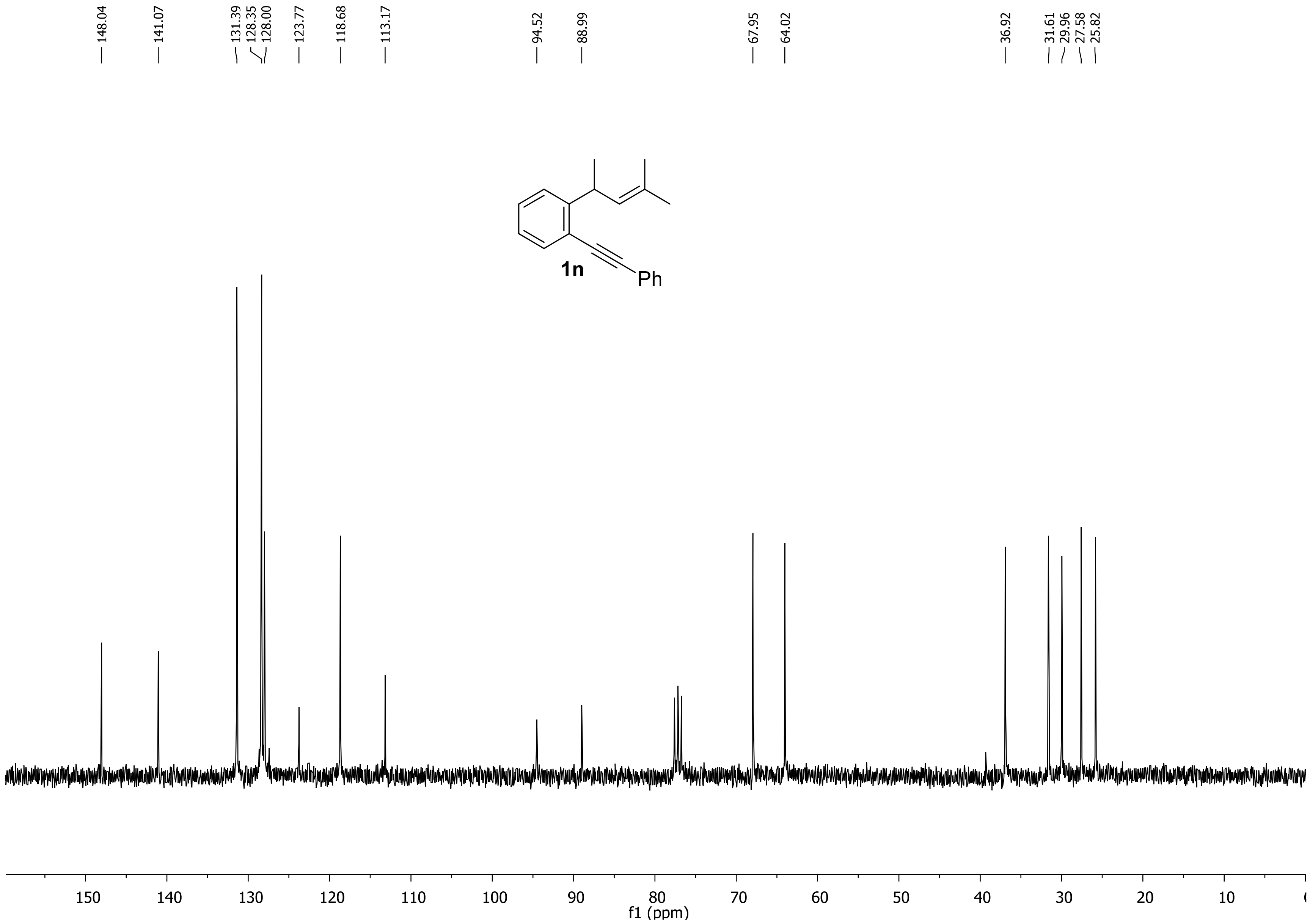
148.04
141.07
131.39
128.35
128.00
123.77
118.68
113.17
94.52
88.99
67.95
64.02
36.92
31.61
29.96
27.58
25.82
1n
Ph
150
140
130
120
110
100
90
80
70
60
50
40
30
20
10
f1 (ppm)

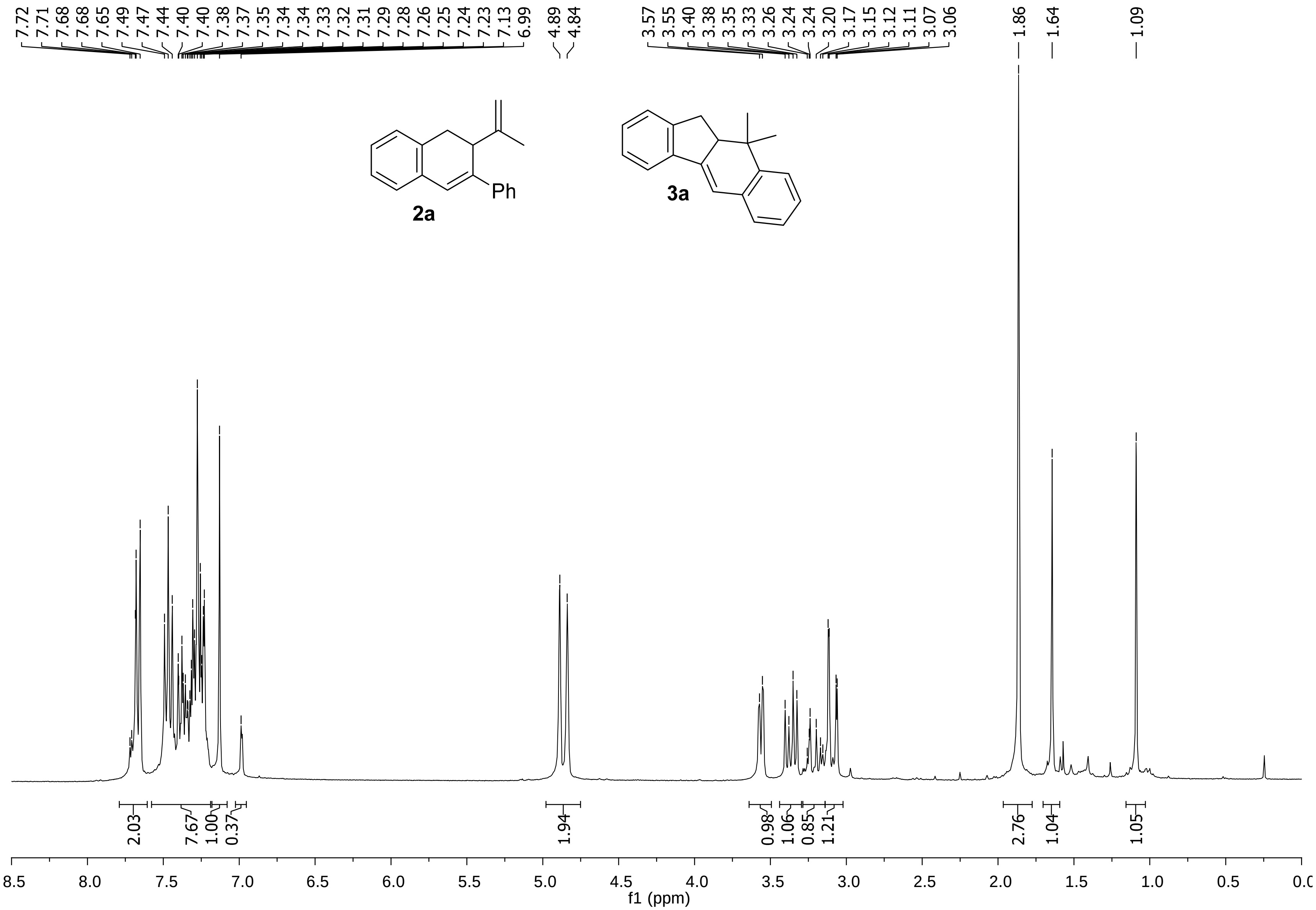
7.72 7.71 7.68 7.68 7.65 7.49 7.47 7.44 7.40 7.40 7.38 7.37 7.35 7.34 7.34 7.33 7.32 7.31 7.29 7.28 7.26 7.25 7.24 7.23 7.13 6.99
4.89 4.84
3.57 3.55 3.40 3.38 3.35 3.33 3.26 3.24 3.24 3.20 3.17 3.15 3.12 3.11 3.07 3.06
1.86
1.64
1.09
Ph
2a
3a
2.03 7.67 1.00 0.37 1.94 0.98 1.06 0.85 1.21 2.76 1.04 1.05
f1 (ppm)

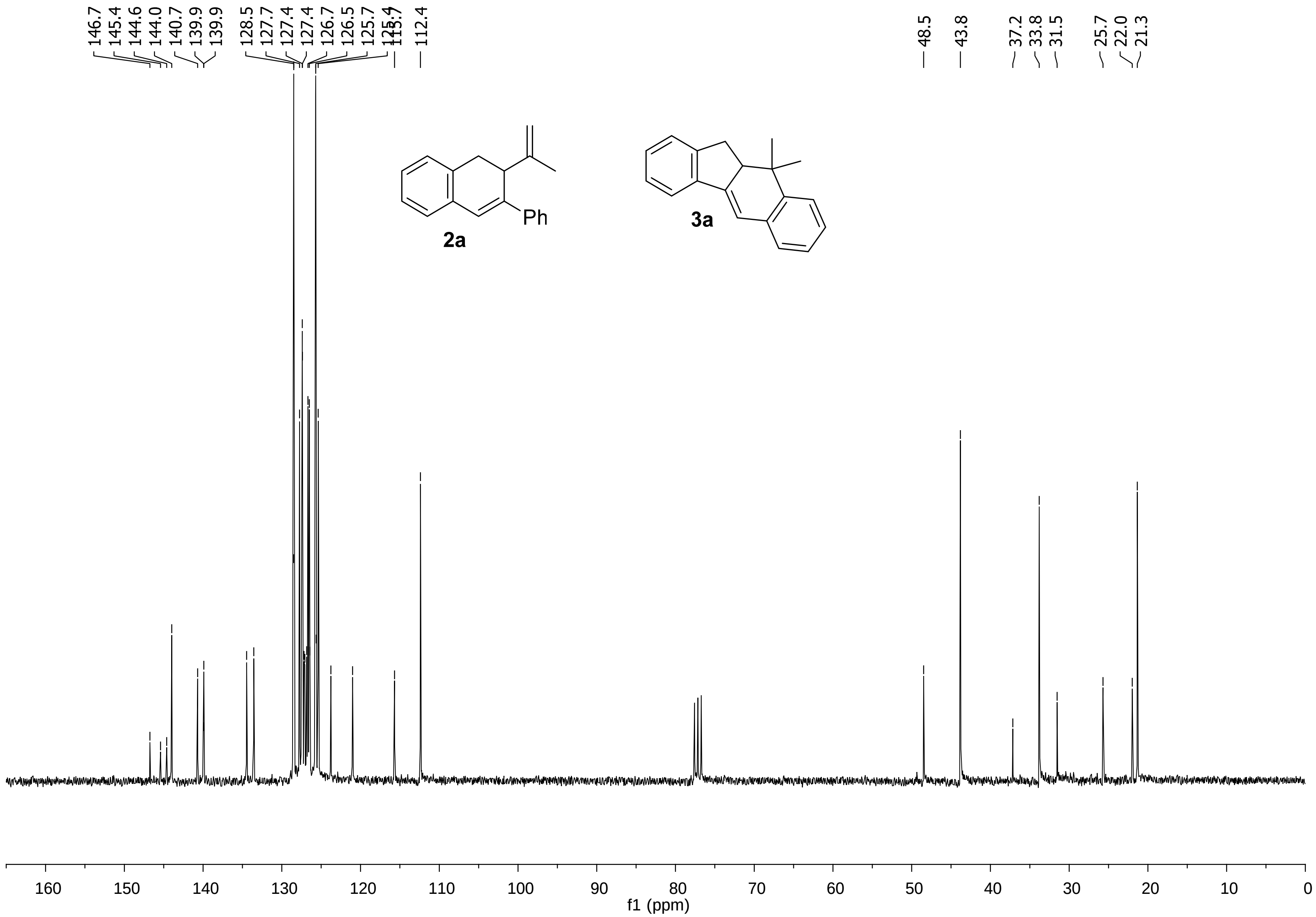
146.7
145.4
144.6
144.0
140.7
139.9
139.9
128.5
127.7
127.4
127.4
126.7
126.5
125.7
125.4
115.7
112.4
48.5
43.8
37.2
33.8
31.5
25.7
22.0
21.3
Ph
2a
3a
160
150
140
130
120
110
100
90
80
70
60
50
40
30
20
10
0
f1 (ppm)

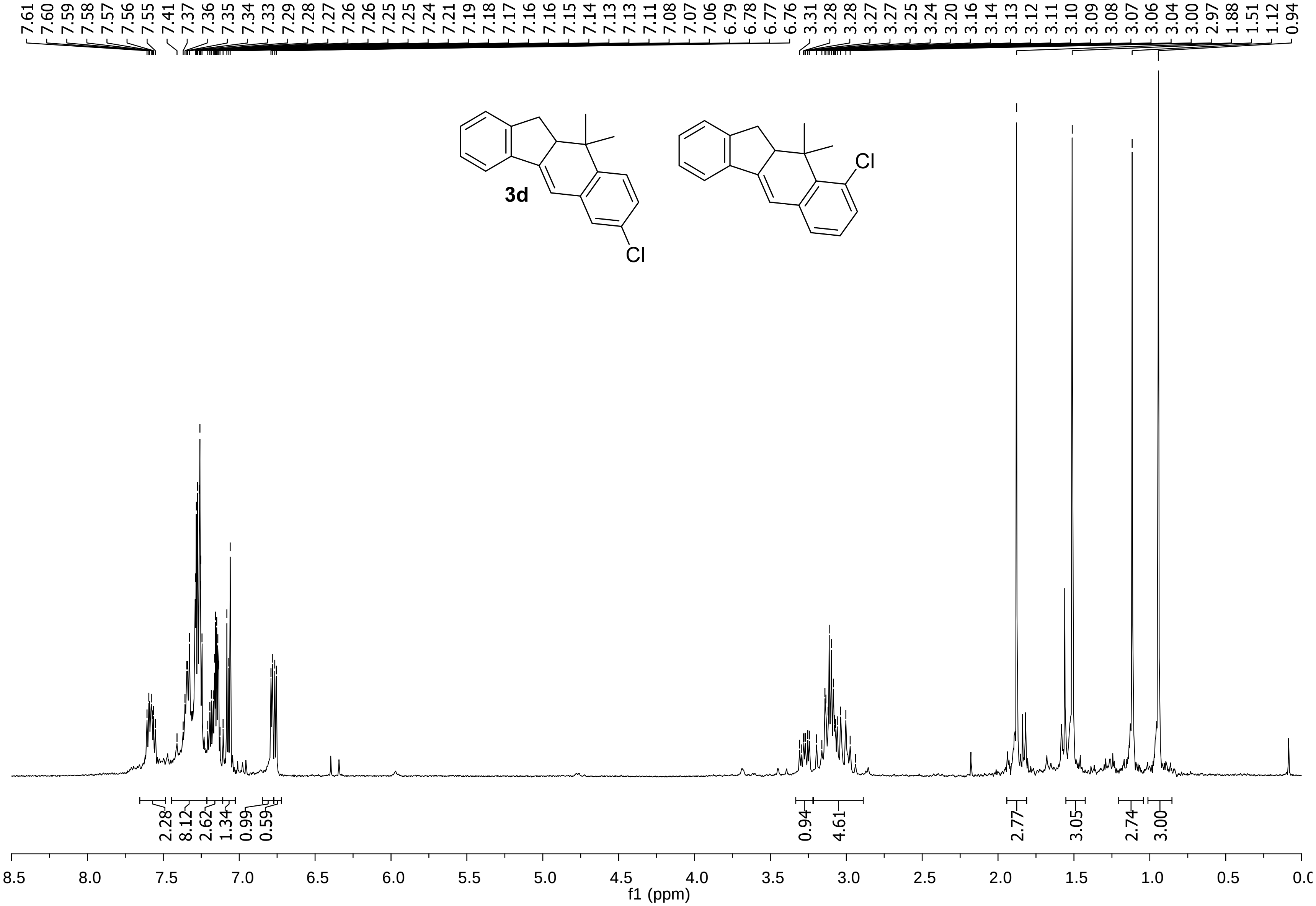
7.61 7.60 7.59 7.58 7.57 7.56 7.55 7.41 7.37 7.36 7.35 7.34 7.33 7.29 7.28 7.27 7.26 7.26 7.25 7.25 7.24 7.21 7.19 7.18 7.17 7.16 7.16 7.15 7.14 7.13 7.13 7.11 7.08 7.07 7.06 6.79 6.78 6.77 6.76 3.31 3.28 3.28 3.27 3.27 3.25 3.24 3.20 3.16 3.14 3.13 3.12 3.11 3.10 3.09 3.08 3.07 3.06 3.04 3.00 2.97 1.88 1.51 1.12 0.94
3d
Cl
Cl
2.28
8.12
2.62
1.34
0.99
0.59
0.94
4.61
2.77
3.05
2.74
3.00
8.5 8.0 7.5 7.0 6.5 6.0 5.5 5.0 4.5 4.0 3.5 3.0 2.5 2.0 1.5 1.0 0.5
f1 (ppm)

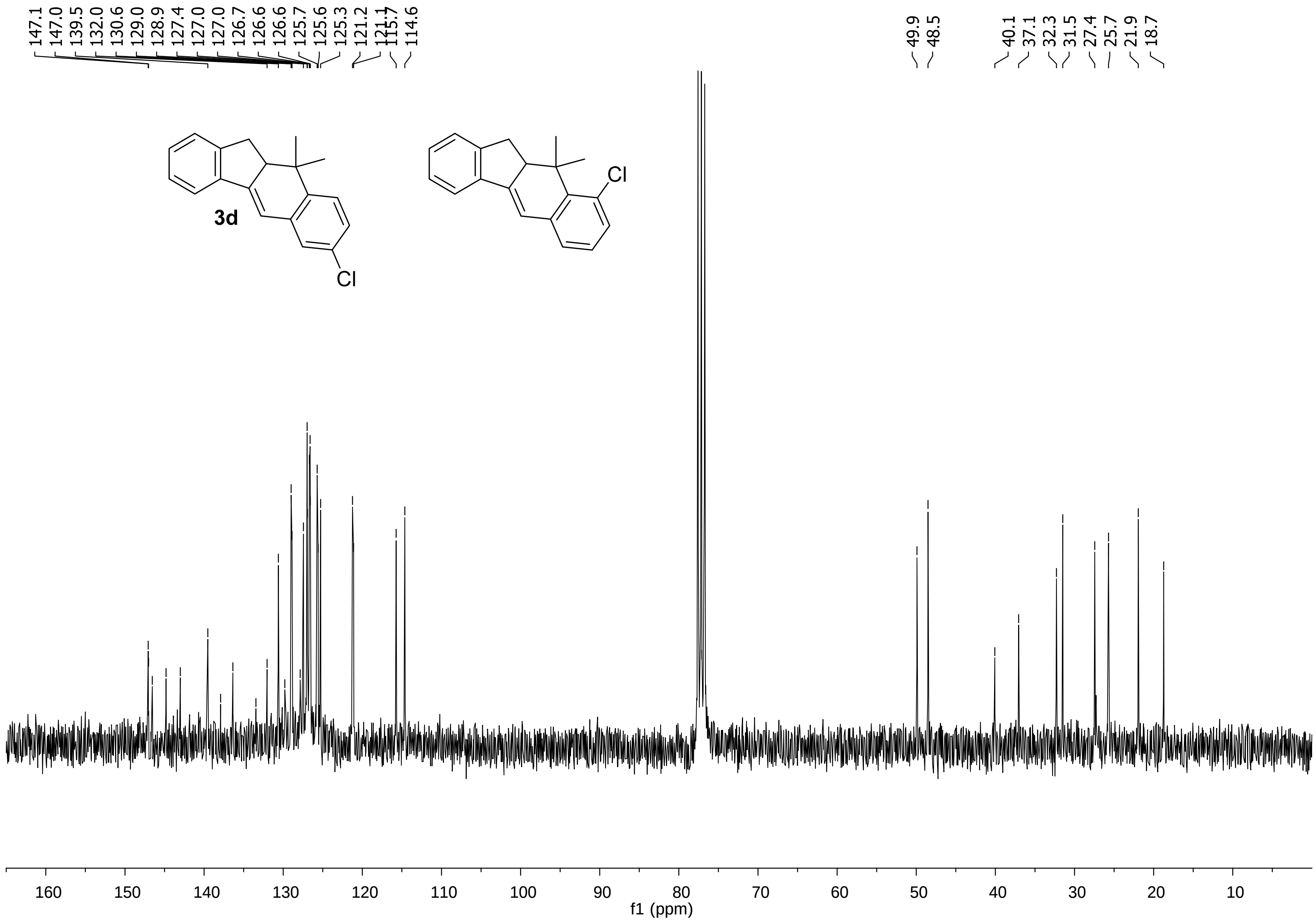
147.1
147.0
139.5
132.0
130.6
129.0
128.9
127.4
127.0
127.0
126.7
126.6
126.6
125.7
125.6
125.3
121.2
121.1
115.7
114.6
49.9
48.5
40.1
37.1
32.3
31.5
27.4
25.7
21.9
18.7
3d
Cl
Cl
160
150
140
130
120
110
100
90
80
70
60
50
40
30
20
10
f1 (ppm)

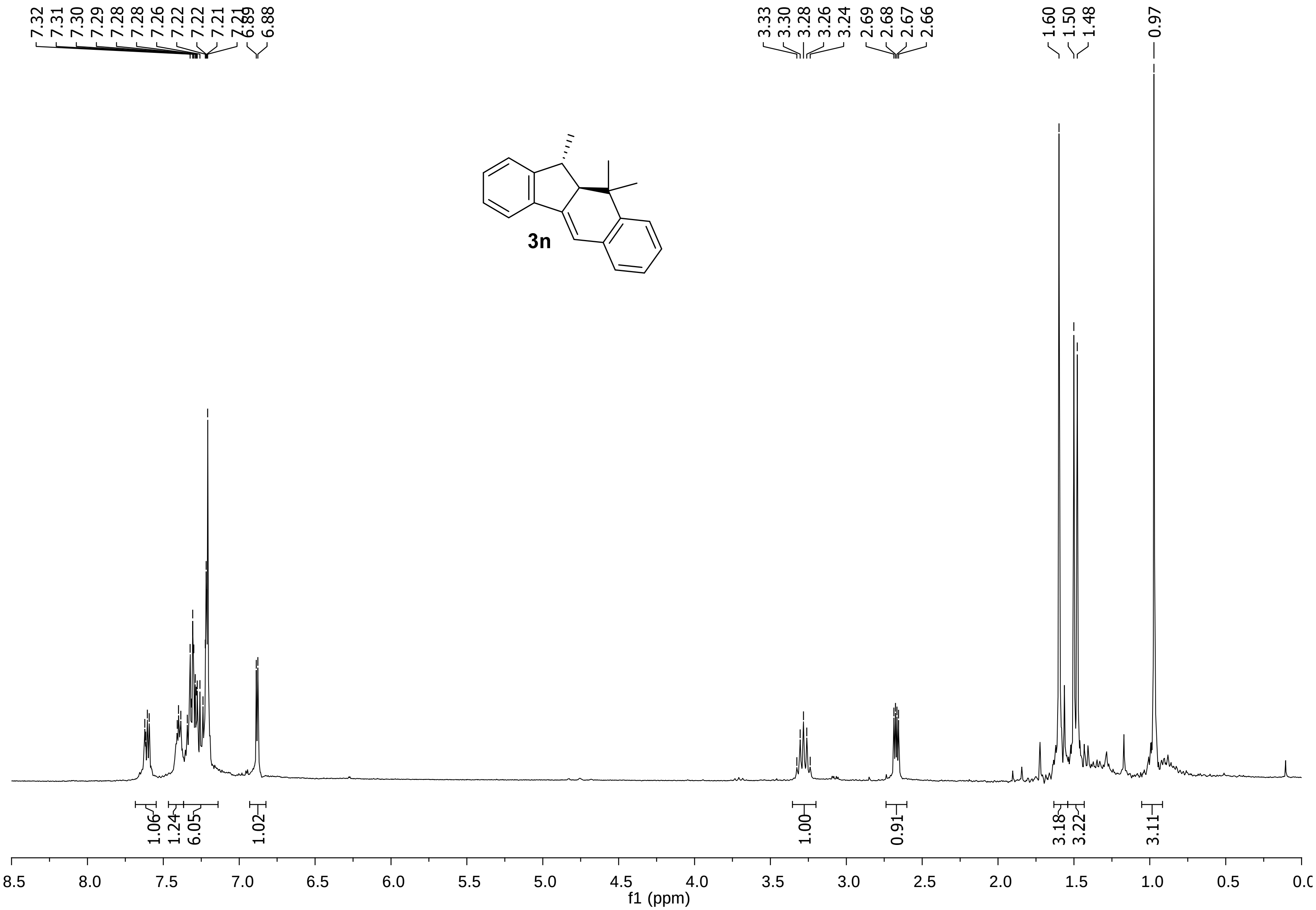

7.32 7.31 7.30 7.29 7.28 7.28 7.26 7.22 7.22 7.21 7.21 6.89 6.88
3.33 3.30 3.28 3.26 3.24 2.69 2.68 2.67 2.66
1.60 1.50 1.48
0.97
3n
1.06 1.24 6.05 1.02 1.00 0.91 3.18 3.22 3.11
8.5 8.0 7.5 7.0 6.5 6.0 5.5 5.0 4.5 4.0 3.5 3.0 2.5 2.0 1.5 1.0 0.5 0.0
f1 (ppm)

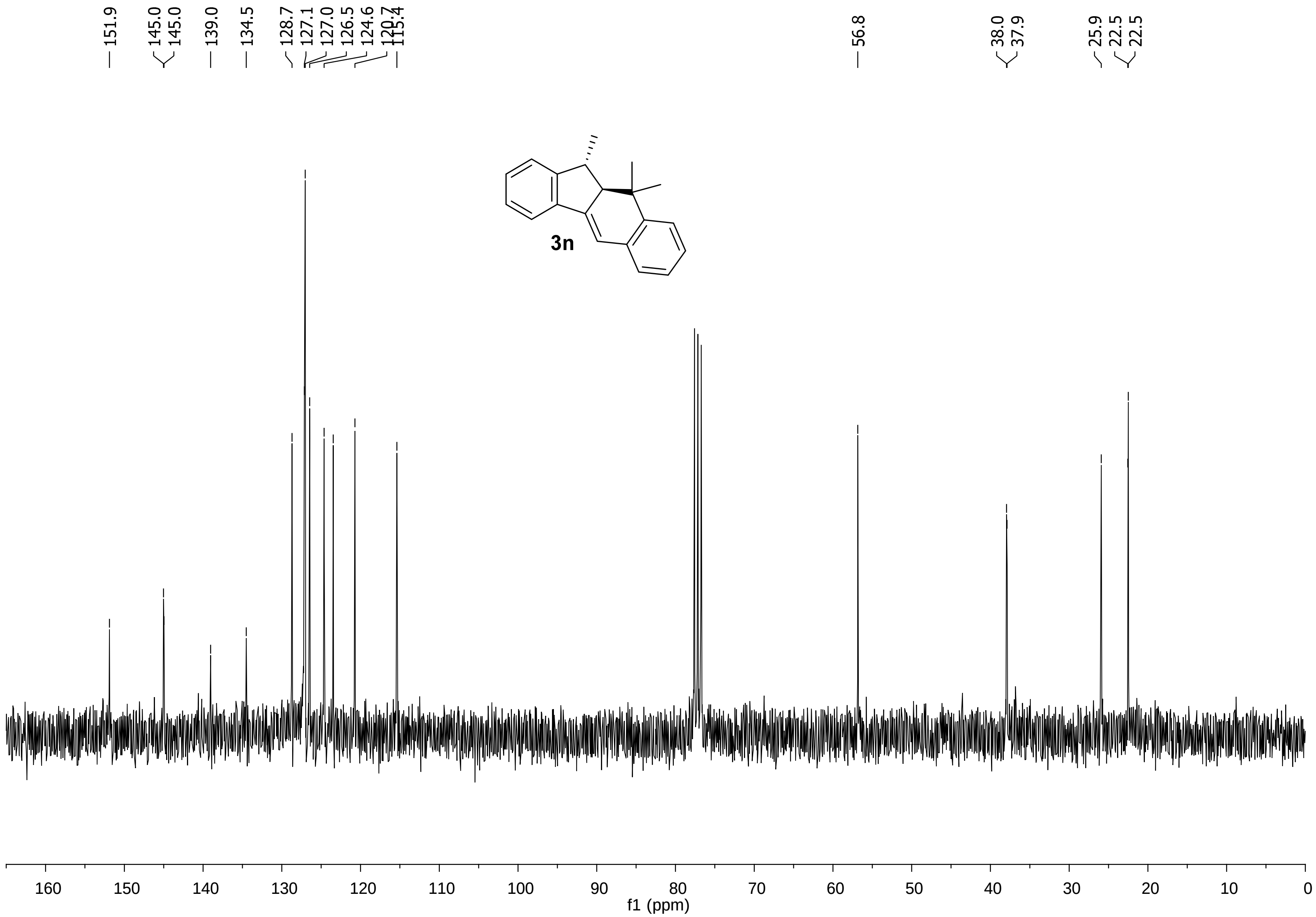
151.9
145.0
145.0
139.0
134.5
128.7
127.1
127.0
126.5
124.6
120.7
115.4
56.8
38.0
37.9
25.9
22.5
22.5
3n
160 150 140 130 120 110 100 90 80 70 60 50 40 30 20 10 0
f1 (ppm)

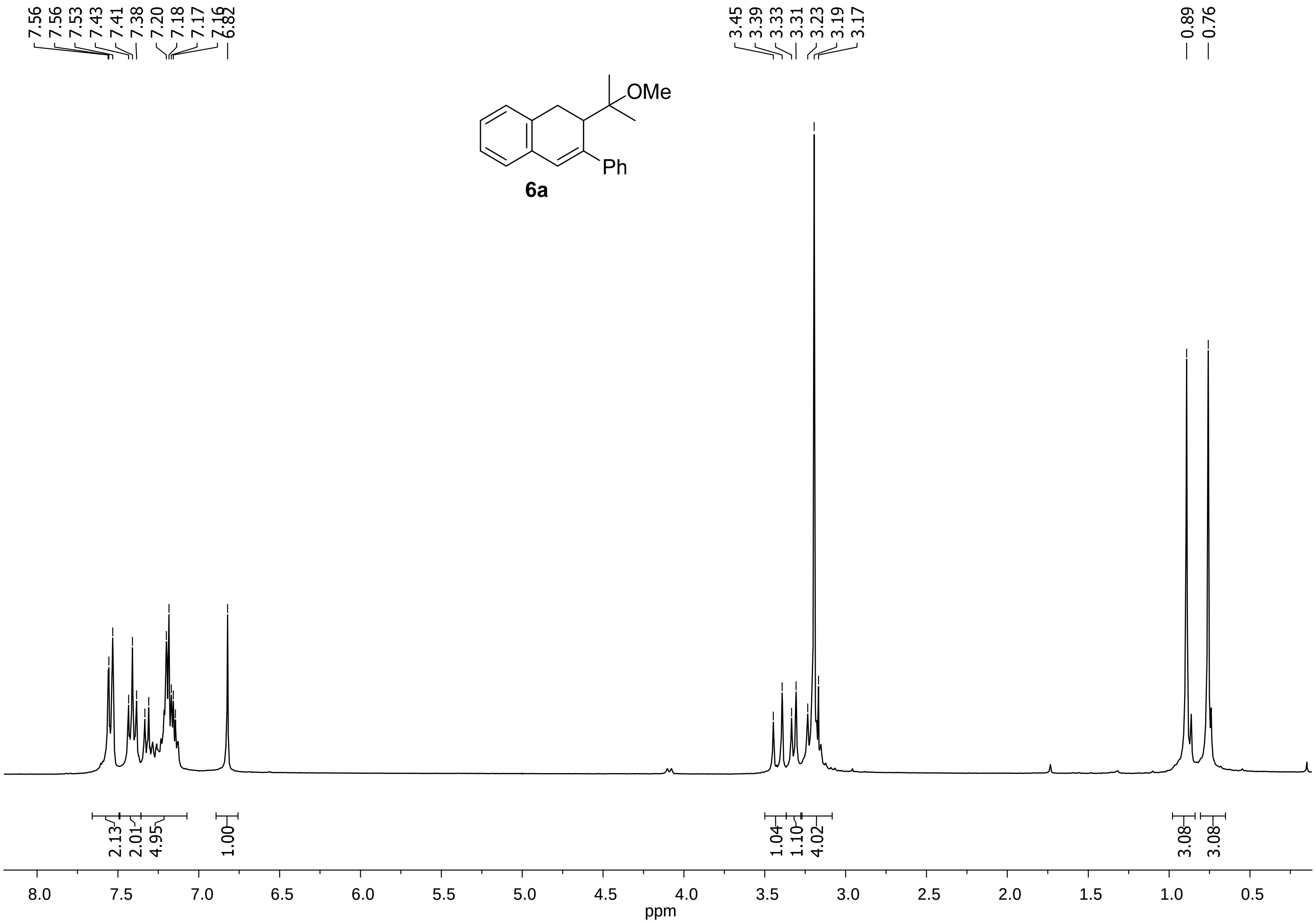
7.56
7.56
7.53
7.43
7.41
7.38
7.20
7.18
7.17
7.16
6.82
3.45
3.39
3.33
3.31
3.23
3.19
3.17
0.89
0.76
OMe
Ph
6a
2.13
2.01
4.95
1.00
1.04
1.10
4.02
3.08
3.08
8.0 7.5 7.0 6.5 6.0 5.5 5.0 4.5 4.0 3.5 3.0 2.5 2.0 1.5 1.0 0.5
ppm

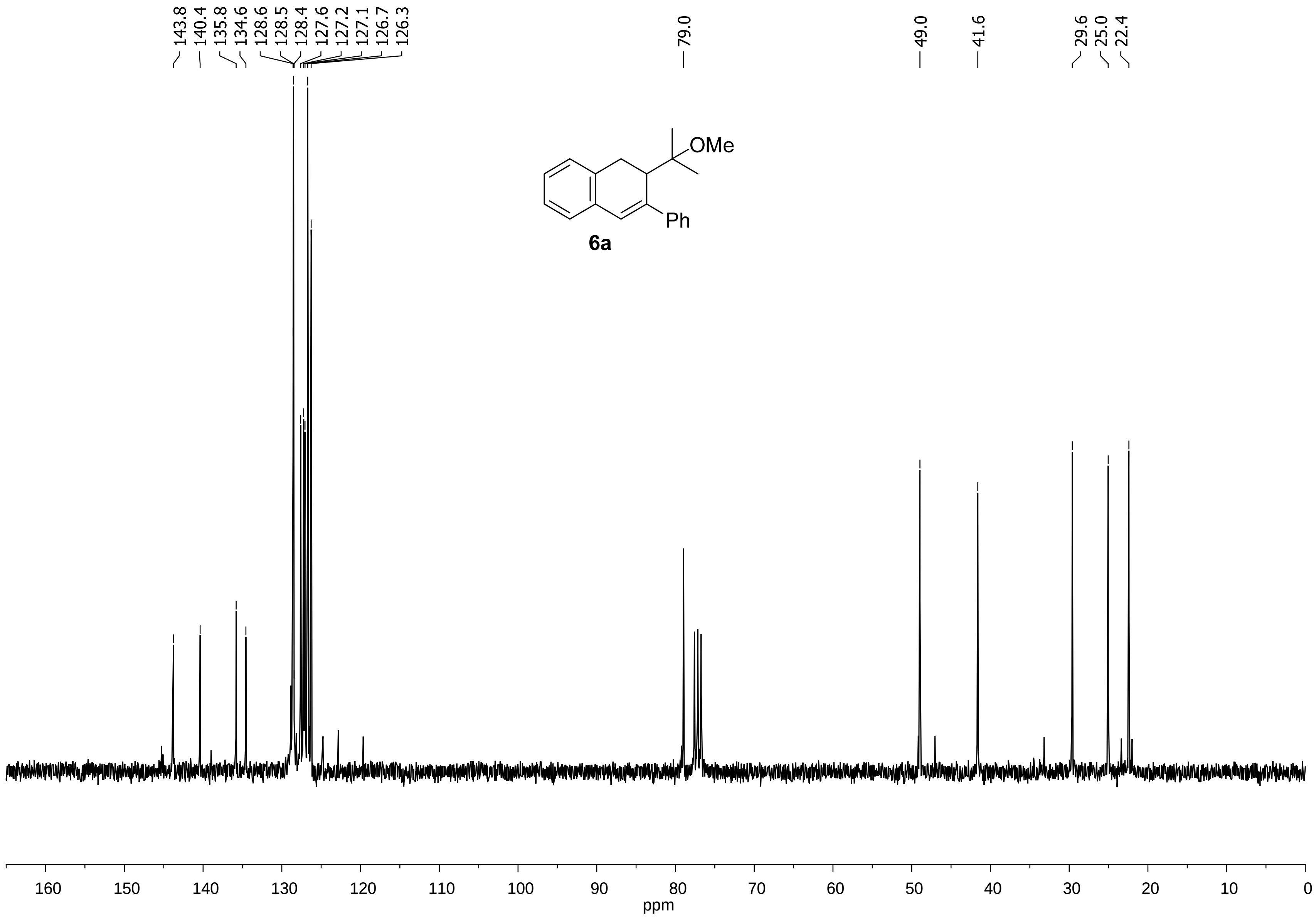
143.8
140.4
135.8
134.6
128.6
128.5
128.4
127.6
127.2
127.1
126.7
126.3
79.0
49.0
41.6
29.6
25.0
22.4
OMe
Ph
6a
160 150 140 130 120 110 100 90 80 70 60 50 40 30 20 10 0
ppm

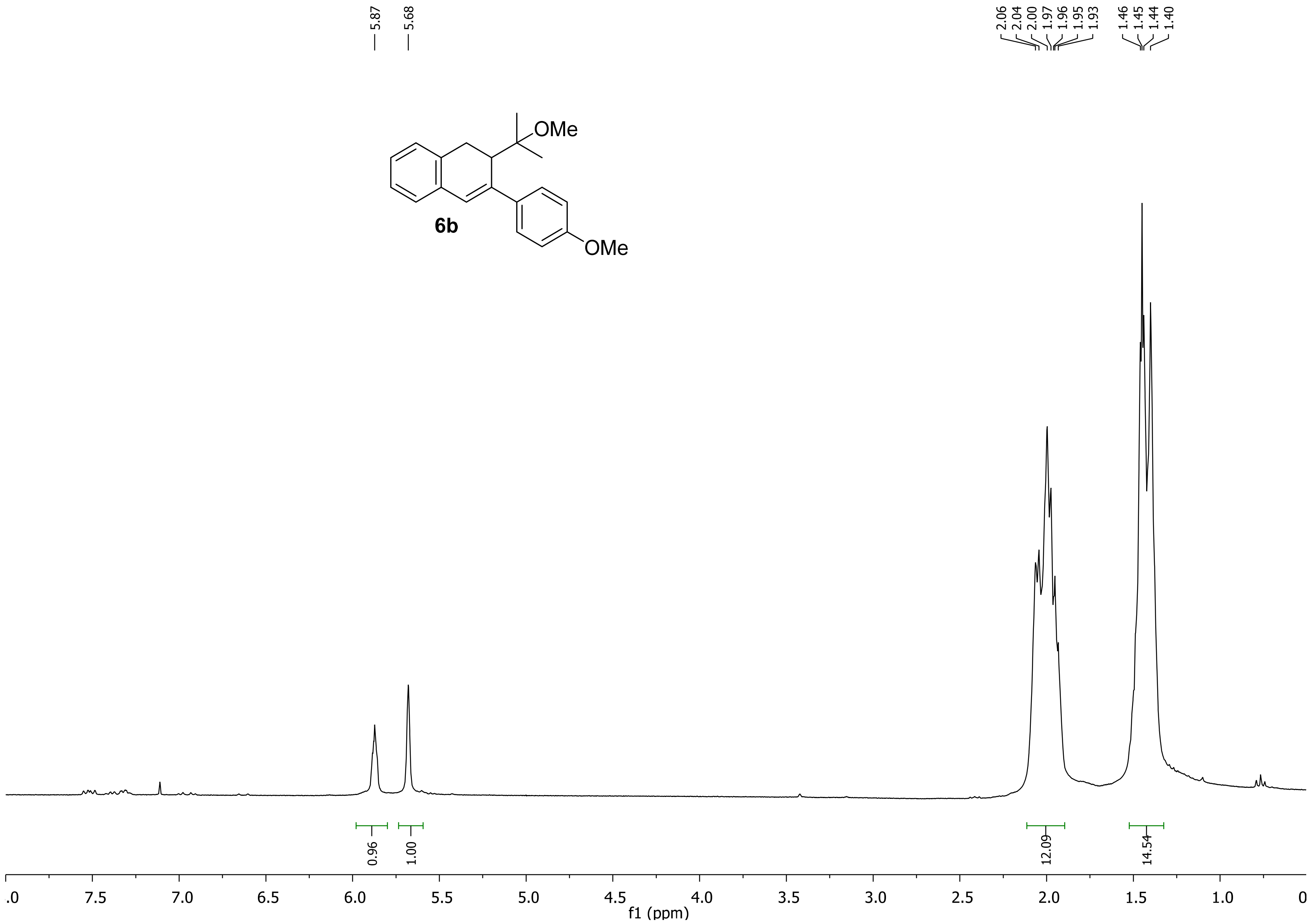
5.87
5.68
2.06
2.04
2.00
1.97
1.96
1.95
1.93
1.46
1.45
1.44
1.40
OMe
OMe
6b
0.96
1.00
12.09
14.54
.0
7.5
7.0
6.5
6.0
5.5
5.0
4.5
4.0
3.5
3.0
2.5
2.0
1.5
1.0
0
f1 (ppm)

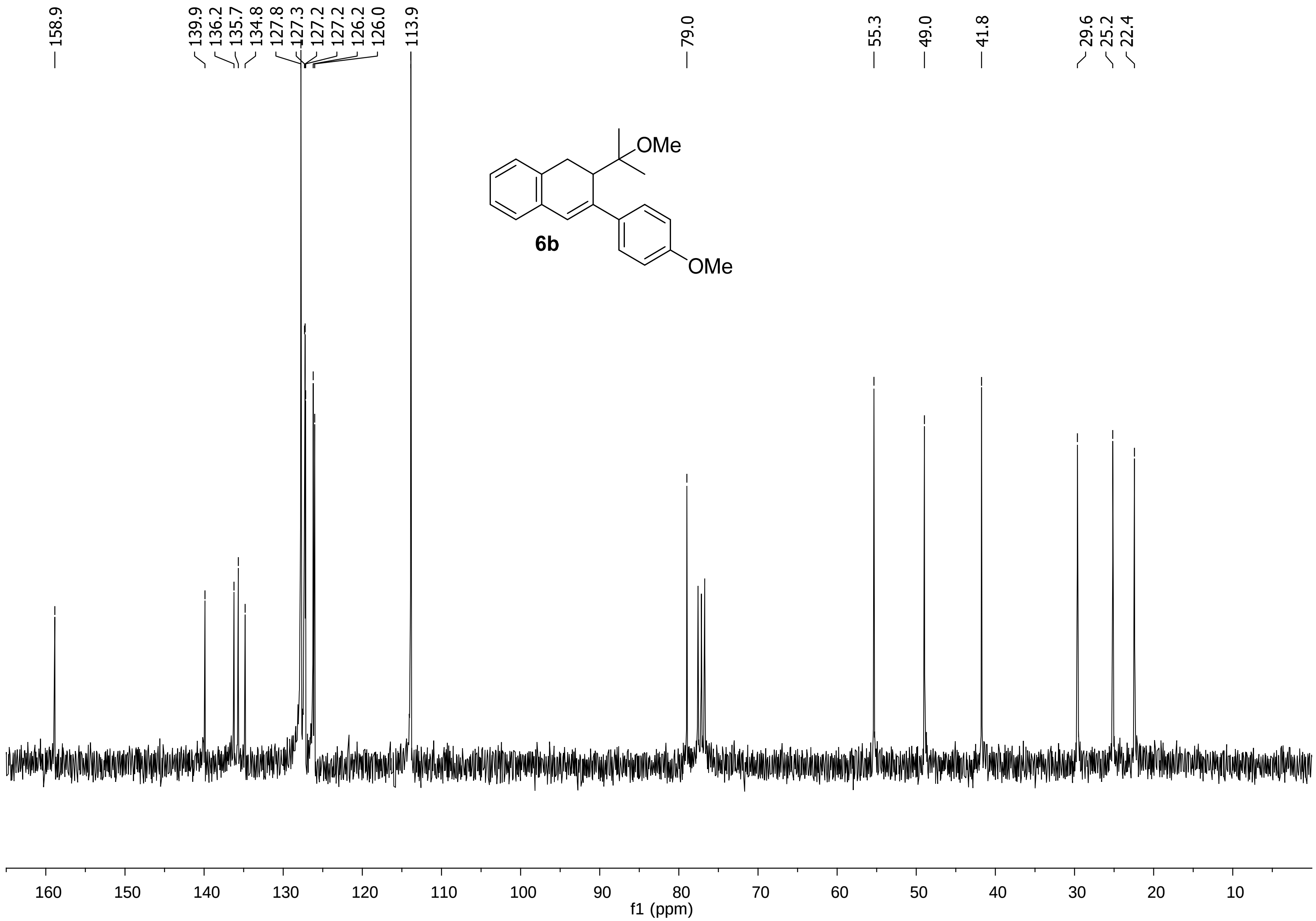
158.9
139.9
136.2
135.7
134.8
127.8
127.3
127.2
127.2
126.2
126.0
113.9
79.0
55.3
49.0
41.8
29.6
25.2
22.4
OMe
6b
OMe
160
150
140
130
120
110
100
90
80
70
60
50
40
30
20
10
f1 (ppm)

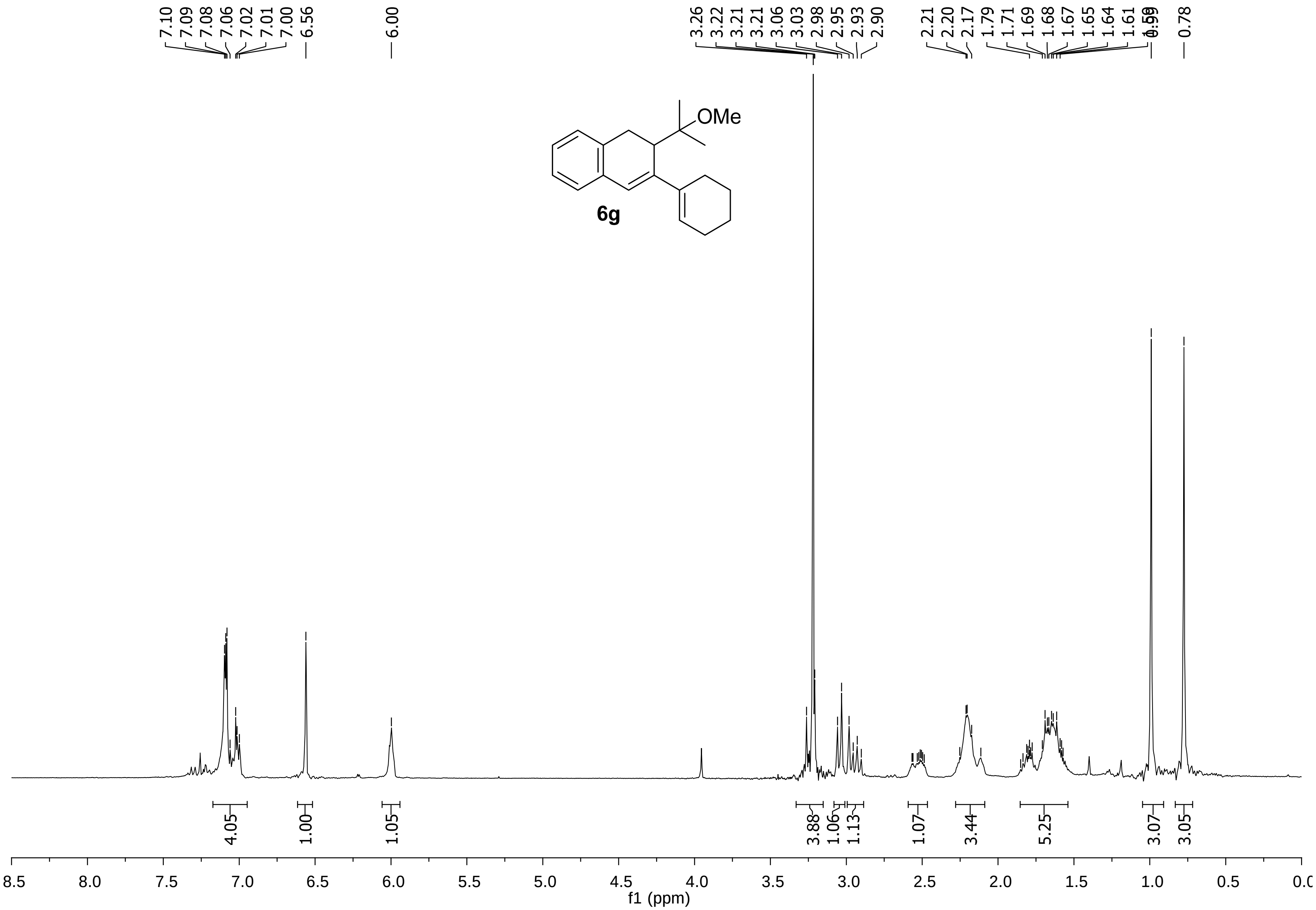
OMe
6g
f1 (ppm)

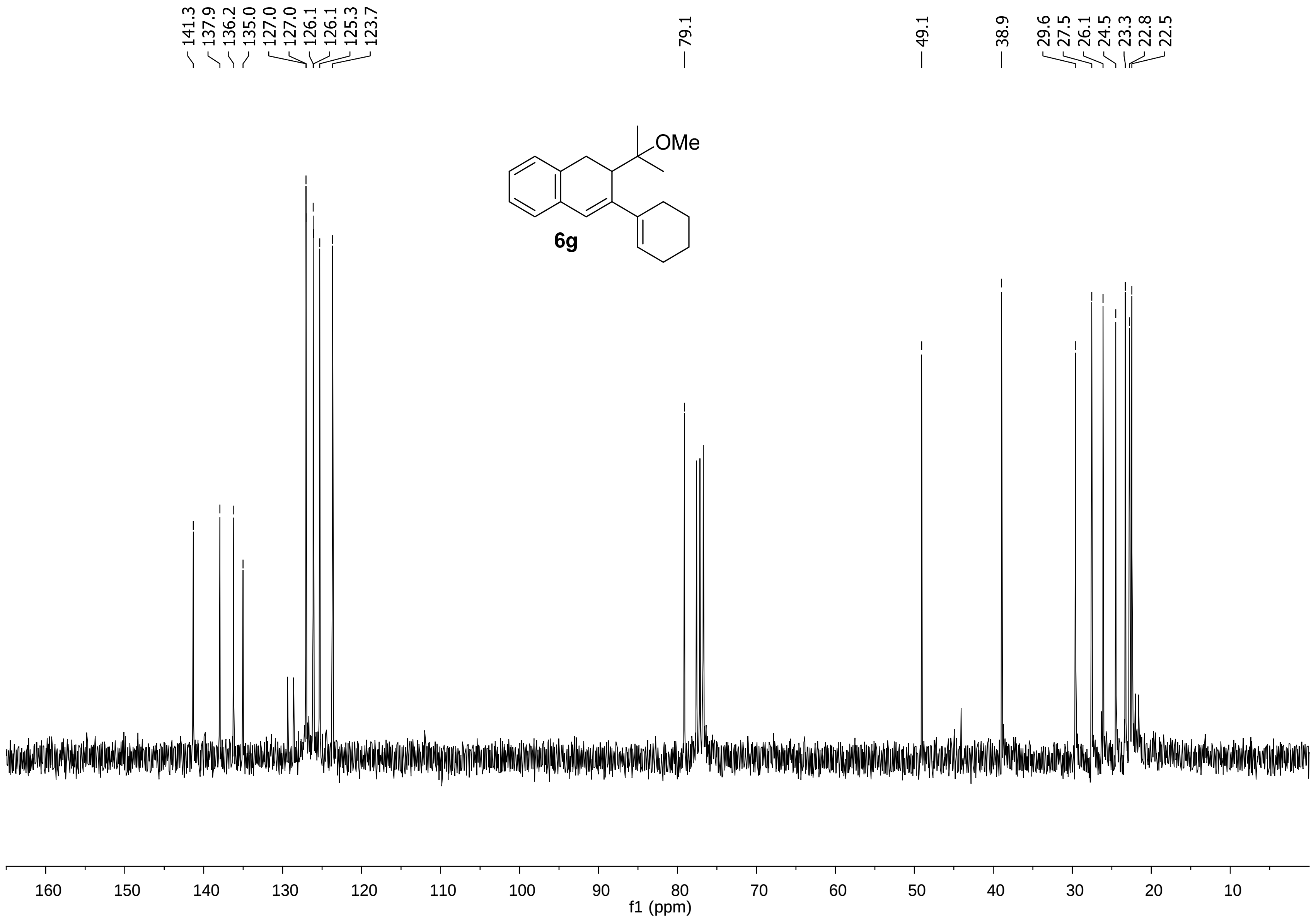
141.3
137.9
136.2
135.0
127.0
127.0
126.1
126.1
125.3
123.7
79.1
49.1
38.9
29.6
27.5
26.1
24.5
23.3
22.8
22.5
OMe
6g
160
150
140
130
120
110
100
90
80
70
60
50
40
30
20
10
f1 (ppm)

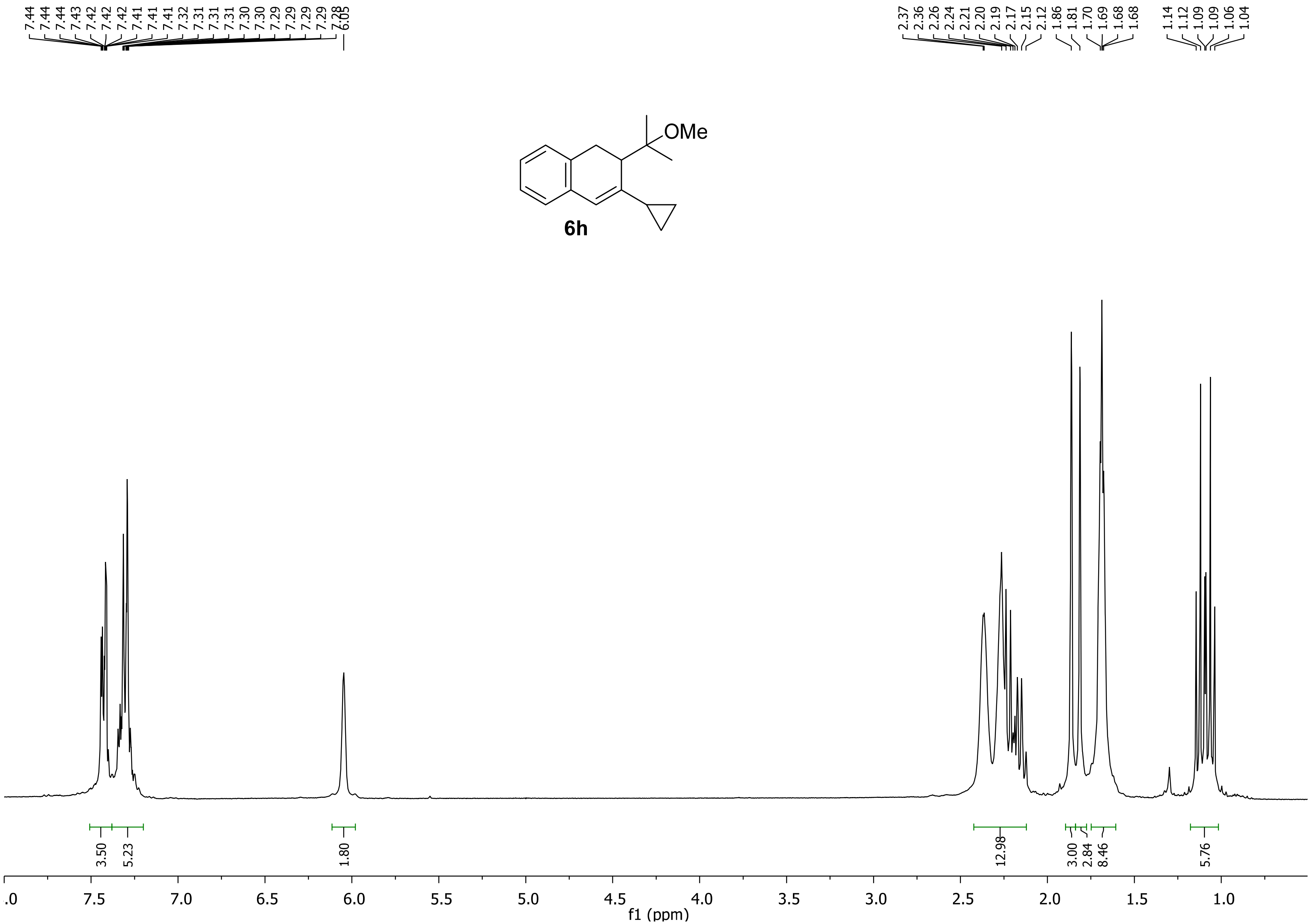
7.44 7.44 7.44 7.43 7.42 7.42 7.42 7.41 7.41 7.41 7.32 7.31 7.31 7.31 7.30 7.30 7.29 7.29 7.29 7.29 7.28 6.05
2.37 2.36 2.26 2.24 2.21 2.20 2.19 2.17 2.15 2.12 1.86 1.81 1.70 1.69 1.68 1.68
1.14 1.12 1.09 1.09 1.06 1.04
OMe
6h
3.50
5.23
1.80
12.98
3.00
2.84
8.46
5.76
.0 7.5 7.0 6.5 6.0 5.5 5.0 4.5 4.0 3.5 3.0 2.5 2.0 1.5 1.0
f1 (ppm)

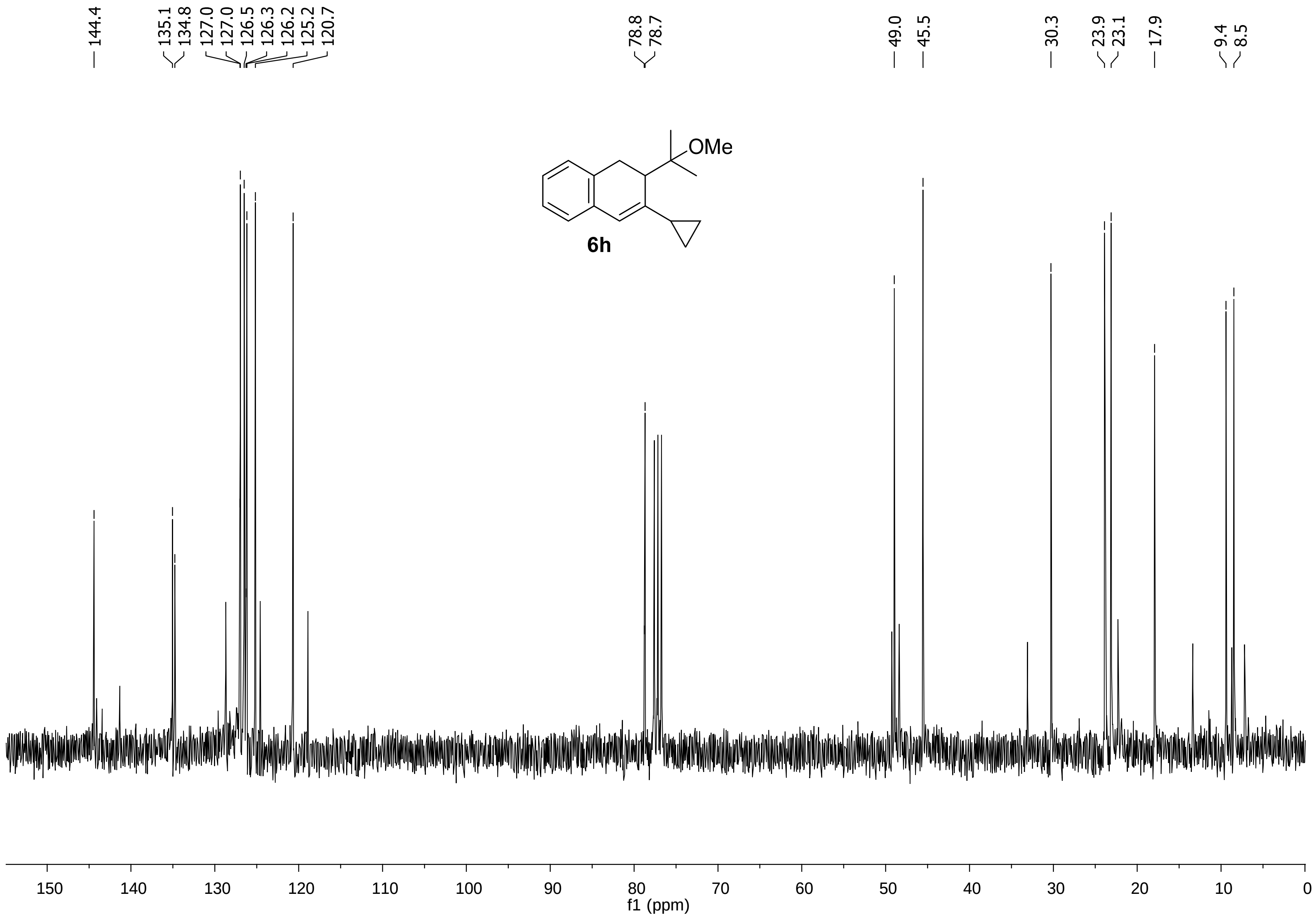
144.4
135.1
134.8
127.0
127.0
126.5
126.3
126.2
125.2
120.7
78.8
78.7
49.0
45.5
30.3
23.9
23.1
17.9
9.4
8.5
OMe
6h
150 140 130 120 110 100 90 80 70 60 50 40 30 20 10 0
f1 (ppm)

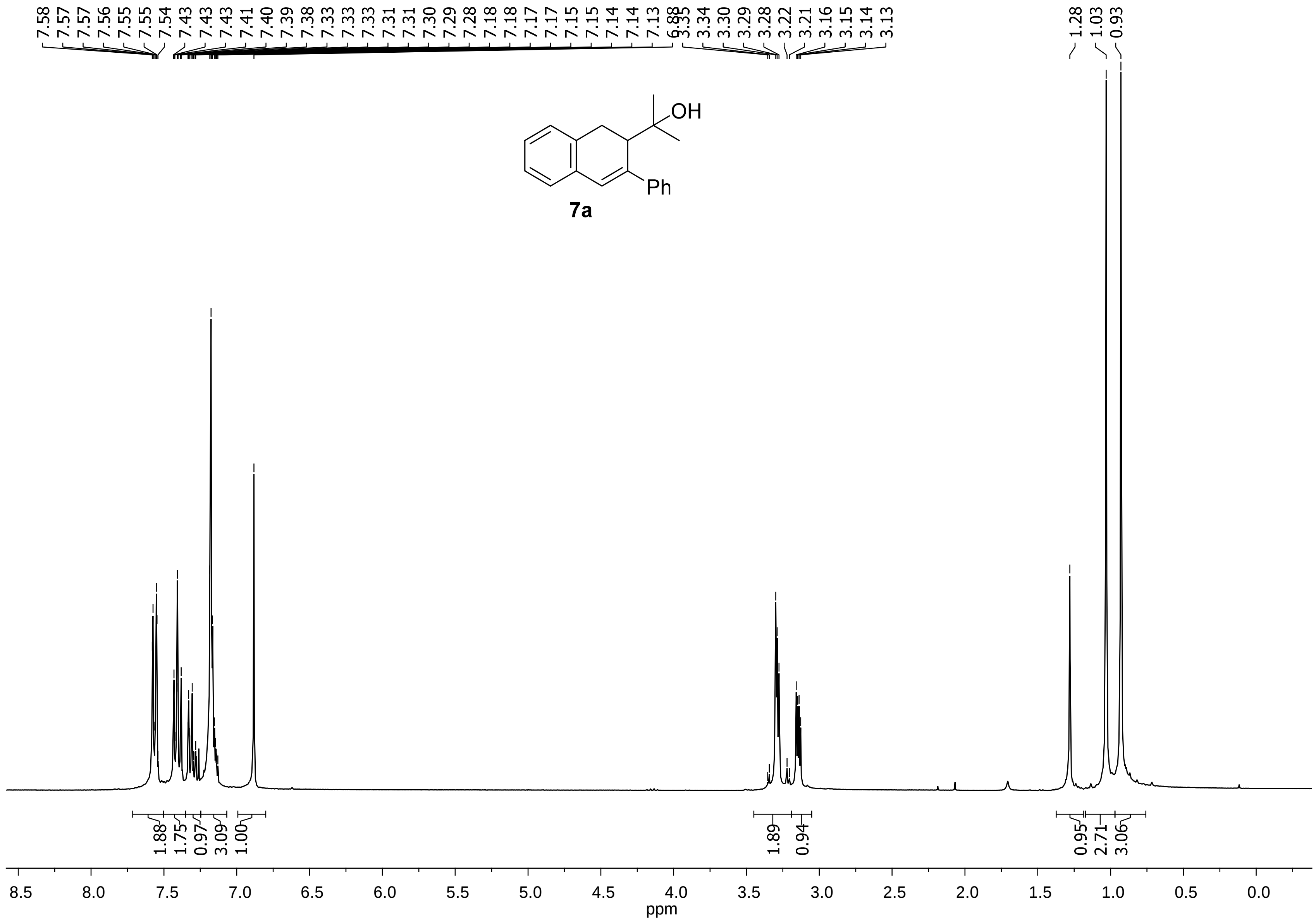
7.58 7.57 7.57 7.56 7.55 7.55 7.54 7.43 7.43 7.43 7.43 7.41 7.40 7.39 7.38 7.33 7.33 7.33 7.31 7.31 7.30 7.29 7.28 7.18 7.18 7.17 7.17 7.15 7.15 7.14 7.14 7.13 6.88 3.35 3.34 3.30 3.29 3.28 3.22 3.21 3.16 3.15 3.14 3.13
1.28 1.03 0.93
OH
Ph
7a
1.88 1.75 0.97 3.09 1.00
1.89 0.94
0.95 2.71 3.06
8.5 8.0 7.5 7.0 6.5 6.0 5.5 5.0 4.5 4.0 3.5 3.0 2.5 2.0 1.5 1.0 0.5 0.0
ppm

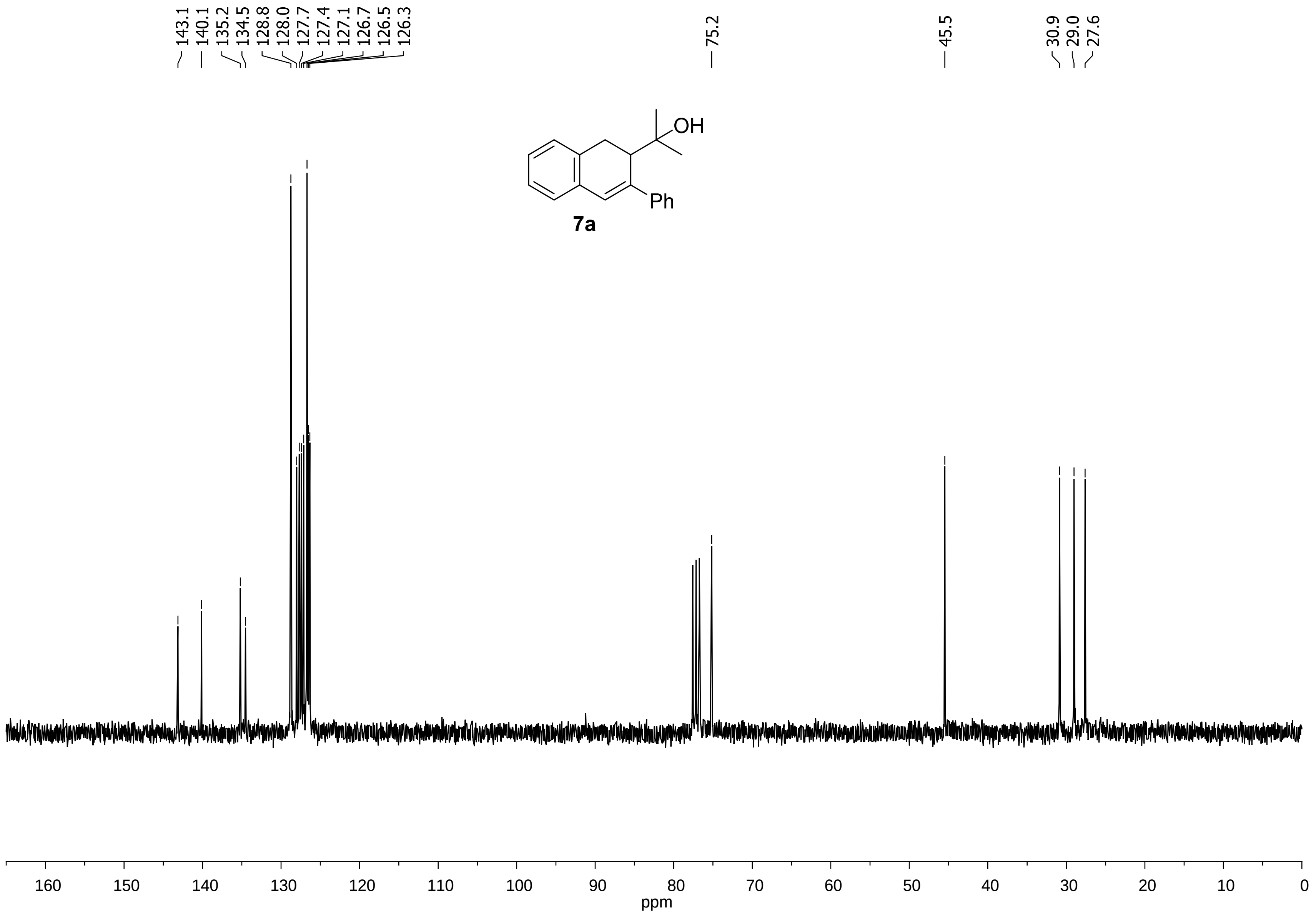
143.1
140.1
135.2
134.5
128.8
128.0
127.7
127.4
127.1
126.7
126.5
126.3
75.2
45.5
30.9
29.0
27.6
OH
Ph
7a
160 150 140 130 120 110 100 90 80 70 60 50 40 30 20 10 0
ppm

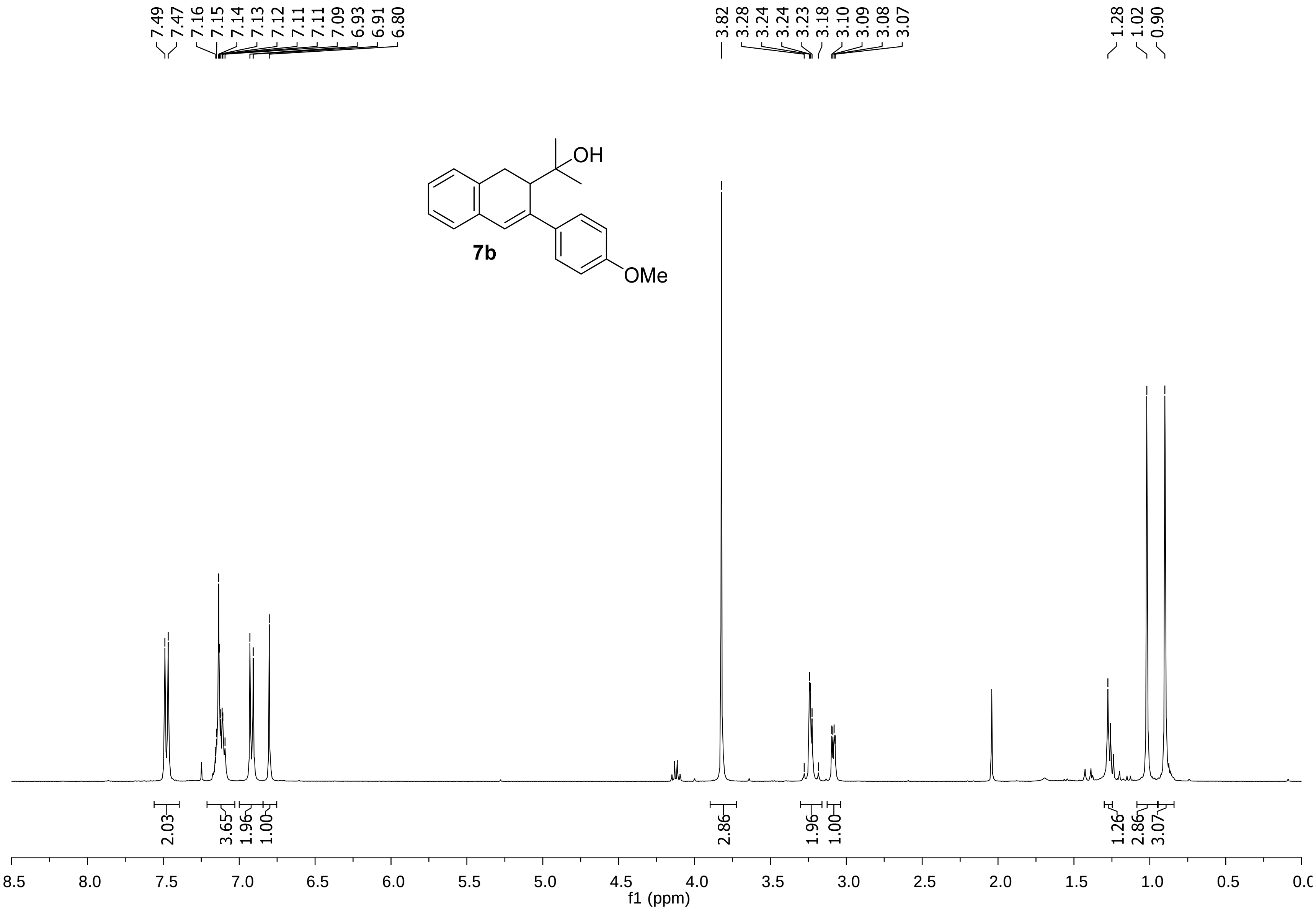
7.49
7.47
7.16
7.15
7.14
7.13
7.12
7.11
7.11
7.09
6.93
6.91
6.80
3.82
3.28
3.24
3.24
3.23
3.18
3.10
3.09
3.08
3.07
1.28
1.02
0.90
OH
OMe
7b
2.03
3.65
1.96
1.00
2.86
1.96
1.00
1.26
2.86
3.07
8.5
8.0
7.5
7.0
6.5
6.0
5.5
5.0
4.5
4.0
3.5
3.0
2.5
2.0
1.5
1.0
0.5
f1 (ppm)

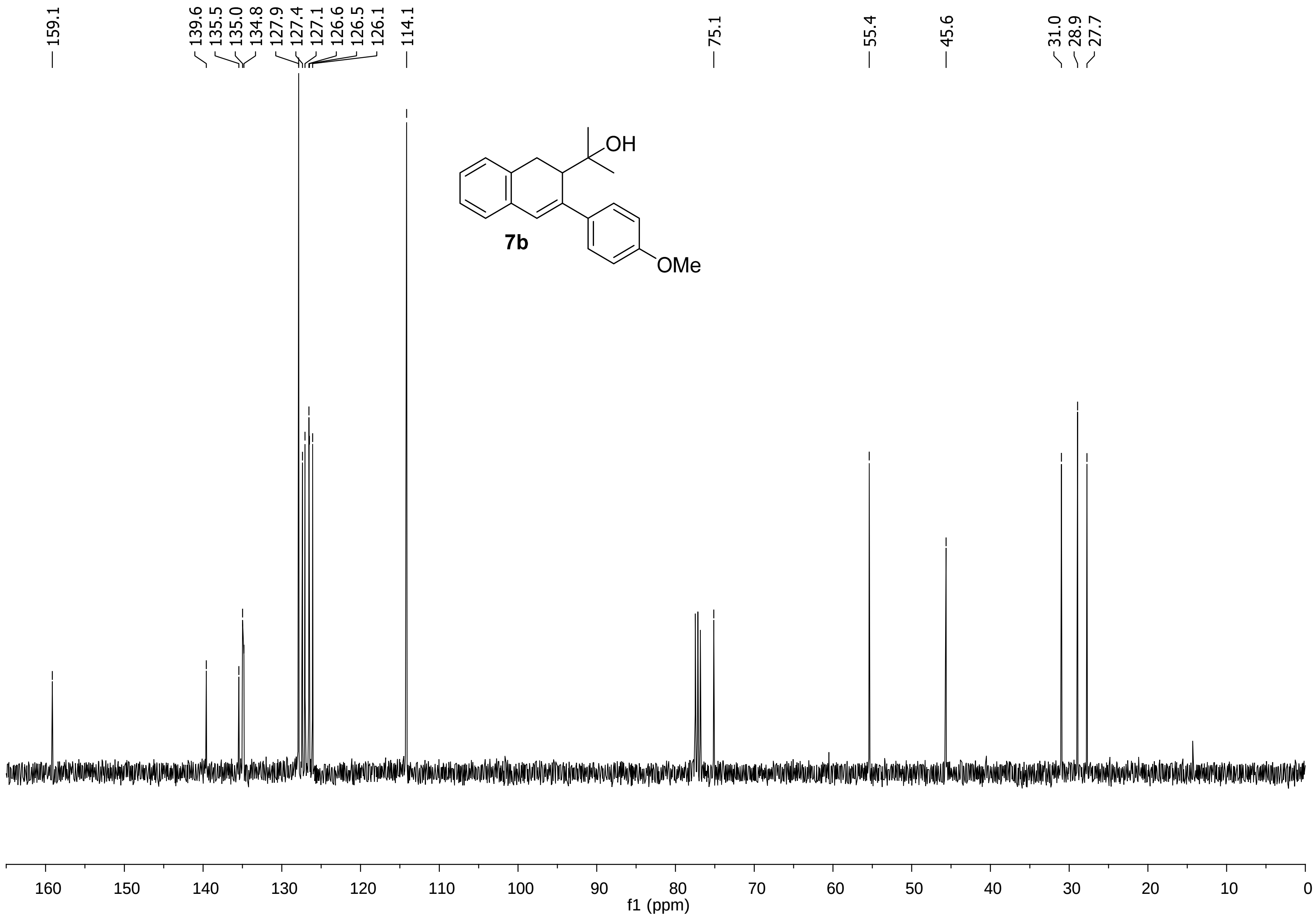

159.1
139.6
135.5
135.0
134.8
127.9
127.4
127.1
126.6
126.5
126.1
114.1
75.1
55.4
45.6
31.0
28.9
27.7
OH
7b
OMe
160
150
140
130
120
110
100
90
80
70
60
50
40
30
20
10
0
f1 (ppm)

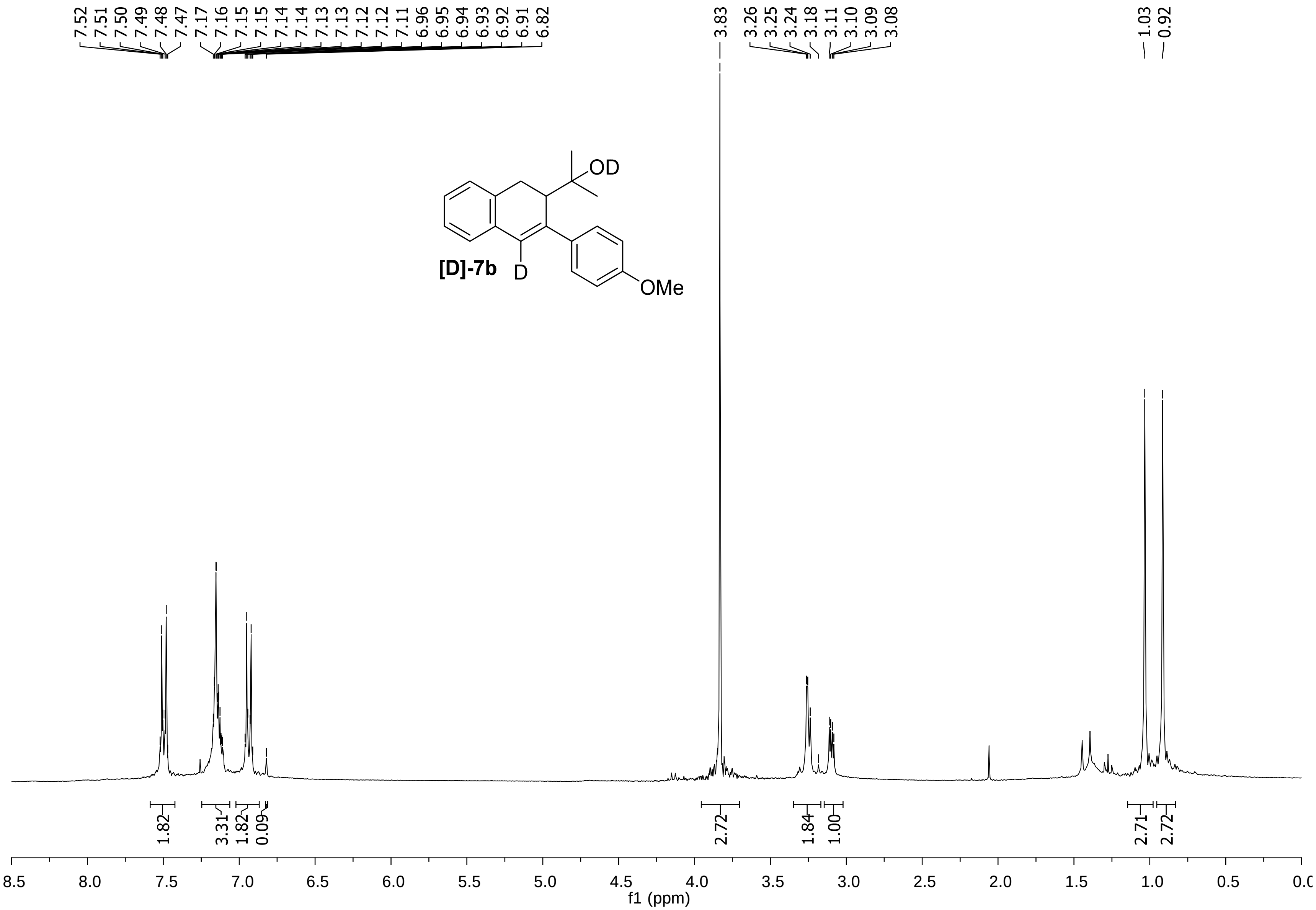

7.52 7.51 7.50 7.49 7.48 7.47 7.17 7.16 7.15 7.15 7.14 7.14 7.13 7.13 7.12 7.12 7.11 6.96 6.95 6.94 6.93 6.92 6.91 6.82
3.83
3.26 3.25 3.24 3.18 3.11 3.10 3.09 3.08
1.03
0.92
OD
D
OMe
[D]-7b
1.82 3.31 1.82 0.09
2.72
1.84 1.00
2.71 2.72
8.5 8.0 7.5 7.0 6.5 6.0 5.5 5.0 4.5 4.0 3.5 3.0 2.5 2.0 1.5 1.0 0.5 0.0
f1 (ppm)

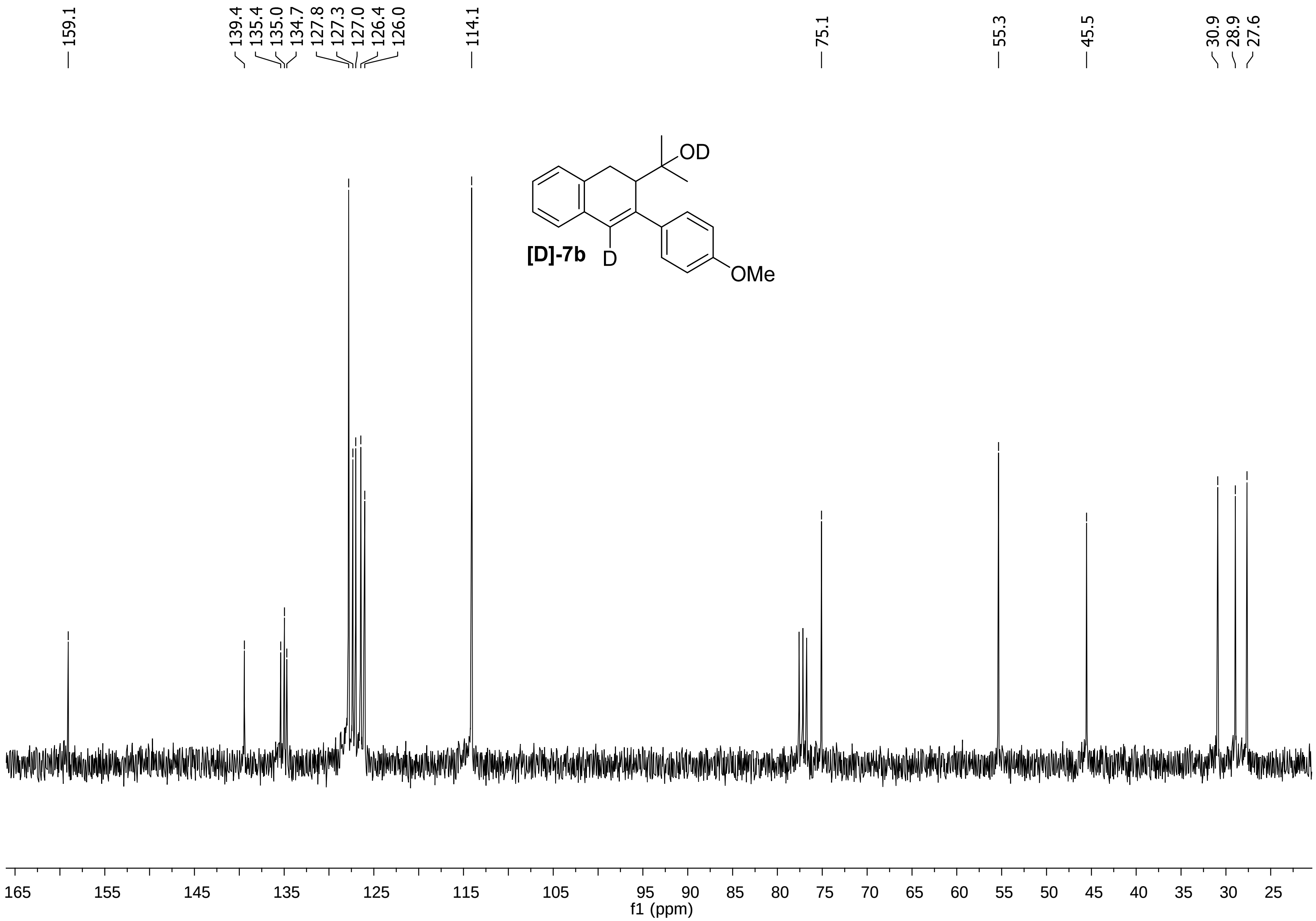
159.1
139.4
135.4
135.0
134.7
127.8
127.3
127.0
126.4
126.0
114.1
75.1
55.3
45.5
30.9
28.9
27.6
OD
[D]-7b
D
OMe
165
155
145
135
125
115
105
95
90
85
80
75
70
65
60
55
50
45
40
35
30
25
f1 (ppm)

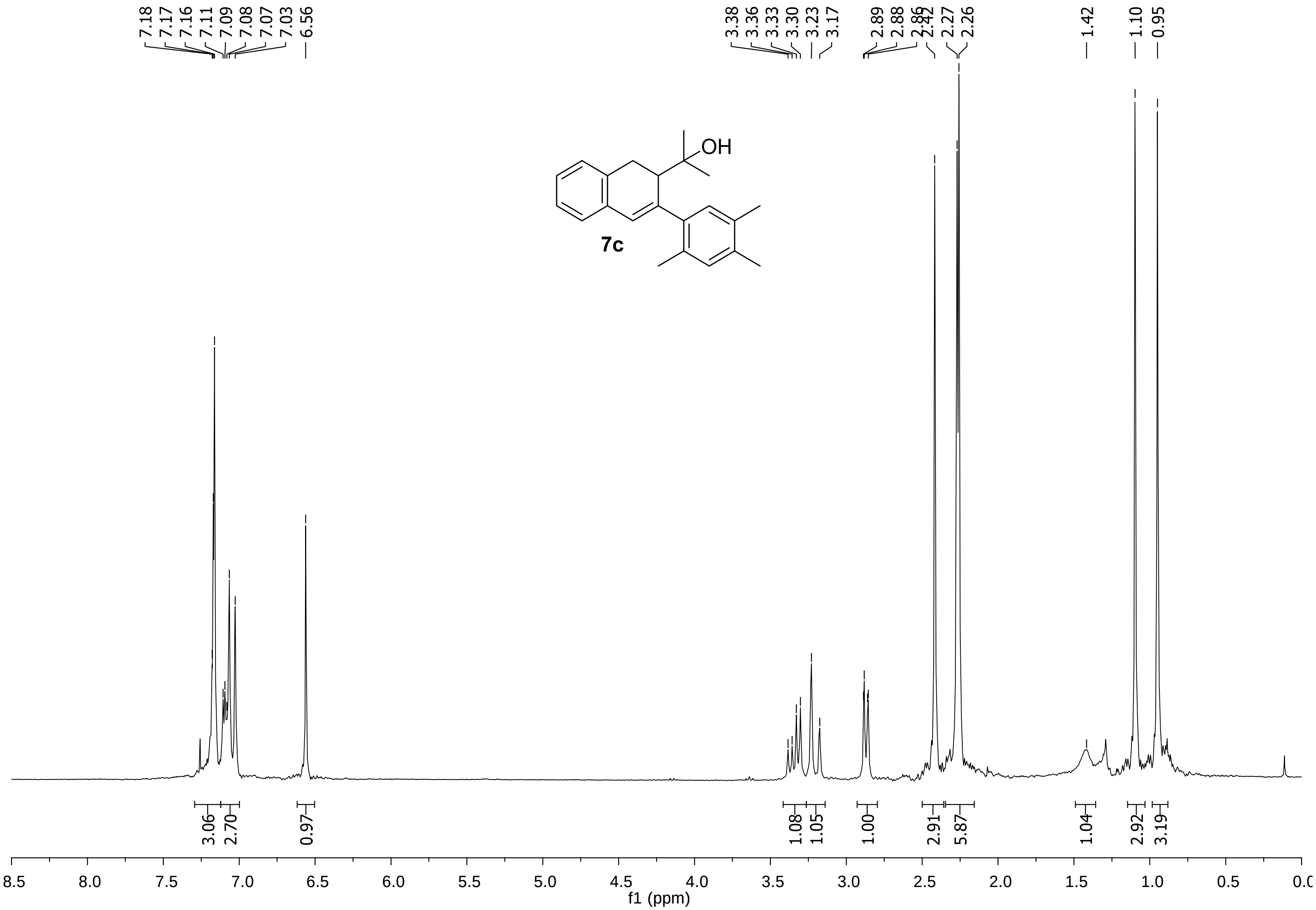
7.18
7.17
7.16
7.11
7.09
7.08
7.07
7.03
6.56
3.38
3.36
3.33
3.30
3.23
3.17
2.89
2.88
2.86
2.42
2.27
2.26
1.42
1.10
0.95
OH
7c
3.06
2.70
0.97
1.08
1.05
1.00
2.91
5.87
1.04
2.92
3.19
8.5
8.0
7.5
7.0
6.5
6.0
5.5
5.0
4.5
4.0
3.5
3.0
2.5
2.0
1.5
1.0
0.5
f1 (ppm)

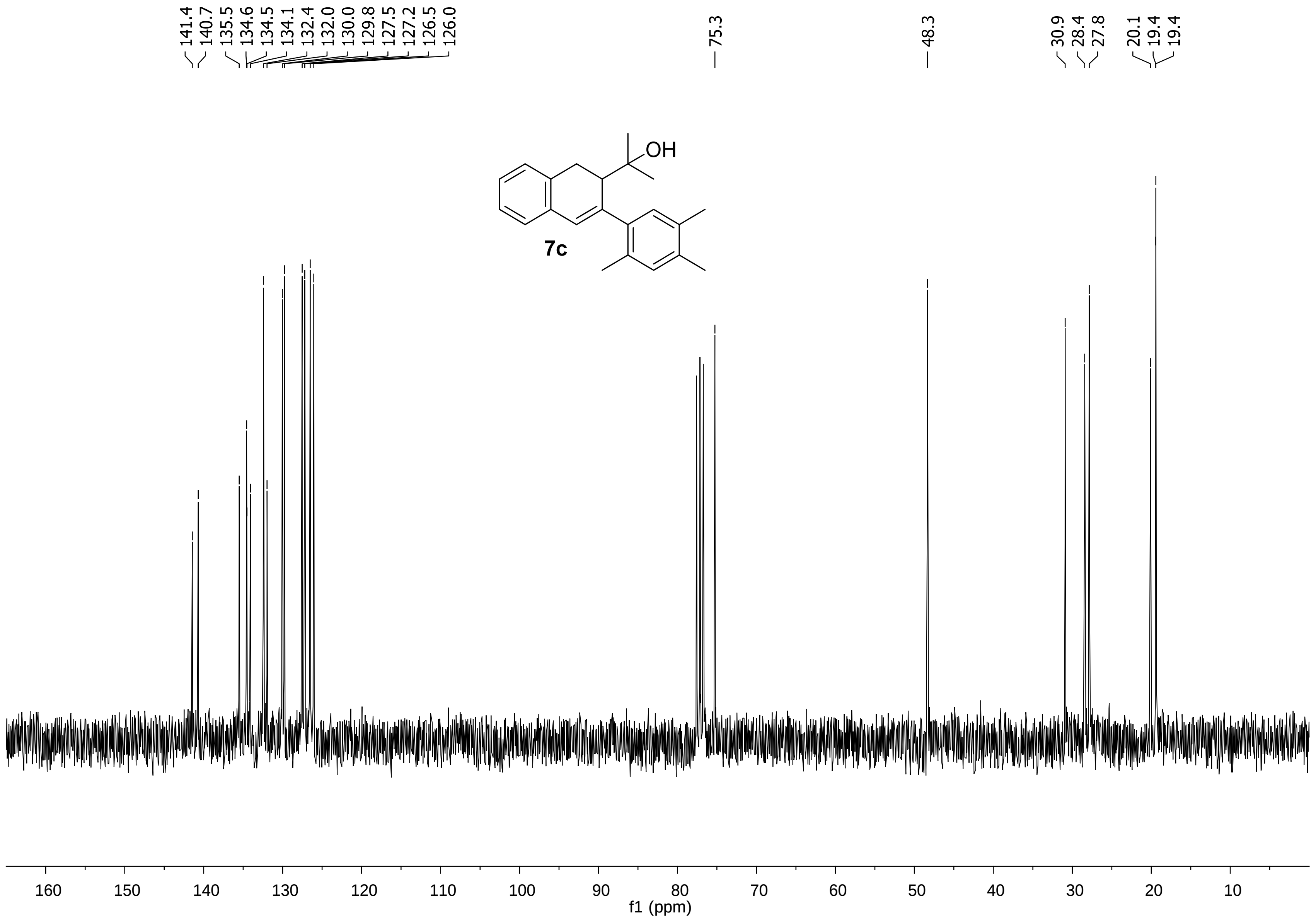
141.4
140.7
135.5
134.6
134.5
134.1
132.4
132.0
130.0
129.8
127.5
127.2
126.5
126.0
75.3
48.3
30.9
28.4
27.8
20.1
19.4
19.4
OH
7c
160 150 140 130 120 110 100 90 80 70 60 50 40 30 20 10
f1 (ppm)

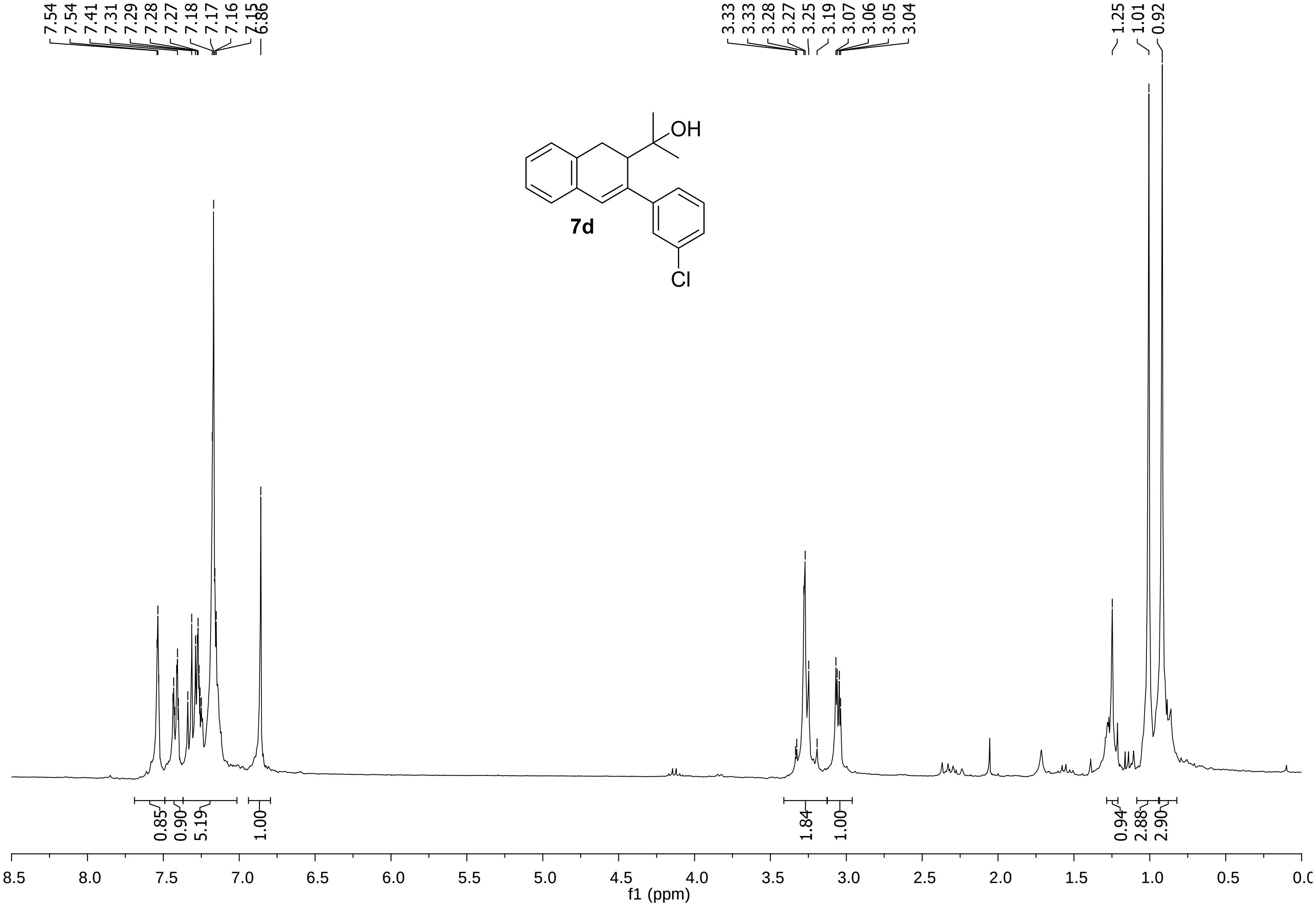
7.54
7.54
7.41
7.31
7.29
7.28
7.27
7.18
7.17
7.16
7.15
6.86
3.33
3.33
3.28
3.27
3.25
3.19
3.07
3.06
3.05
3.04
1.25
1.01
0.92
OH
Cl
7d
0.85
0.90
5.19
1.00
1.84
1.00
0.94
2.88
2.90
8.5
8.0
7.5
7.0
6.5
6.0
5.5
5.0
4.5
4.0
3.5
3.0
2.5
2.0
1.5
1.0
0.5
f1 (ppm)

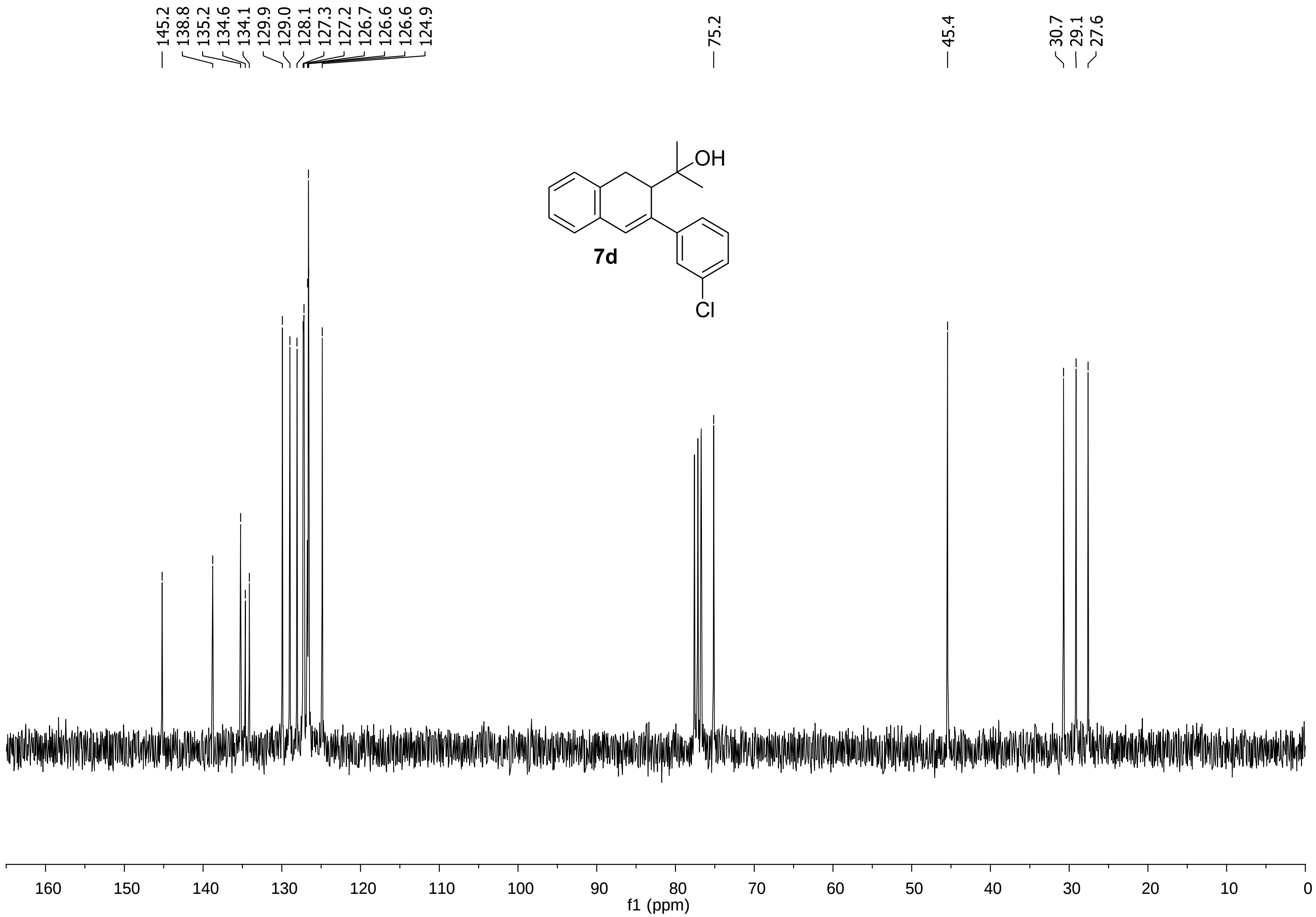
145.2
138.8
135.2
134.6
134.1
129.9
129.0
128.1
127.3
127.2
126.7
126.6
126.6
124.9
75.2
45.4
30.7
29.1
27.6
OH
Cl
7d
160 150 140 130 120 110 100 90 80 70 60 50 40 30 20 10 0
f1 (ppm)

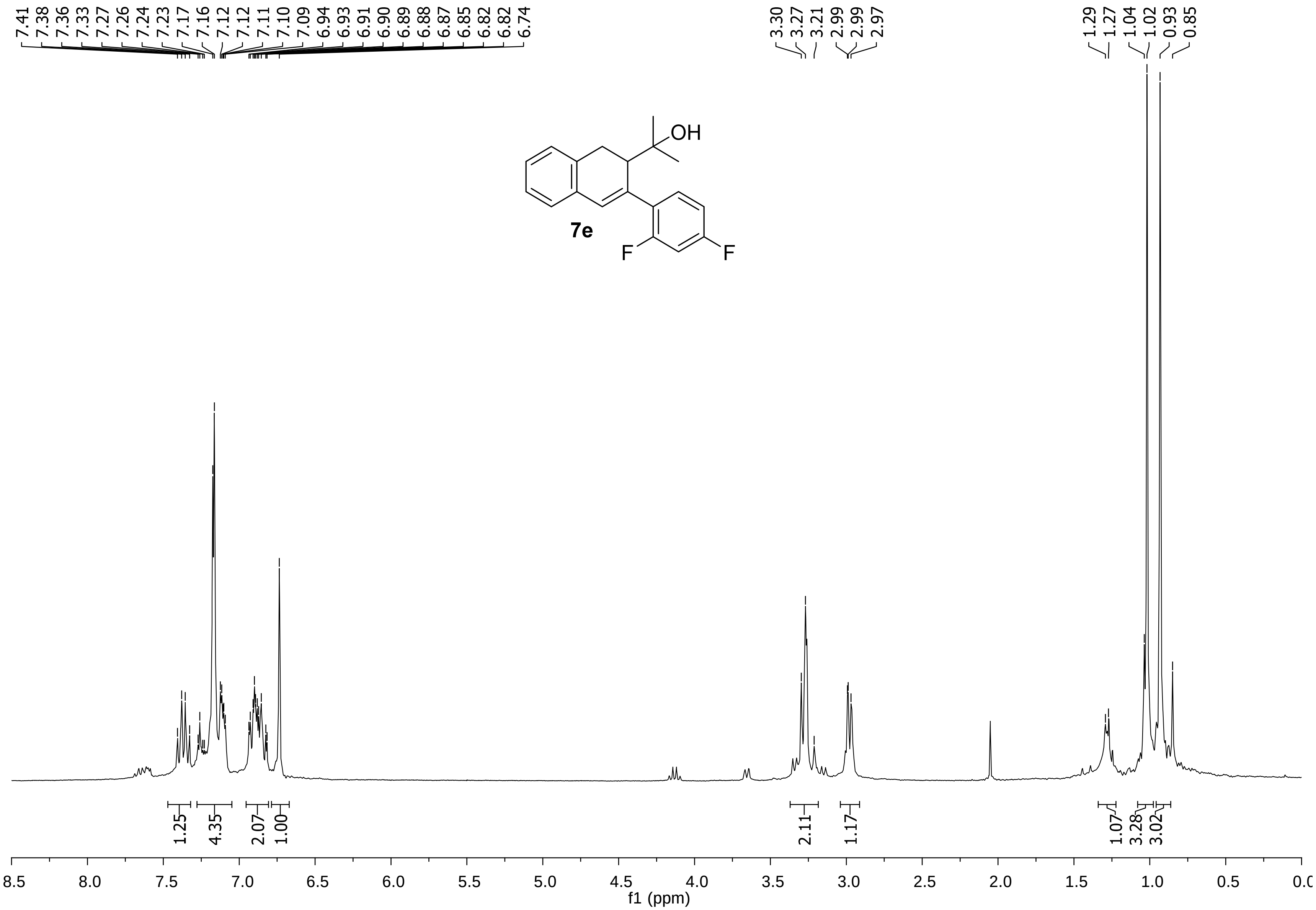
7.41 7.38 7.36 7.33 7.27 7.26 7.24 7.23 7.17 7.16 7.12 7.12 7.11 7.10 7.09 6.94 6.93 6.91 6.90 6.89 6.88 6.87 6.85 6.82 6.82 6.74
3.30 3.27 3.21 2.99 2.99 2.97
1.29 1.27 1.04 1.02 0.93 0.85
OH
F
F
7e
1.25 4.35 2.07 1.00 2.11 1.17 1.07 3.28 3.02
8.5 8.0 7.5 7.0 6.5 6.0 5.5 5.0 4.5 4.0 3.5 3.0 2.5 2.0 1.5 1.0 0.5 0.0
f1 (ppm)

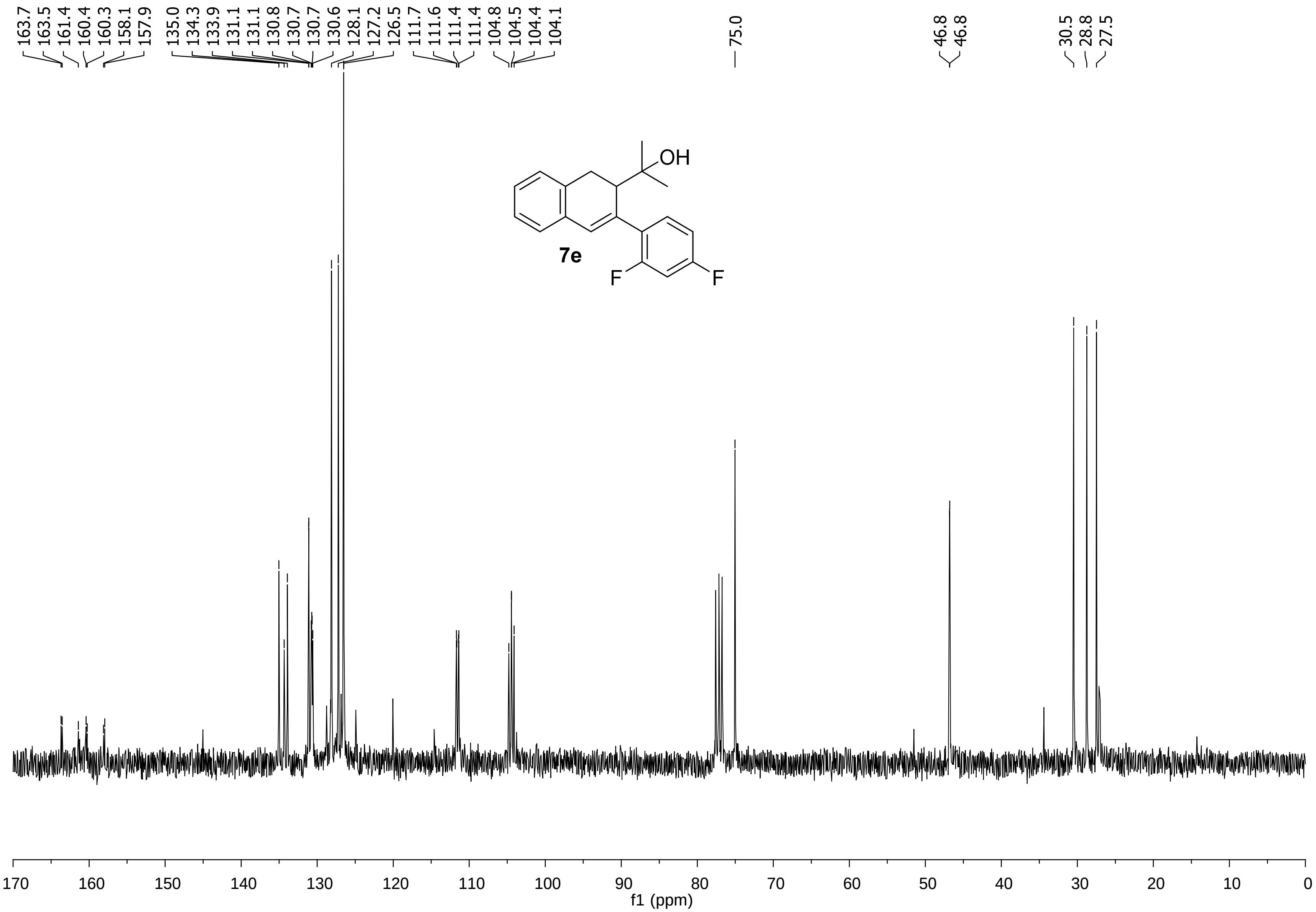
163.7
163.5
161.4
160.4
160.3
158.1
157.9
135.0
134.3
133.9
131.1
131.1
130.8
130.7
130.7
130.6
128.1
127.2
126.5
111.7
111.6
111.4
111.4
104.8
104.5
104.4
104.1
75.0
46.8
46.8
30.5
28.8
27.5
OH
7e
F
F
170 160 150 140 130 120 110 100 90 80 70 60 50 40 30 20 10 0
f1 (ppm)

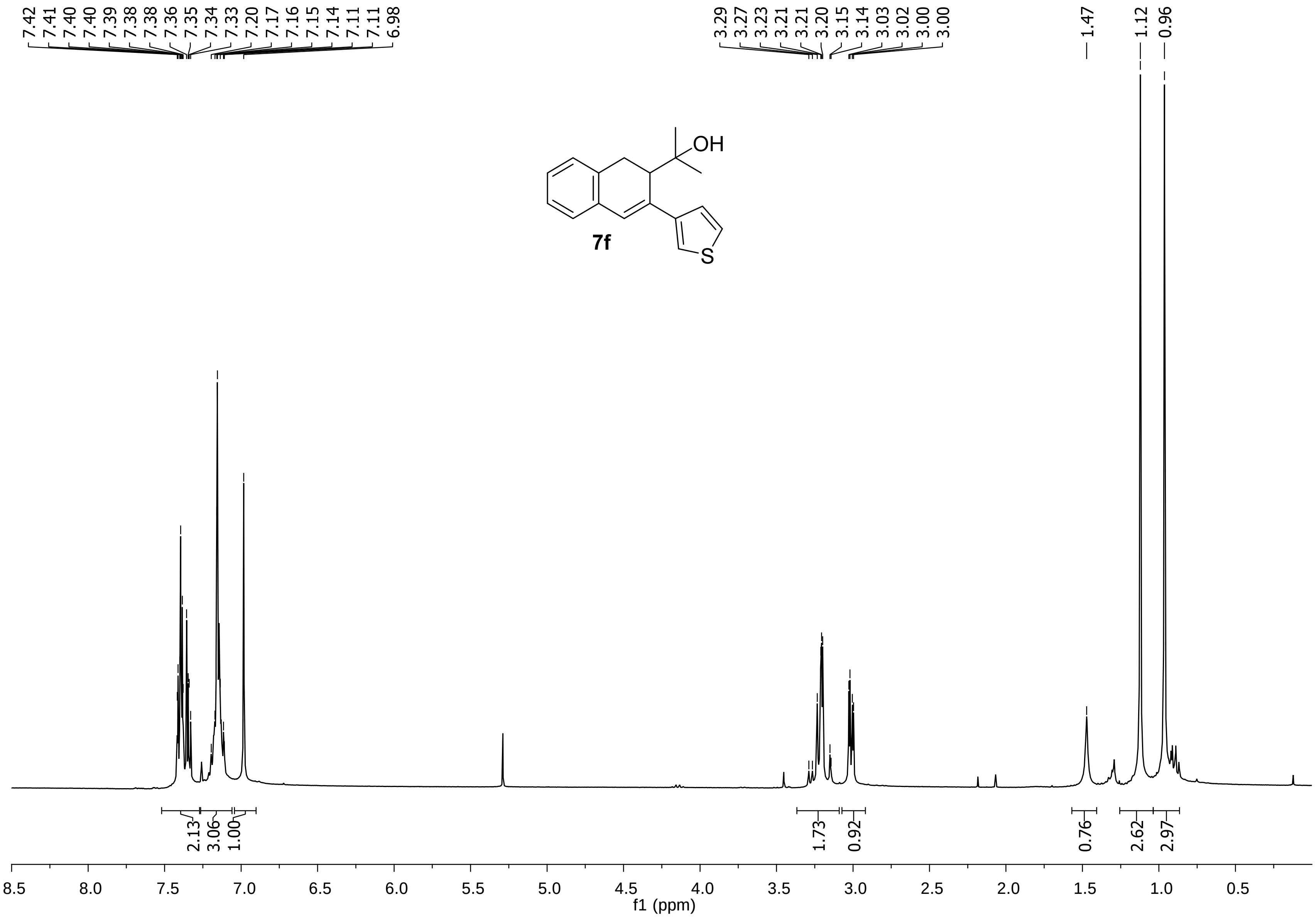

7.42
7.41
7.40
7.40
7.39
7.38
7.38
7.36
7.35
7.34
7.33
7.20
7.17
7.16
7.15
7.14
7.11
7.11
6.98
3.29
3.27
3.23
3.21
3.21
3.20
3.15
3.14
3.03
3.02
3.00
3.00
1.47
1.12
0.96
OH
S
7f
2.13
3.06
1.00
1.73
0.92
0.76
2.62
2.97
8.5
8.0
7.5
7.0
6.5
6.0
5.5
5.0
4.5
4.0
3.5
3.0
2.5
2.0
1.5
1.0
0.5
f1 (ppm)

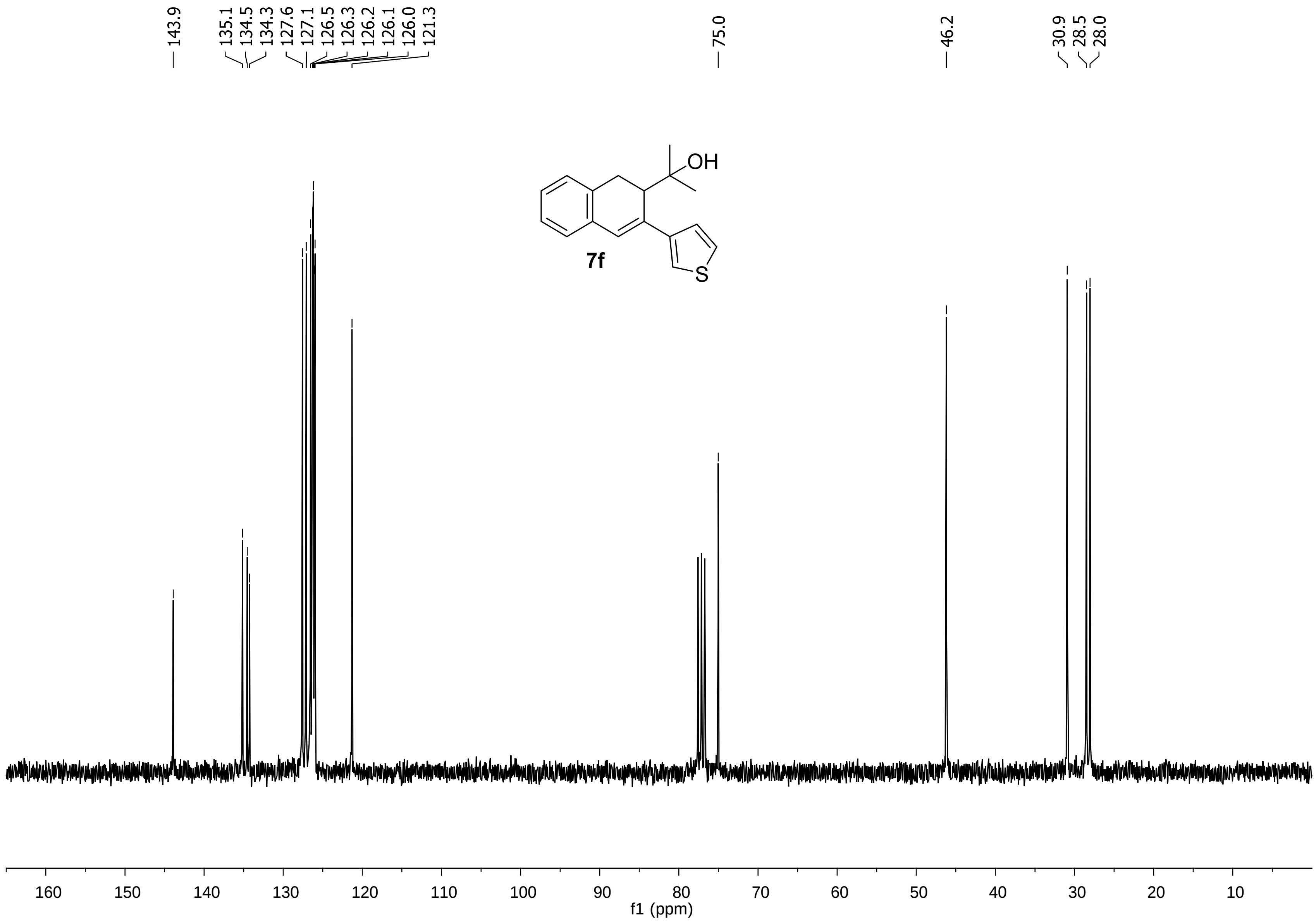
143.9
135.1
134.5
134.3
127.6
127.1
126.5
126.3
126.2
126.1
126.0
121.3
75.0
46.2
30.9
28.5
28.0
OH
S
7f
160
150
140
130
120
110
100
90
80
70
60
50
40
30
20
10
f1 (ppm)

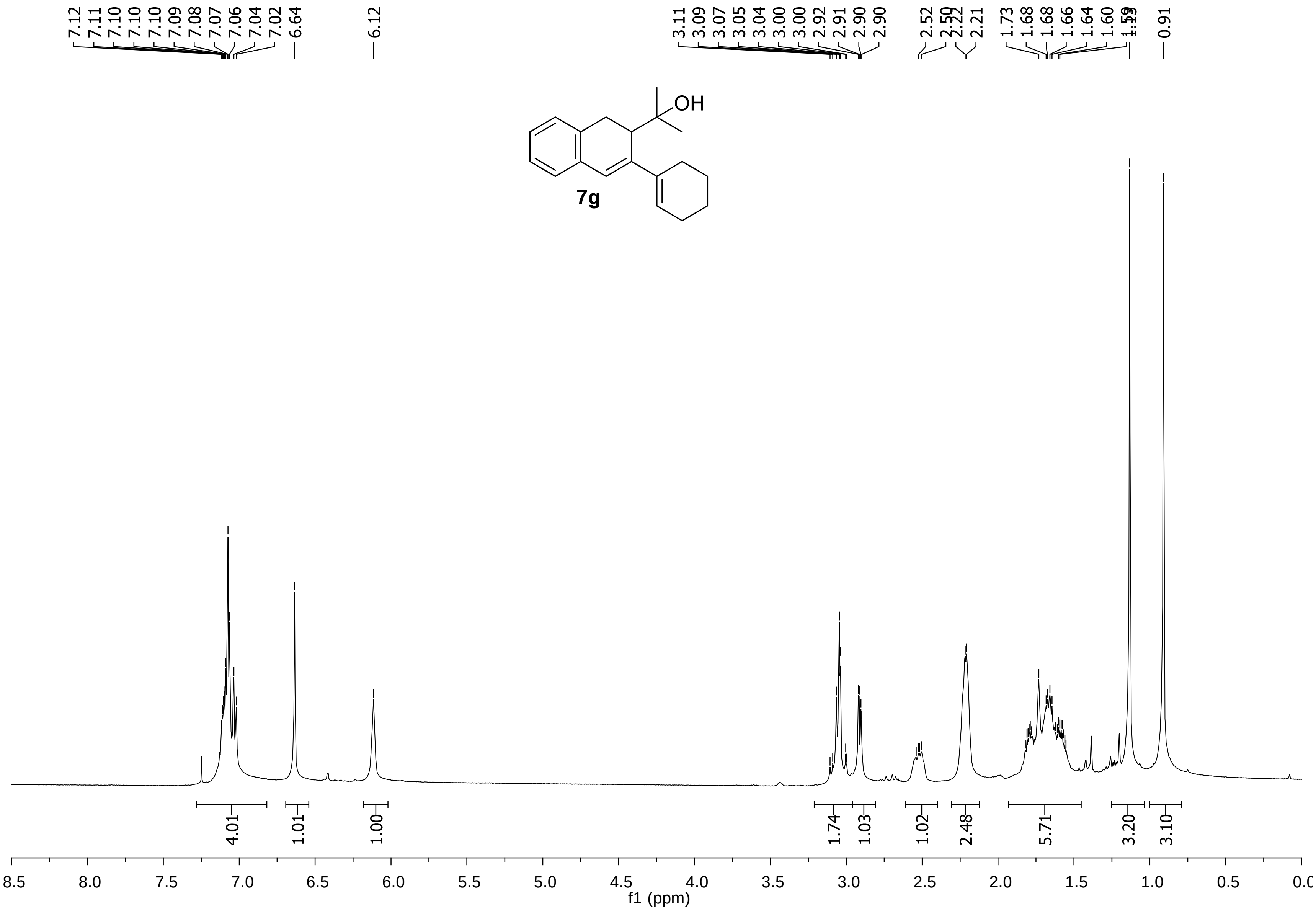
7.12
7.11
7.10
7.10
7.10
7.09
7.08
7.07
7.06
7.04
7.02
6.64
6.12
3.11
3.09
3.07
3.05
3.04
3.00
3.00
2.92
2.91
2.90
2.90
2.52
2.50
2.22
2.21
1.73
1.68
1.68
1.66
1.64
1.60
1.59
1.13
0.91
OH
7g
4.01
1.01
1.00
1.74
1.03
1.02
2.48
5.71
3.20
3.10
8.5
8.0
7.5
7.0
6.5
6.0
5.5
5.0
4.5
4.0
3.5
3.0
2.5
2.0
1.5
1.0
0.5
f1 (ppm)

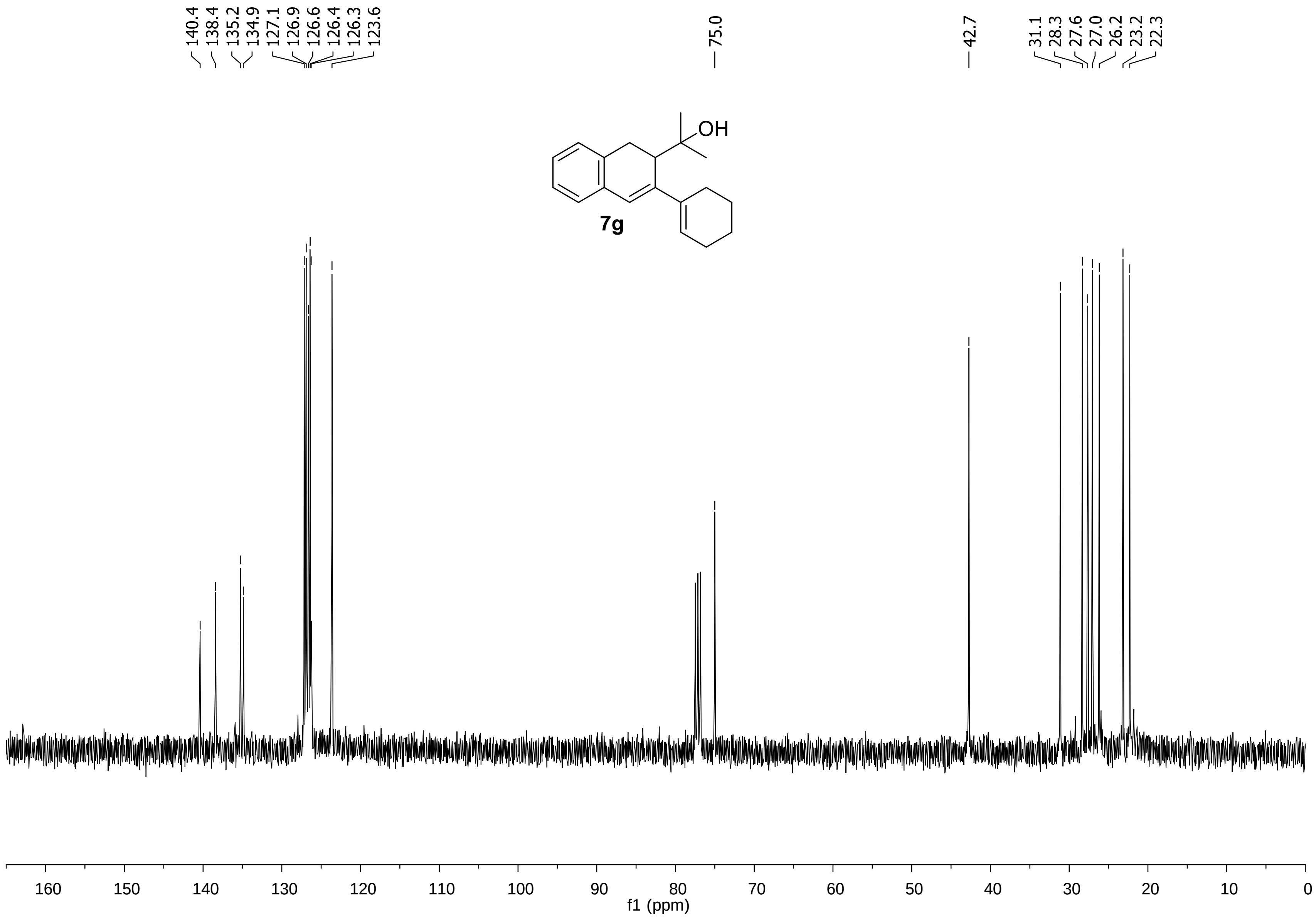

140.4
138.4
135.2
134.9
127.1
126.9
126.6
126.4
126.3
123.6
75.0
42.7
31.1
28.3
27.6
27.0
26.2
23.2
22.3
OH
7g
160
150
140
130
120
110
100
90
80
70
60
50
40
30
20
10
0
f1 (ppm)

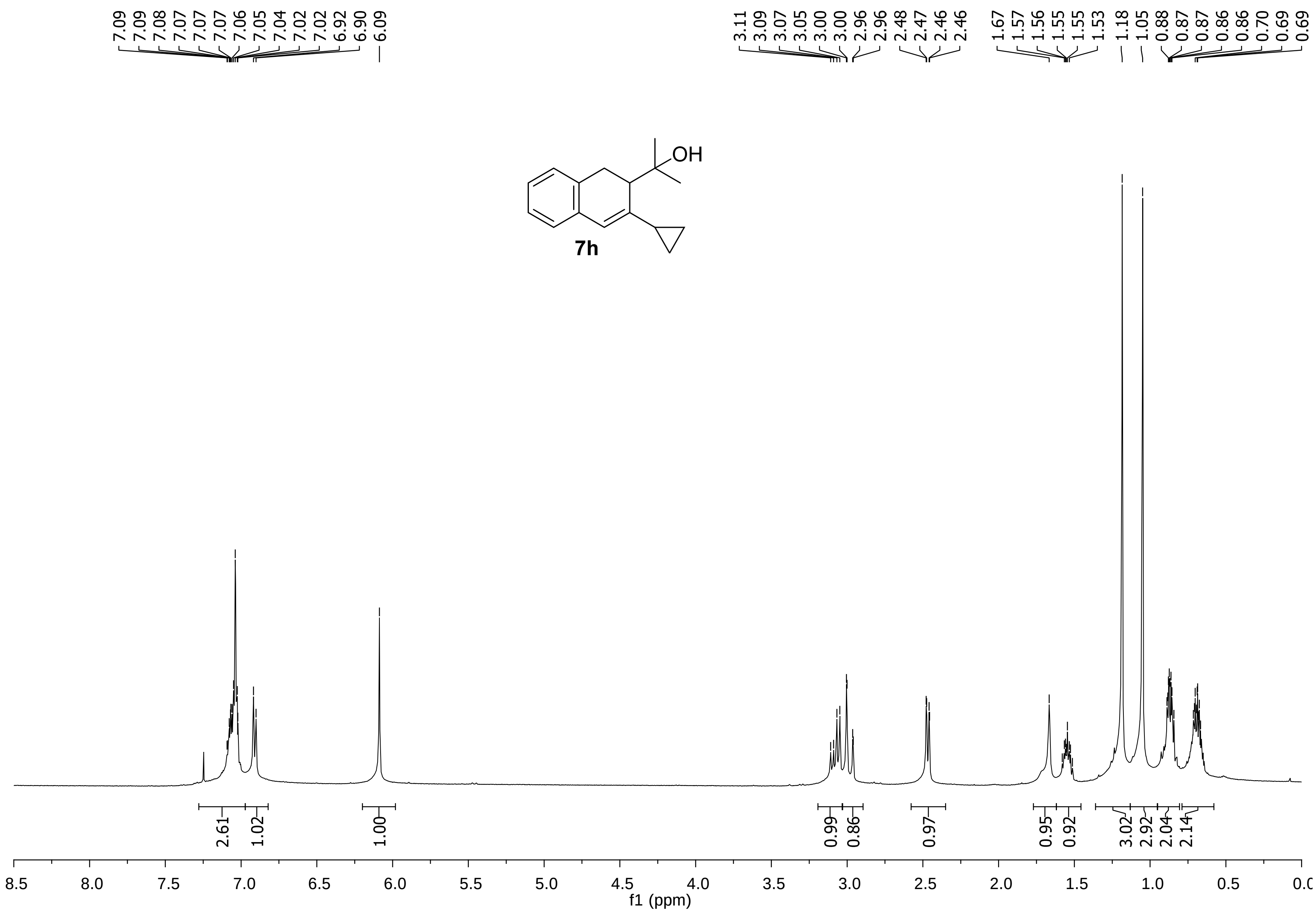
7.09 7.09 7.08 7.07 7.07 7.07 7.06 7.05 7.04 7.02 7.02 6.92 6.90 6.09
3.11 3.09 3.07 3.05 3.00 3.00 2.96 2.96 2.48 2.47 2.46 2.46
1.67 1.57 1.56 1.55 1.55 1.53 1.18 1.05 0.88 0.87 0.87 0.86 0.86 0.70 0.69 0.69
OH
7h
2.61 1.02 1.00 0.99 0.86 0.97 0.95 0.92 3.02 2.92 2.04 2.14
8.5 8.0 7.5 7.0 6.5 6.0 5.5 5.0 4.5 4.0 3.5 3.0 2.5 2.0 1.5 1.0 0.5 0.0
f1 (ppm)

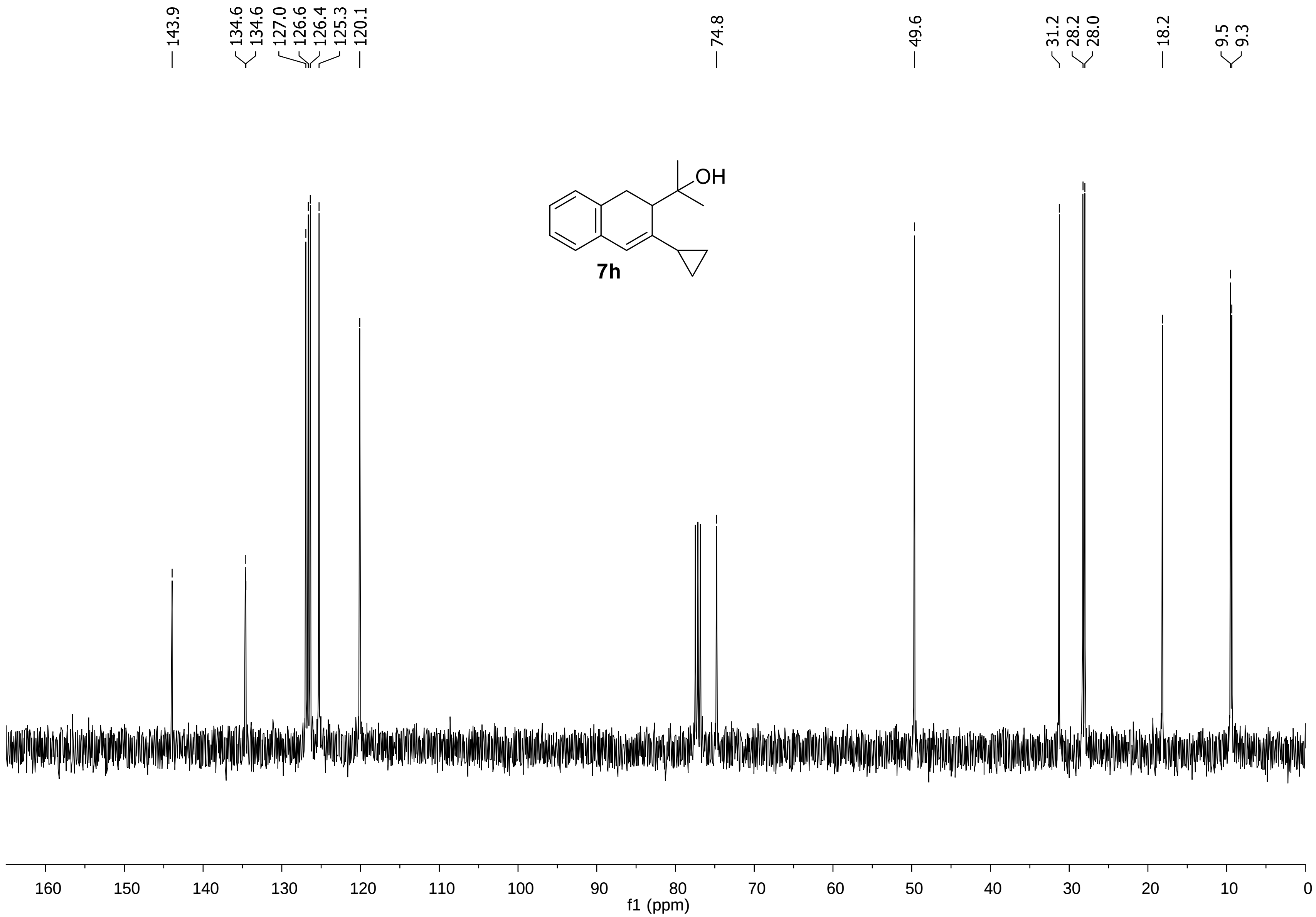
143.9
134.6
134.6
127.0
126.6
126.4
125.3
120.1
74.8
49.6
31.2
28.2
28.0
18.2
9.5
9.3
OH
7h
160 150 140 130 120 110 100 90 80 70 60 50 40 30 20 10 0
f1 (ppm)

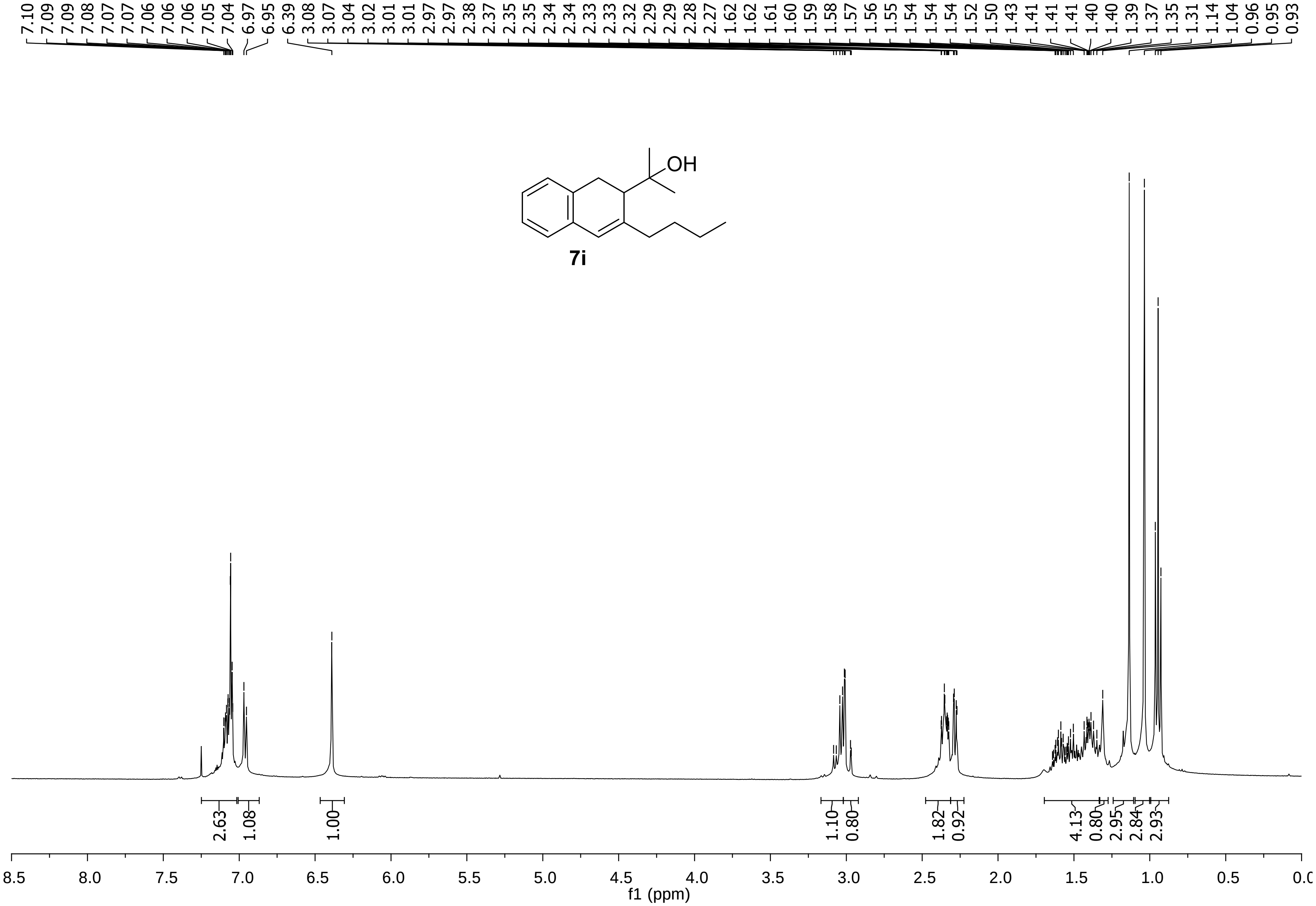

7.10
7.09
7.09
7.08
7.07
7.07
7.06
7.06
7.06
7.05
7.04
6.97
6.95
6.39
3.08
3.07
3.04
3.02
3.01
3.01
2.97
2.97
2.38
2.37
2.35
2.35
2.34
2.34
2.33
2.33
2.32
2.29
2.29
2.28
2.27
1.62
1.62
1.61
1.60
1.59
1.58
1.57
1.56
1.55
1.54
1.54
1.54
1.52
1.50
1.43
1.41
1.41
1.41
1.40
1.40
1.39
1.37
1.35
1.31
1.14
1.04
0.96
0.95
0.93
OH
7i
2.63
1.08
1.00
1.10
0.80
1.82
0.92
4.13
0.80
2.95
2.84
2.93
8.5
8.0
7.5
7.0
6.5
6.0
5.5
5.0
4.5
4.0
3.5
3.0
2.5
2.0
1.5
1.0
0.5
f1 (ppm)

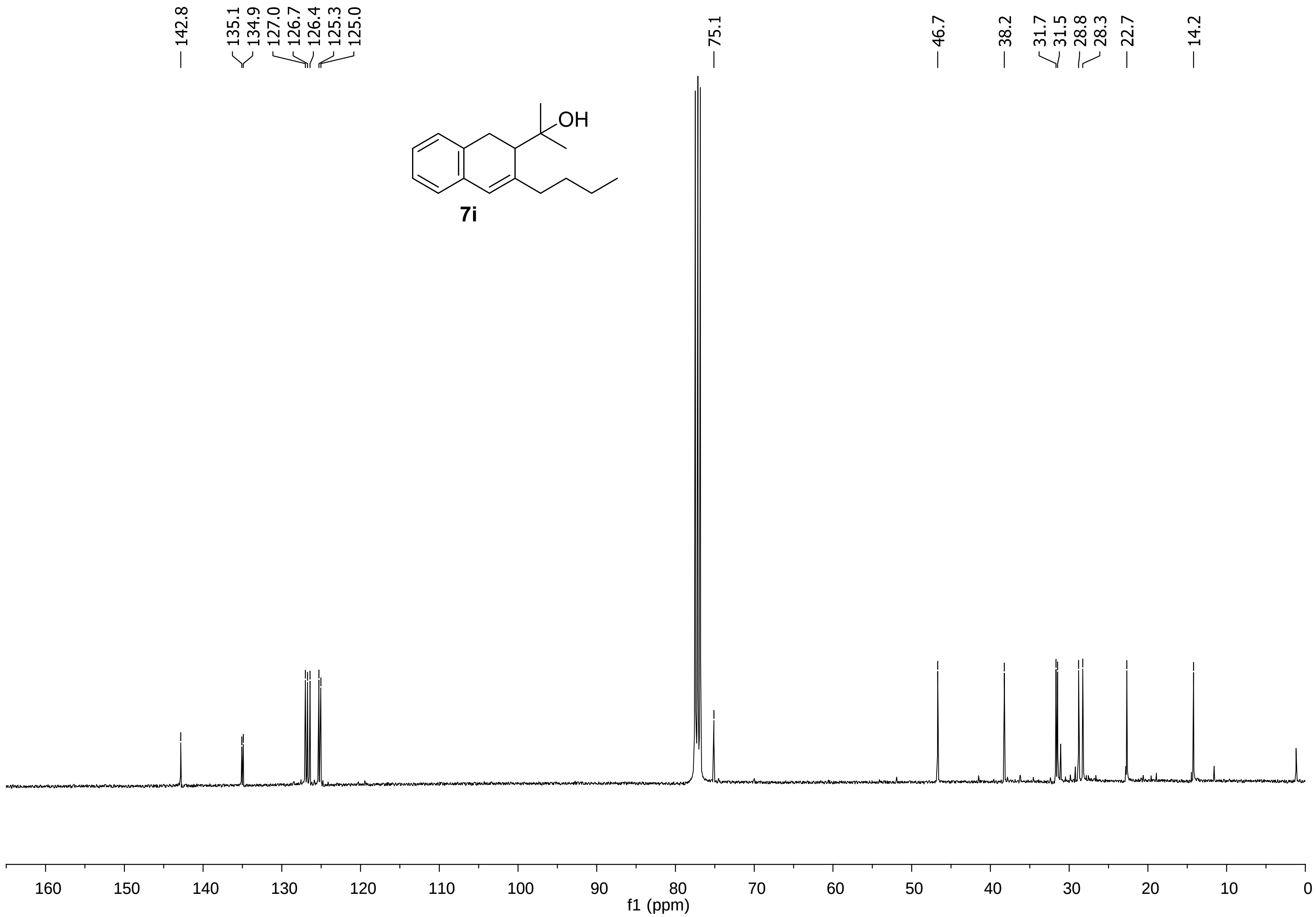
142.8
135.1
134.9
127.0
126.7
126.4
125.3
125.0
75.1
46.7
38.2
31.7
31.5
28.8
28.3
22.7
14.2
OH
7i
160 150 140 130 120 110 100 90 80 70 60 50 40 30 20 10 0
f1 (ppm)

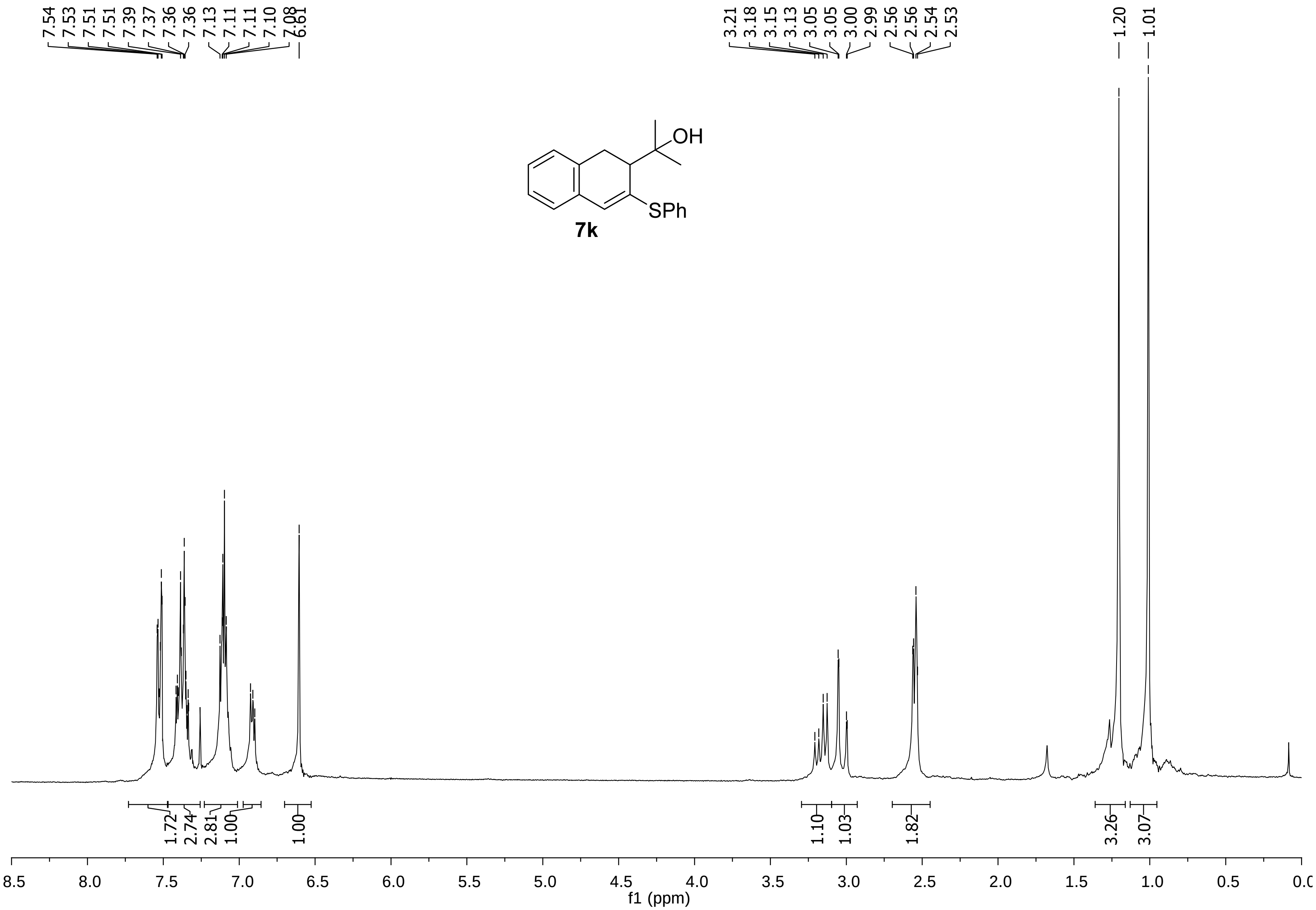
7.54
7.53
7.51
7.51
7.39
7.37
7.36
7.36
7.13
7.11
7.11
7.10
7.08
6.61
3.21
3.18
3.15
3.13
3.05
3.05
3.00
2.99
2.56
2.56
2.54
2.53
1.20
1.01
OH
SPh
7k
1.72
2.74
2.81
1.00
1.00
1.10
1.03
1.82
3.26
3.07
8.5
8.0
7.5
7.0
6.5
6.0
5.5
5.0
4.5
4.0
3.5
3.0
2.5
2.0
1.5
1.0
0.5
f1 (ppm)

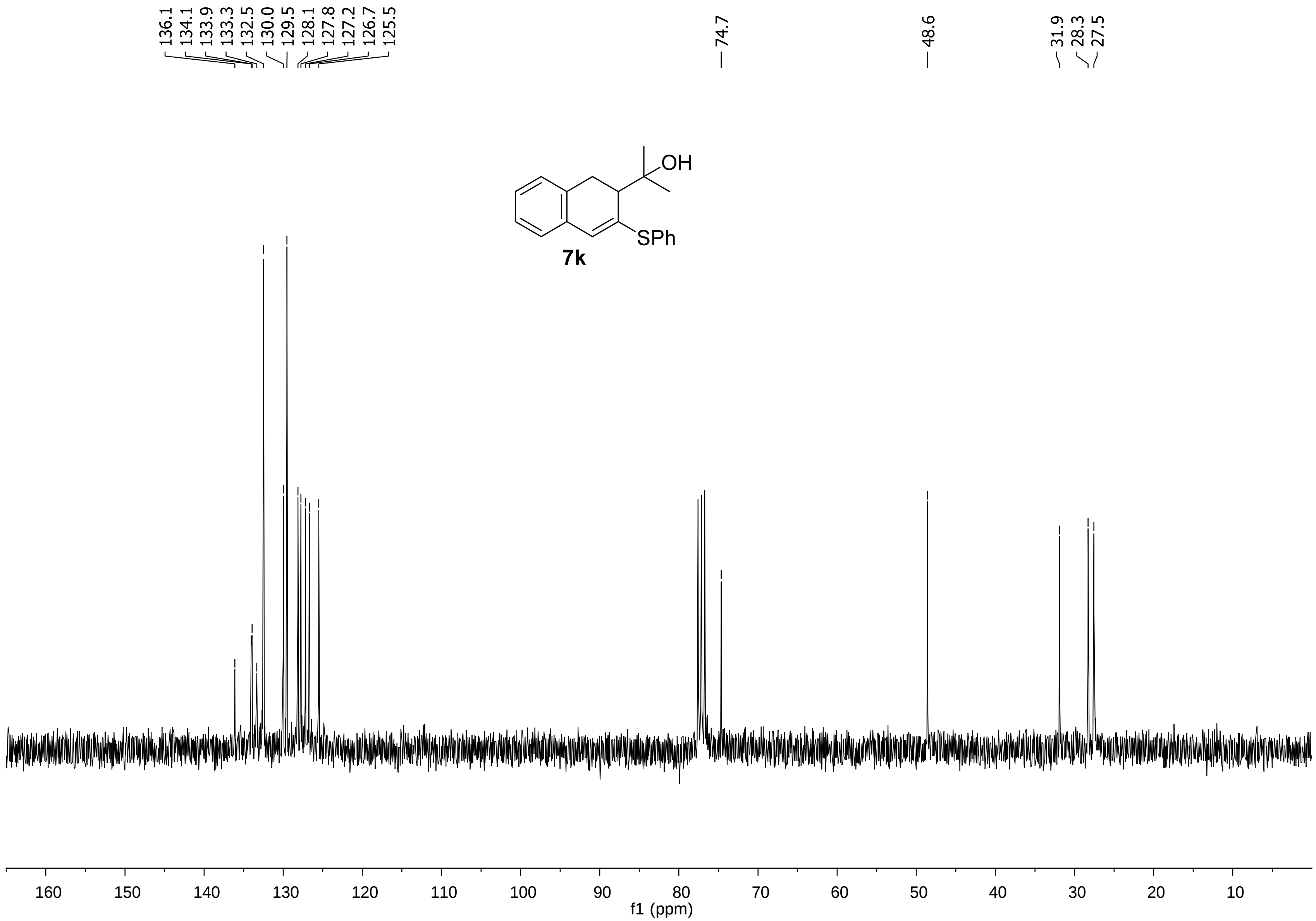
136.1
134.1
133.9
133.3
132.5
130.0
129.5
128.1
127.8
127.2
126.7
125.5
74.7
48.6
31.9
28.3
27.5
OH
SPh
7k
160
150
140
130
120
110
100
90
80
70
60
50
40
30
20
10
f1 (ppm)

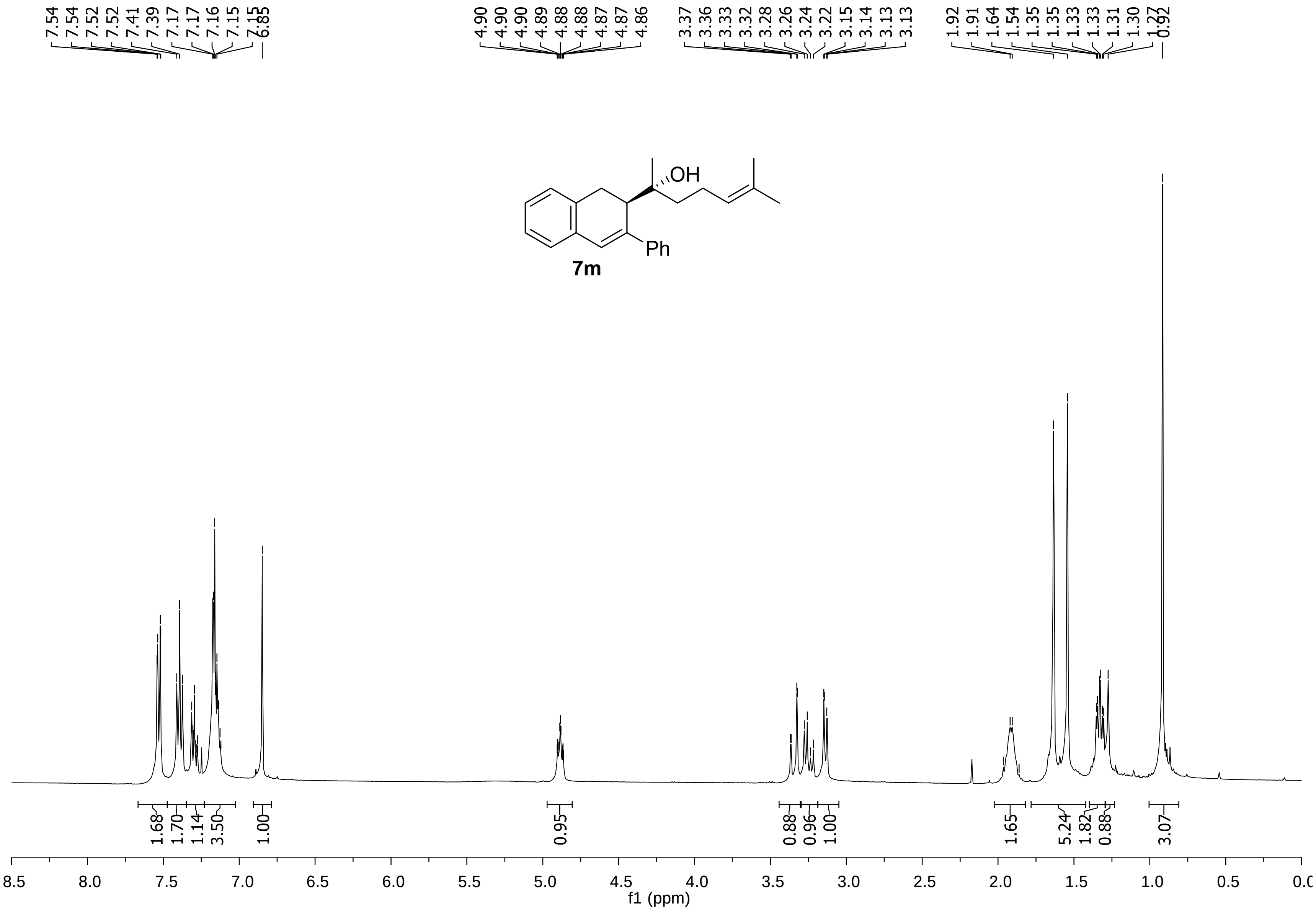

7.54
7.54
7.52
7.52
7.41
7.39
7.17
7.17
7.16
7.15
7.15
6.85
4.90
4.90
4.90
4.89
4.88
4.88
4.87
4.87
4.86
3.37
3.36
3.33
3.32
3.28
3.26
3.24
3.22
3.15
3.14
3.13
3.13
1.92
1.91
1.64
1.54
1.35
1.35
1.33
1.33
1.31
1.30
1.27
0.92
OH
Ph
7m
1.68
1.70
1.14
3.50
1.00
0.95
0.88
0.96
1.00
1.65
5.24
1.82
0.88
3.07
8.5
8.0
7.5
7.0
6.5
6.0
5.5
5.0
4.5
4.0
3.5
3.0
2.5
2.0
1.5
1.0
0.5
0.0
f1 (ppm)

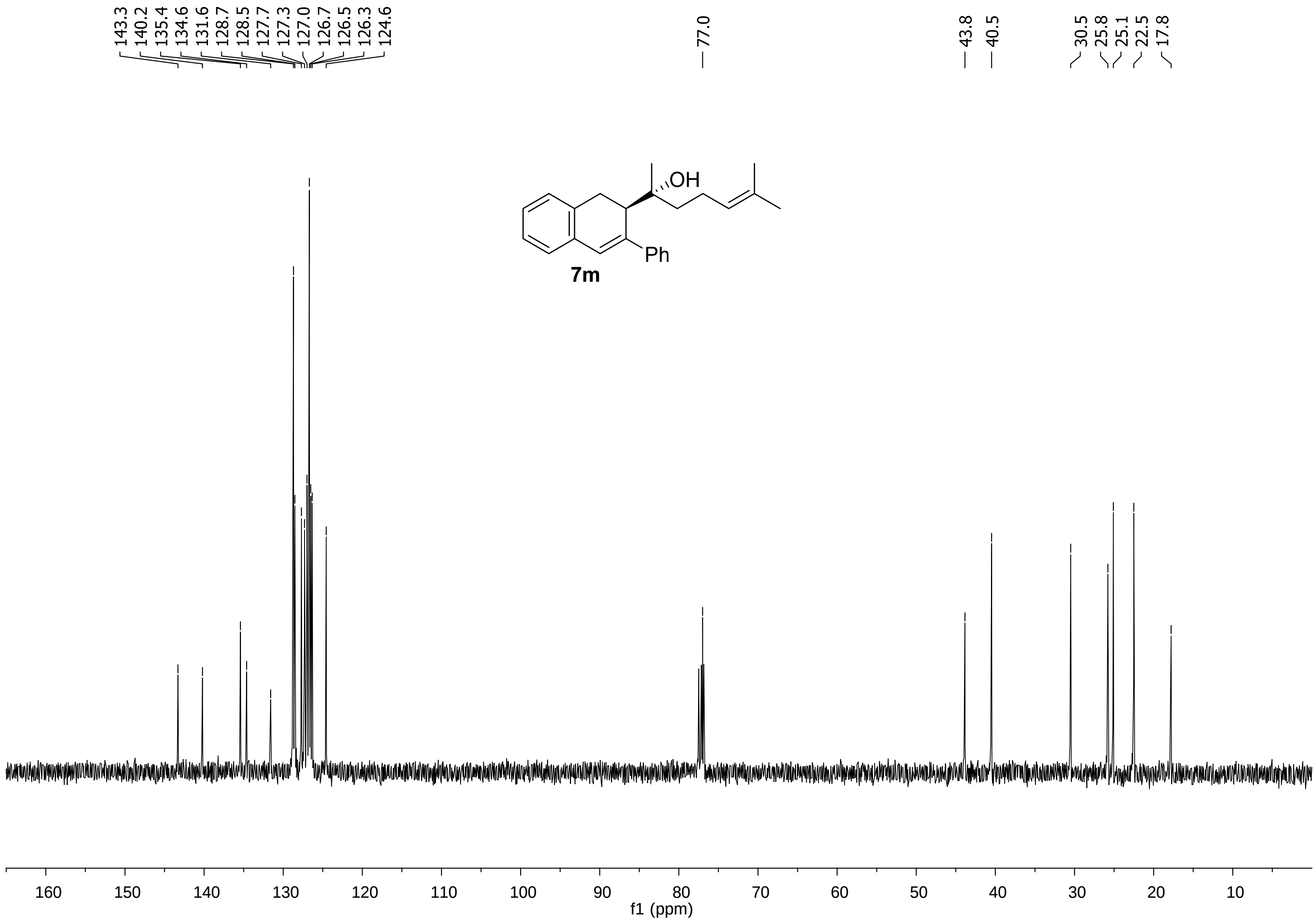
143.3
140.2
135.4
134.6
131.6
128.7
128.5
127.7
127.3
127.0
126.7
126.5
126.3
124.6
77.0
43.8
40.5
30.5
25.8
25.1
22.5
17.8
OH
Ph
7m
160
150
140
130
120
110
100
90
80
70
60
50
40
30
20
10
f1 (ppm)

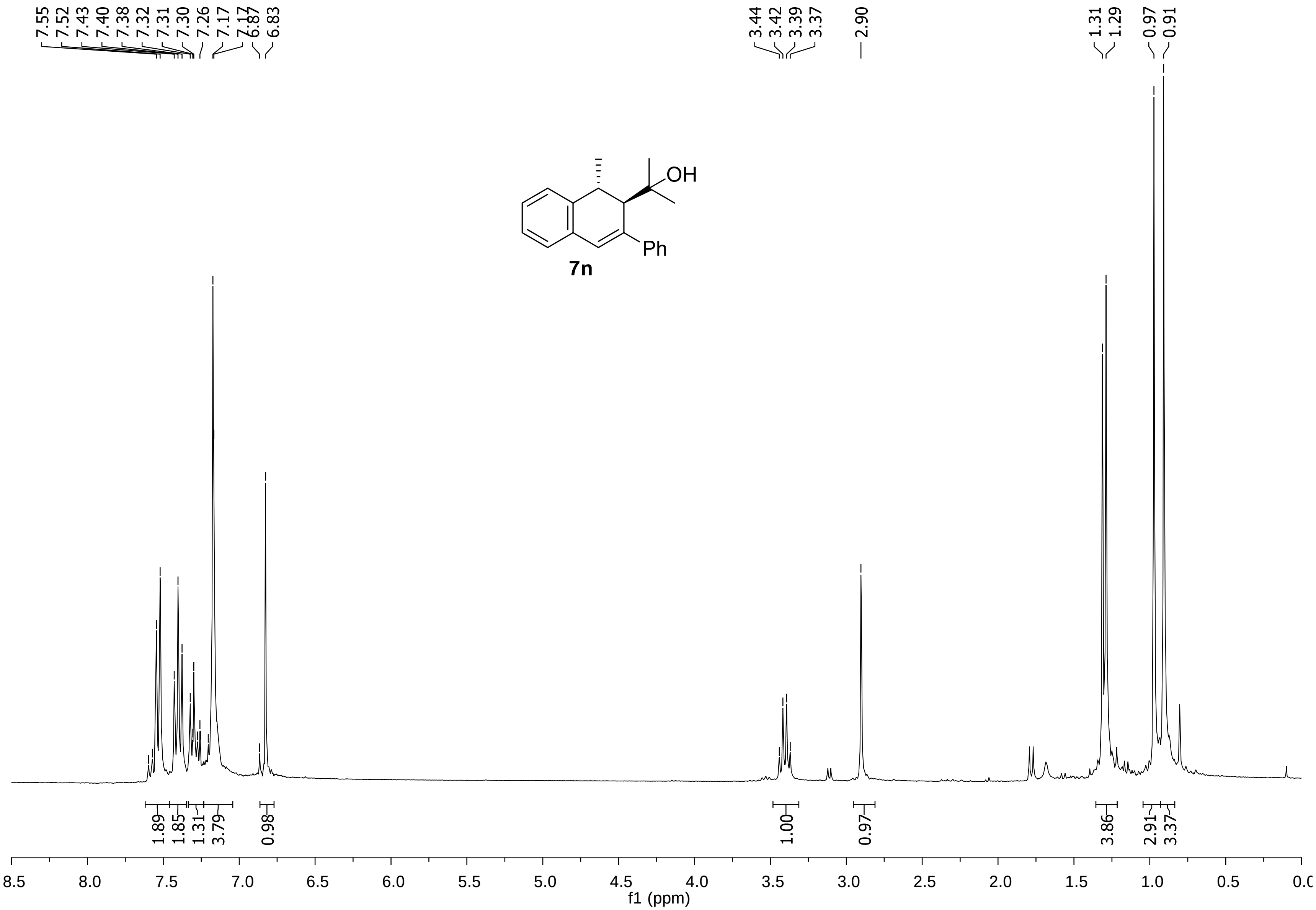
7.55 7.52 7.43 7.40 7.38 7.32 7.31 7.30 7.26 7.17 7.17 6.87 6.83
3.44 3.42 3.39 3.37
2.90
1.31 1.29
0.97 0.91
OH
Ph
7n
1.89 1.85 1.31 3.79 0.98
1.00
0.97
3.86
2.91 3.37
8.5 8.0 7.5 7.0 6.5 6.0 5.5 5.0 4.5 4.0 3.5 3.0 2.5 2.0 1.5 1.0 0.5 0.0
f1 (ppm)

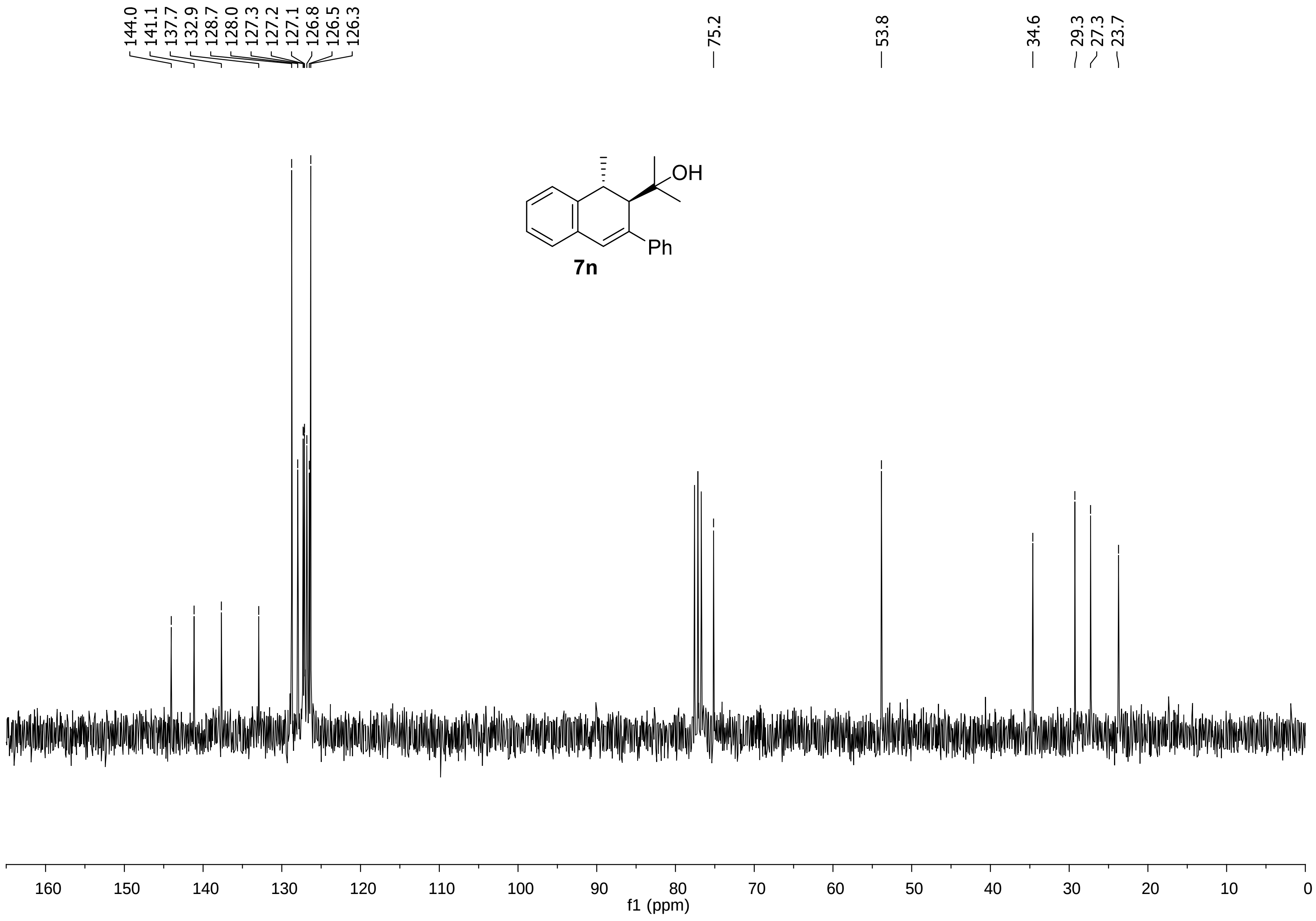
144.0
141.1
137.7
132.9
128.7
128.0
127.3
127.2
127.1
126.8
126.5
126.3
75.2
53.8
34.6
29.3
27.3
23.7
OH
Ph
7n
160
150
140
130
120
110
100
90
80
70
60
50
40
30
20
10
0
f1 (ppm)

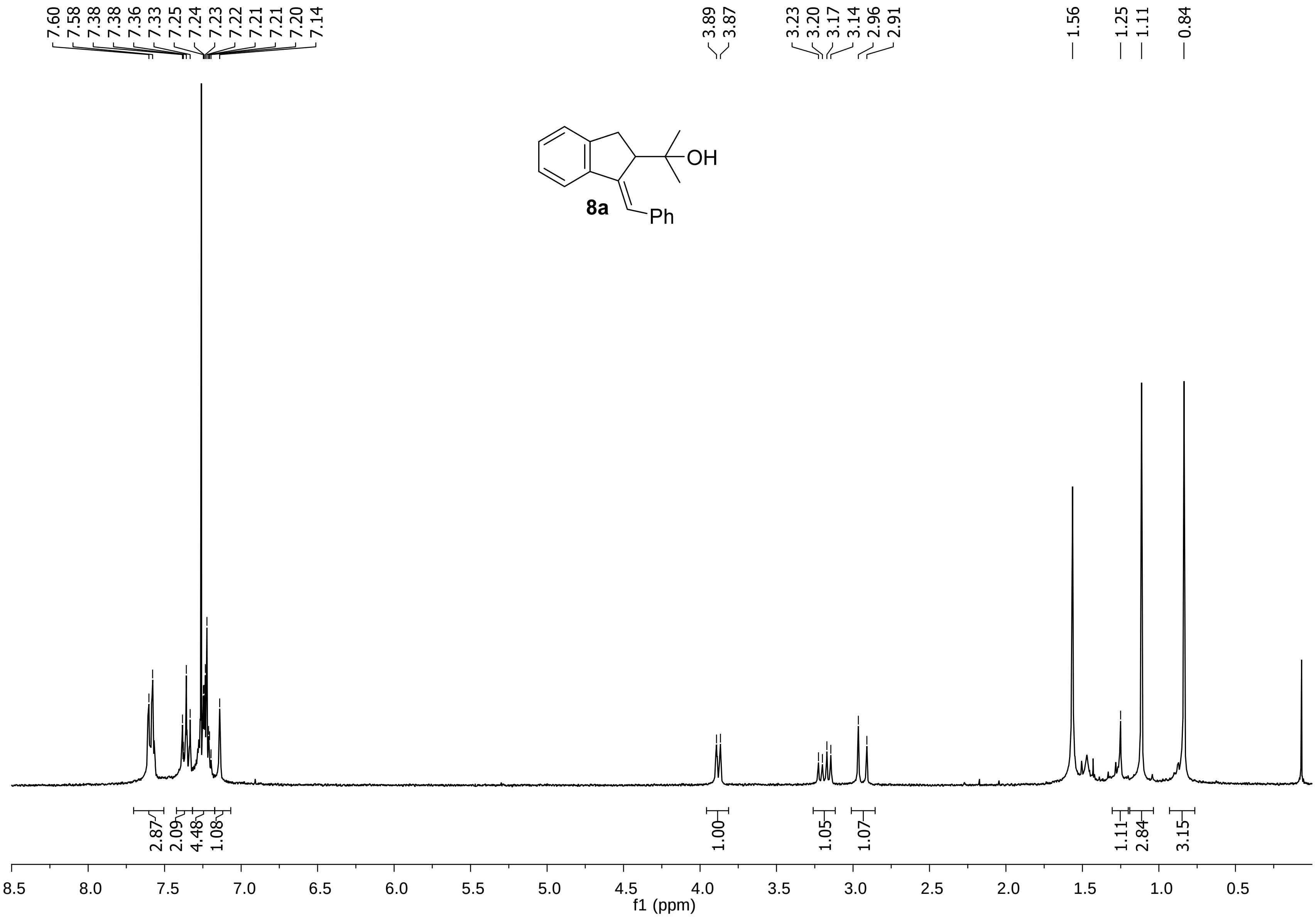
7.60 7.58 7.38 7.38 7.36 7.33 7.25 7.24 7.23 7.22 7.21 7.21 7.20 7.14
3.89 3.87
3.23 3.20 3.17 3.14 2.96 2.91
1.56
1.25 1.11
0.84
OH
8a
Ph
2.87 2.09 4.48 1.08
1.00
1.05 1.07
1.11 2.84 3.15
8.5 8.0 7.5 7.0 6.5 6.0 5.5 5.0 4.5 4.0 3.5 3.0 2.5 2.0 1.5 1.0 0.5
f1 (ppm)

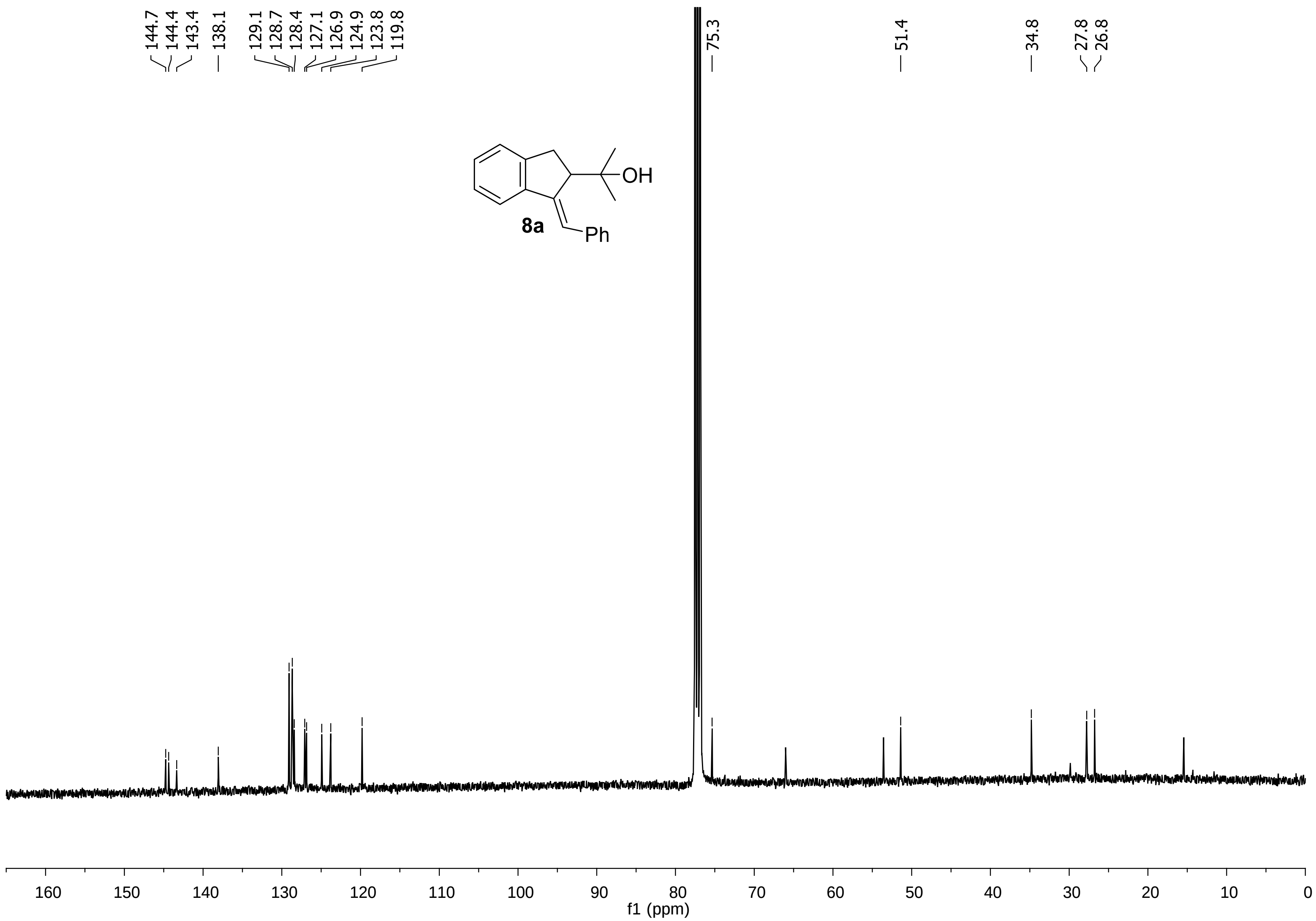
144.7
144.4
143.4
138.1
129.1
128.7
128.4
127.1
126.9
124.9
123.8
119.8
75.3
51.4
34.8
27.8
26.8
OH
8a
Ph
160 150 140 130 120 110 100 90 80 70 60 50 40 30 20 10 0
f1 (ppm)

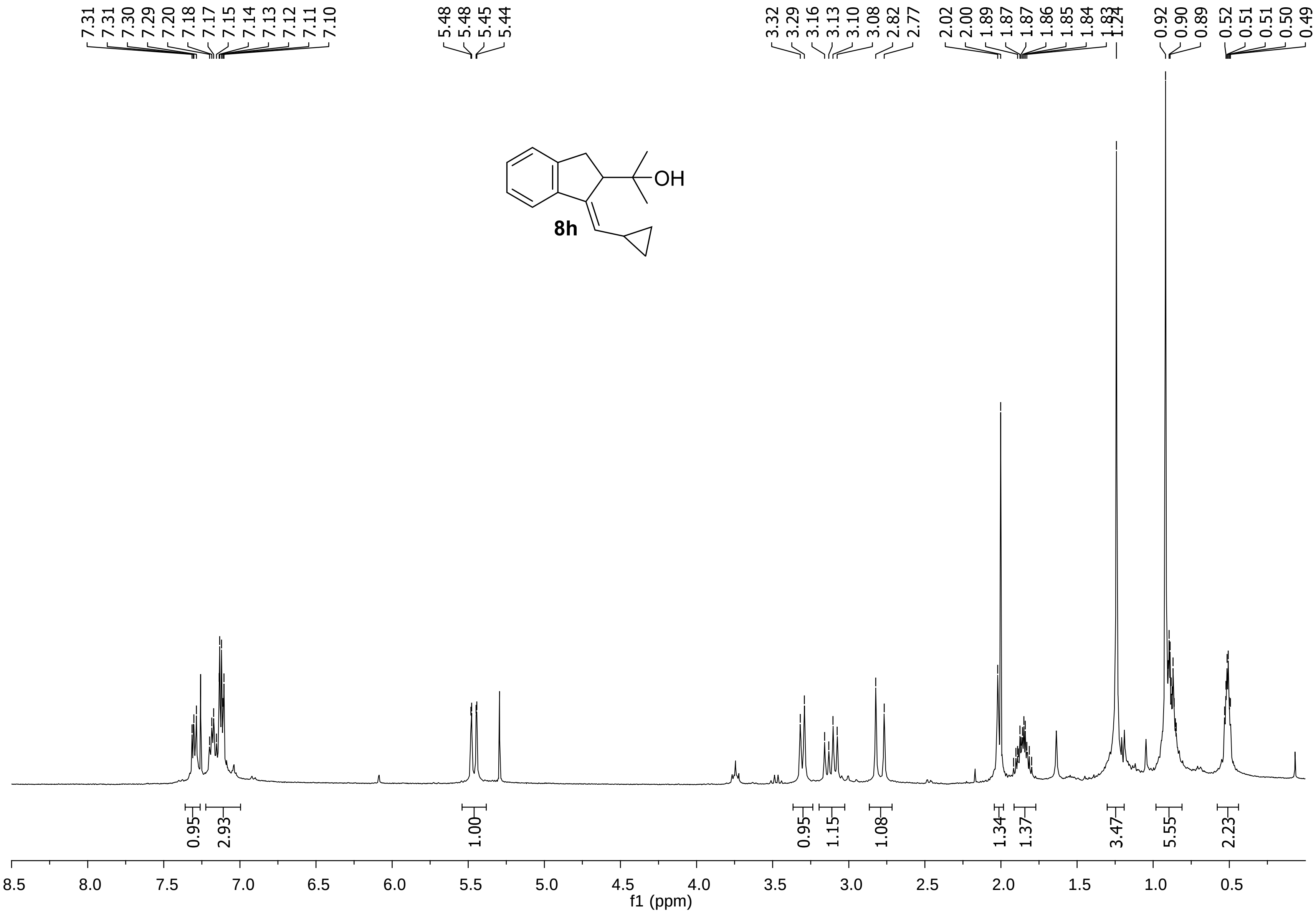
7.31 7.31 7.30 7.29 7.20 7.18 7.17 7.15 7.14 7.13 7.12 7.11 7.10
5.48 5.48 5.45 5.44
3.32 3.29 3.16 3.13 3.10 3.08 2.82 2.77
2.02 2.00 1.89 1.87 1.87 1.86 1.85 1.84 1.83 1.24
0.92 0.90 0.89 0.52 0.51 0.51 0.50 0.49
OH
8h
0.95 2.93 1.00 0.95 1.15 1.08 1.34 1.37 3.47 5.55 2.23
8.5 8.0 7.5 7.0 6.5 6.0 5.5 5.0 4.5 4.0 3.5 3.0 2.5 2.0 1.5 1.0 0.5
f1 (ppm)

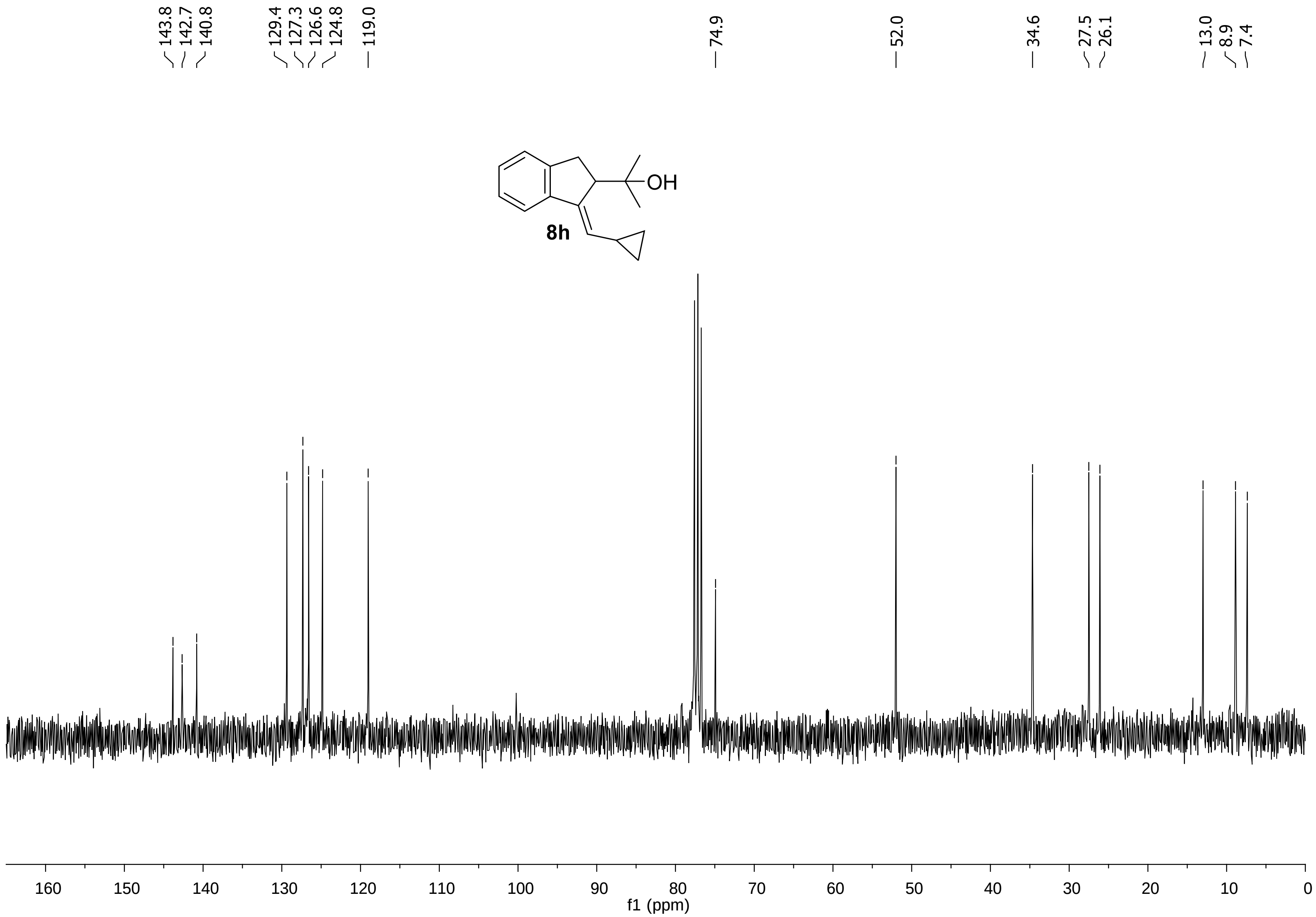
143.8
142.7
140.8
129.4
127.3
126.6
124.8
119.0
74.9
52.0
34.6
27.5
26.1
13.0
8.9
7.4
OH
8h
160
150
140
130
120
110
100
90
80
70
60
50
40
30
20
10
0
f1 (ppm)

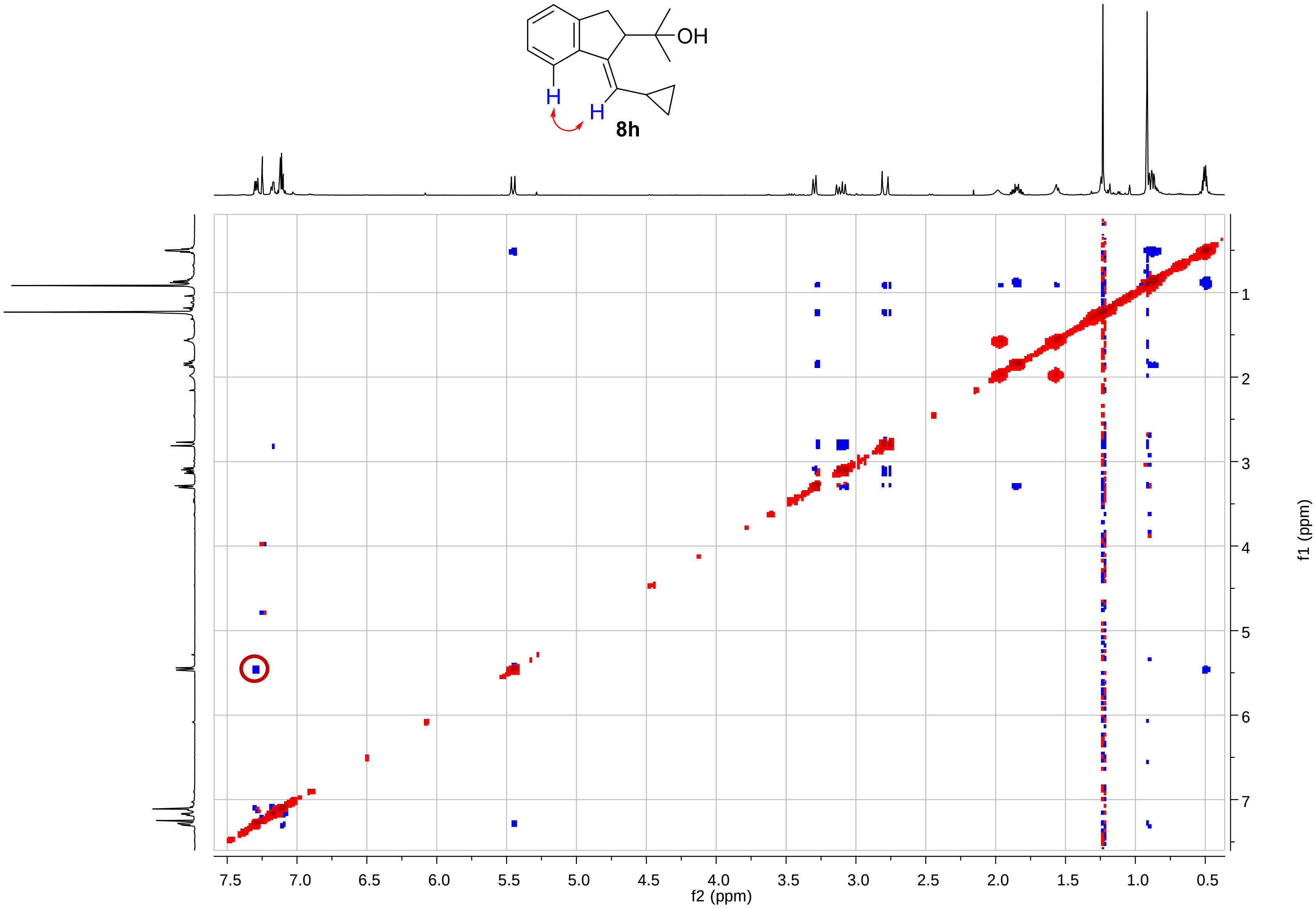
OH
H
H
8h
f2 (ppm)
f1 (ppm)

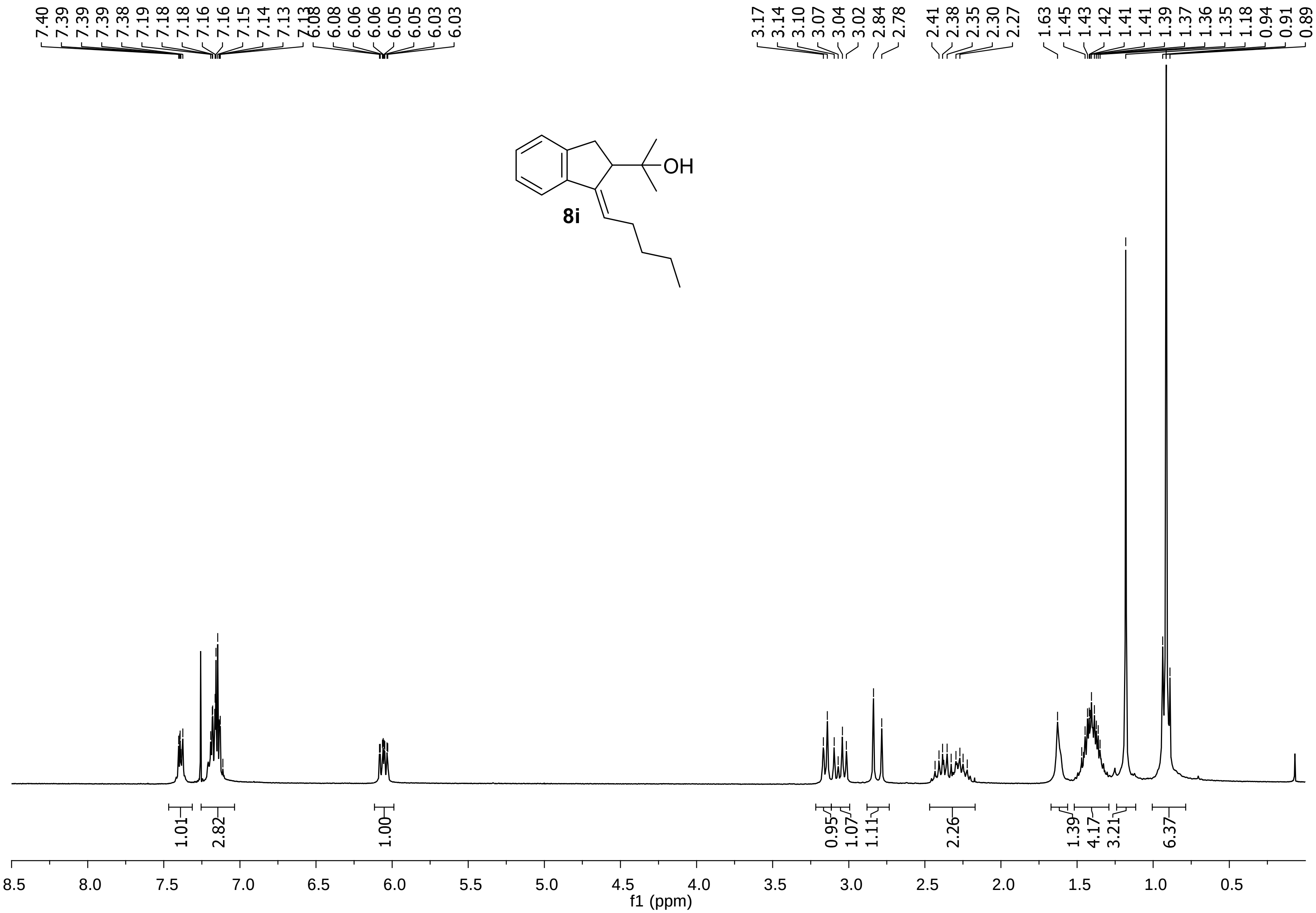
7.40 7.39 7.39 7.39 7.38 7.19 7.18 7.18 7.16 7.16 7.15 7.14 7.13 7.13 6.08 6.08 6.06 6.06 6.05 6.05 6.03 6.03
3.17 3.14 3.10 3.07 3.04 3.02 2.84 2.78 2.41 2.38 2.35 2.30 2.27 1.63 1.45 1.43 1.42 1.41 1.41 1.39 1.37 1.36 1.35 1.18 0.94 0.91 0.89
OH
8i
1.01 2.82 1.00 0.95 1.07 1.11 2.26 1.39 4.17 3.21 6.37
8.5 8.0 7.5 7.0 6.5 6.0 5.5 5.0 4.5 4.0 3.5 3.0 2.5 2.0 1.5 1.0 0.5
f1 (ppm)

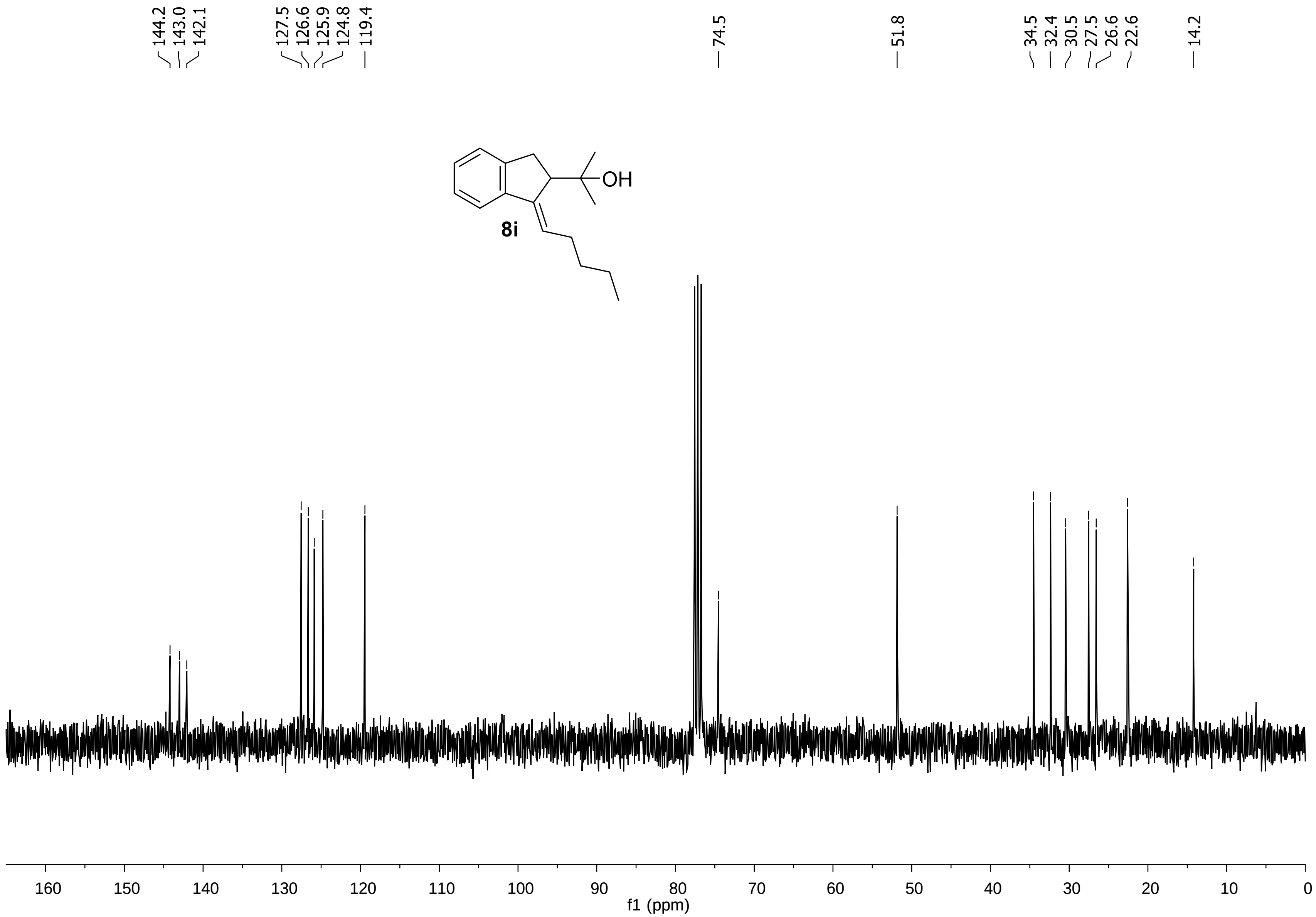
144.2
143.0
142.1
127.5
126.6
125.9
124.8
119.4
74.5
51.8
34.5
32.4
30.5
27.5
26.6
22.6
14.2
OH
8i
160
150
140
130
120
110
100
90
80
70
60
50
40
30
20
10
0
f1 (ppm)

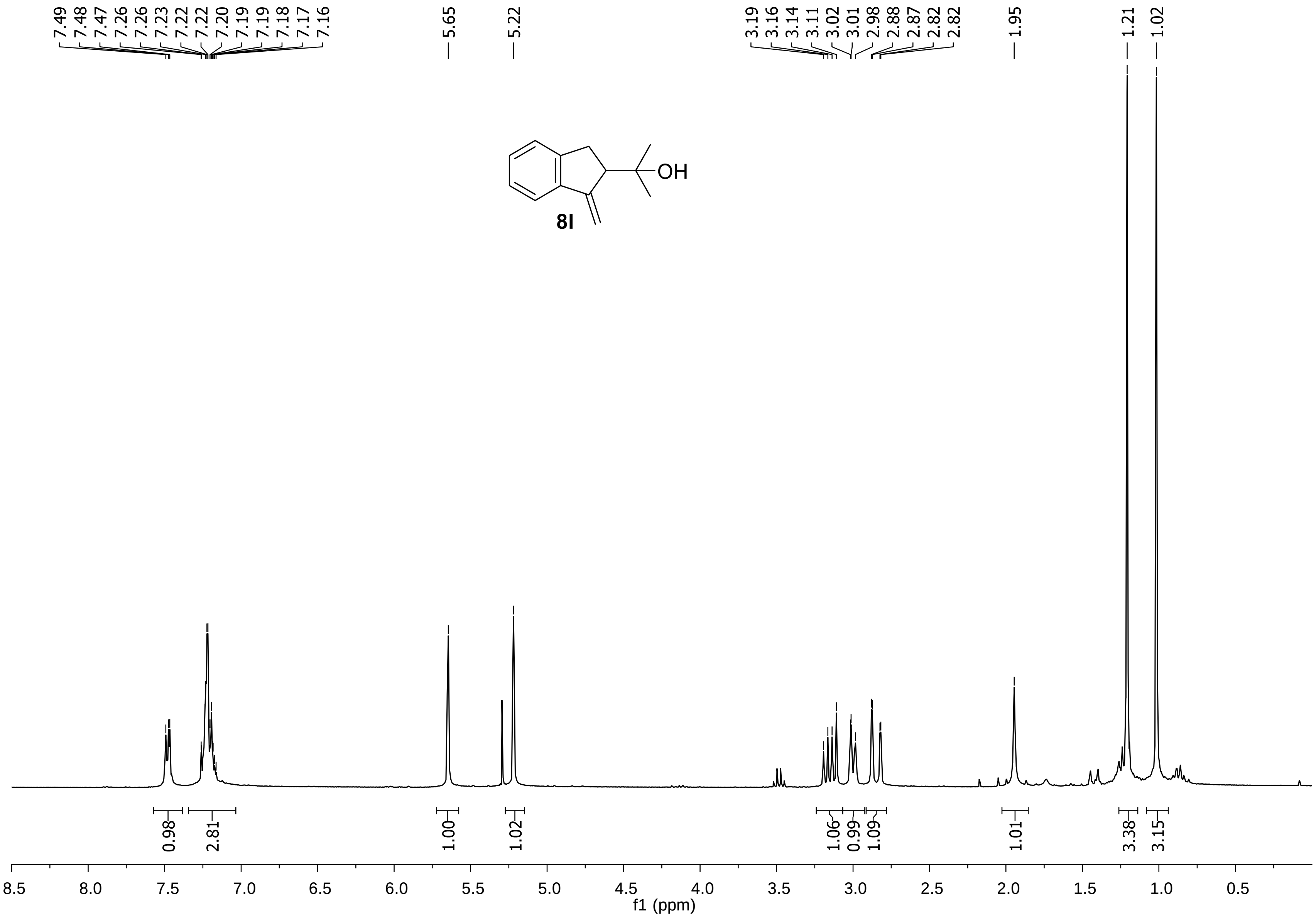
7.49 7.48 7.47 7.26 7.26 7.23 7.22 7.22 7.20 7.19 7.19 7.18 7.17 7.16
5.65
5.22
3.19 3.16 3.14 3.11 3.02 3.01 2.98 2.88 2.87 2.82 2.82
1.95
1.21 1.02
OH
8l
0.98 2.81 1.00 1.02 1.06 0.99 1.09 1.01 3.38 3.15
8.5 8.0 7.5 7.0 6.5 6.0 5.5 5.0 4.5 4.0 3.5 3.0 2.5 2.0 1.5 1.0 0.5
f1 (ppm)

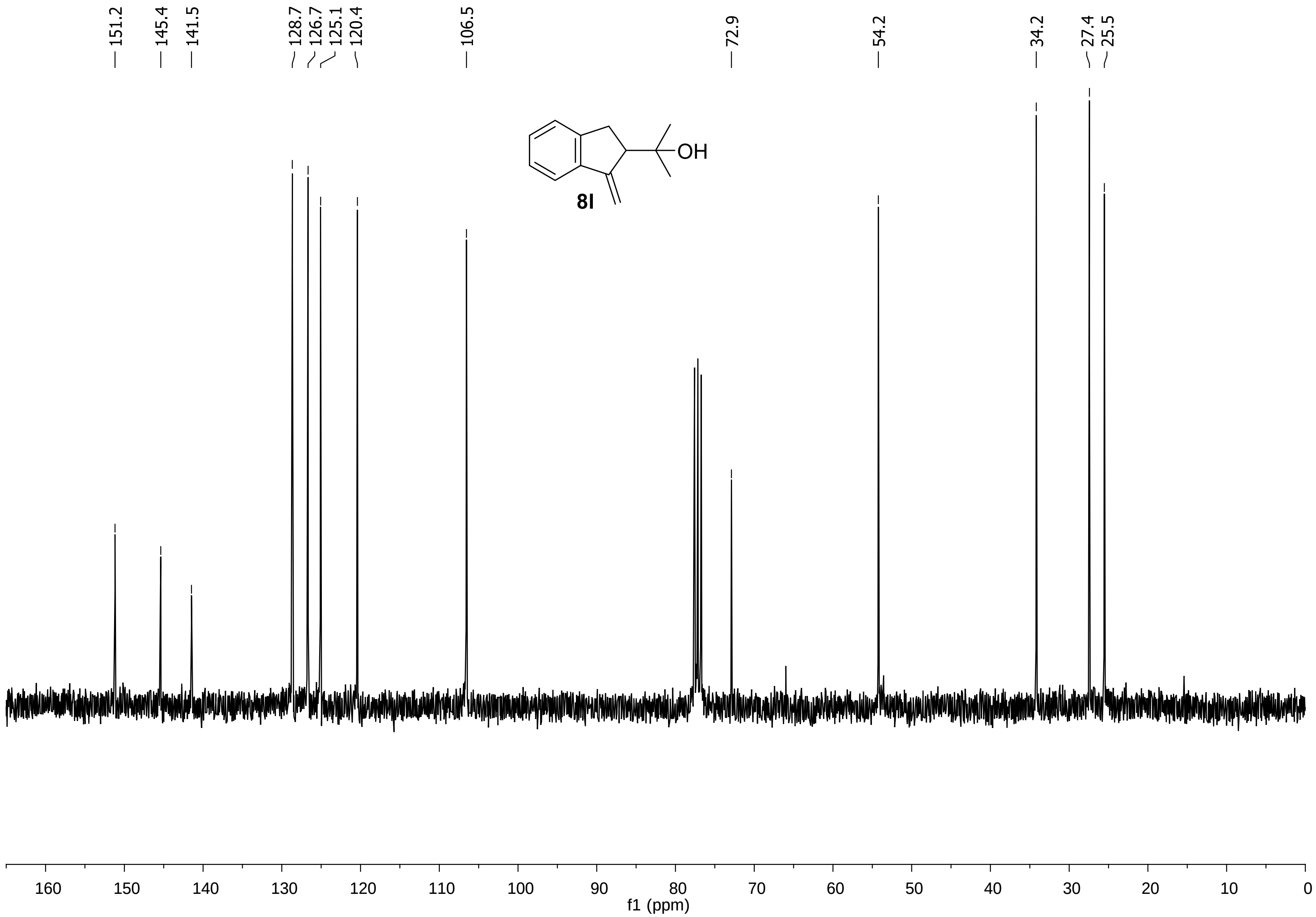
151.2
145.4
141.5
128.7
126.7
125.1
120.4
106.5
72.9
54.2
34.2
27.4
25.5
OH
8l
160 150 140 130 120 110 100 90 80 70 60 50 40 30 20 10 0
f1 (ppm)
